# Discovery of Novel Acetamide-Based Heme Oxygenase-1 Inhibitors with Potent *In Vitro* Antiproliferative Activity

**DOI:** 10.1021/acs.jmedchem.1c00633

**Published:** 2021-09-02

**Authors:** Antonino N. Fallica, Valeria Sorrenti, Agata G. D’Amico, Loredana Salerno, Giuseppe Romeo, Sebastiano Intagliata, Valeria Consoli, Giuseppe Floresta, Antonio Rescifina, Velia D’Agata, Luca Vanella, Valeria Pittalà

**Affiliations:** † Department of Drug and Health Sciences, University of Catania, 95125 Catania, Italy; ‡ Department of Analytics, Environmental & Forensics, King’s College London, Stamford Street, London SE1 9NH, U.K.; § Sections of Human Anatomy and Histology, Department of Biomedical and Biotechnological Sciences, University of Catania, 95123 Catania, Italy

## Abstract

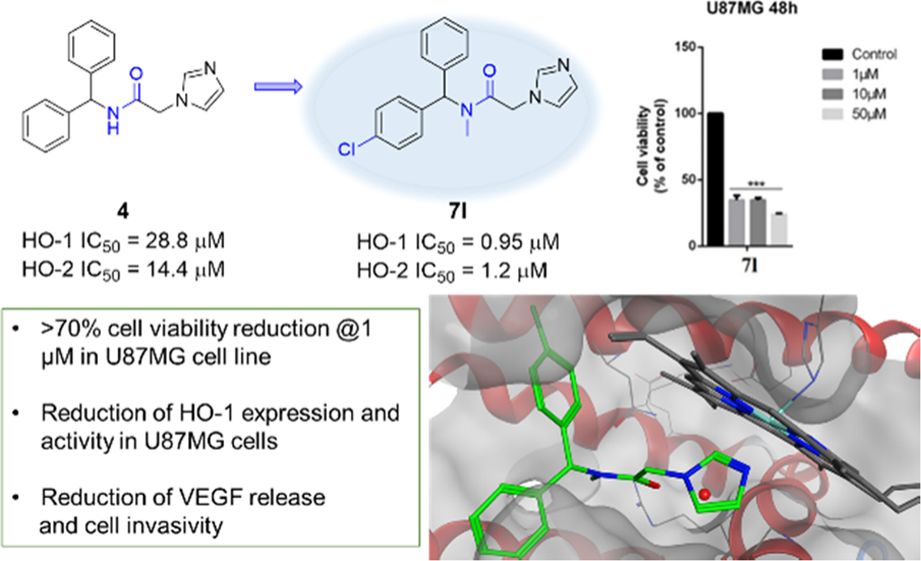

Heme oxygenase-1 (HO-1) promotes heme catabolism exercising cytoprotective roles in normal and cancer cells. Herein, we report the design, synthesis, molecular modeling, and biological evaluation of novel HO-1 inhibitors. Specifically, an amide linker in the central spacer and an imidazole were fixed, and the hydrophobic moiety required by the pharmacophore was largely modified. In many tumors, overexpression of HO-1 correlates with poor prognosis and chemoresistance, suggesting the inhibition of HO-1 as a possible antitumor strategy. Accordingly, compounds **7i** and **7l**–**p** emerged for their potency against HO-1 and were investigated for their anticancer activity against prostate (DU145), lung (A549), and glioblastoma (U87MG, A172) cancer cells. The selected compounds showed the best activity toward U87MG cells. Compound **7l** was further investigated for its in-cell enzymatic HO-1 activity, expression levels, and effects on cell invasion and vascular endothelial growth factor (VEGF) extracellular release. The obtained data suggest that **7l** can reduce cell invasivity acting through modulation of HO-1 expression.

## Introduction

**Figure 1:**
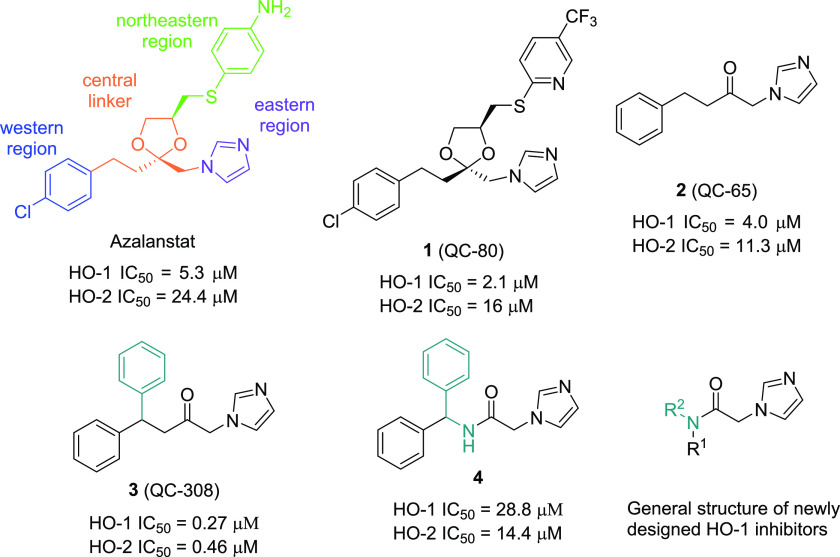
Chemical structures of azalanstat, 1 (QC-80), 2 (QC-65), 3 (QC-308), and hit compound 4.

Heme metabolism is under the tight control of a family of phase II detoxifying enzymes known as heme oxygenase (HO).^1^ HOs include heme oxygenase-1 (HO-1) and heme oxygenase-2 (HO-2) isoforms. HO-2 is a constitutive isoform and has been characterized generally in testis and the brain, where this isoform is more abundant. While HO-2 distribution remains unchanged regardless of the endogenous oxidative stress status, HO-1, whose expression is mainly under the control of the transcription factor, nuclear factor erythroid 2-related factor 2 (Nrf2) is an inducible isoform implicated in counteracting inflammation and oxidative stress responses.^1,2^ Metabolites produced upon heme breakdown, biliverdin, bilirubin, carbon monoxide, and Fe^2+^ further support HO-1 cytoprotective roles. As a result, HO-1 gained interest over the years, and its induction is valuable in several oxidative stress-dependent diseases.^3−5^ At the same time, literature reports indicate a key role of HO-1 in promoting cell survival in cancerous cells and resistance to the current anticancer therapies. Patients presenting HO-1 aberrant overexpression appear to have lesser survival chances and more unsatisfactory clinical outcomes. In fact, the detrimental role of HO-1 has been demonstrated in leukemia, glioblastoma (GBM), prostate, lung, and colon cancers.^6−12^ Also, whereas HO-1 is generally found in the cytoplasm, a different subcellular localization was detected in cancerous tissues. In fact, higher levels of nuclear HO-1 have been detected in malignant tissues than those in normal ones, which has been speculated to be strictly linked with cancer progression.^13,14^ These aspects pushed for the search of selective HO-1 inhibitors. Though growing information has been gained in the recent past to unravel the involvement of HO-1 in tumors, its pharmacological tractability as a new target remains to be elucidated. Therefore, the identification of new potent HO-1 inhibitors is desirable to gather knowledge faster. Structure–activity relationship (SAR) studies to identify new HO-1 inhibitors have been initially focused on metalloporphyrins (MPs), soon abandoned due to different side effects and subsequently on the nonporphyrin lead compound azalanstat (Figure 1).^15^

The main modification able to increase potency and selectivity occurred mainly through structural modifications on the central connecting chain or in the western region, while the northeastern region demonstrated to be not mandatory and the eastern region not prone to modifications.^16−19^

**Figure 2:**
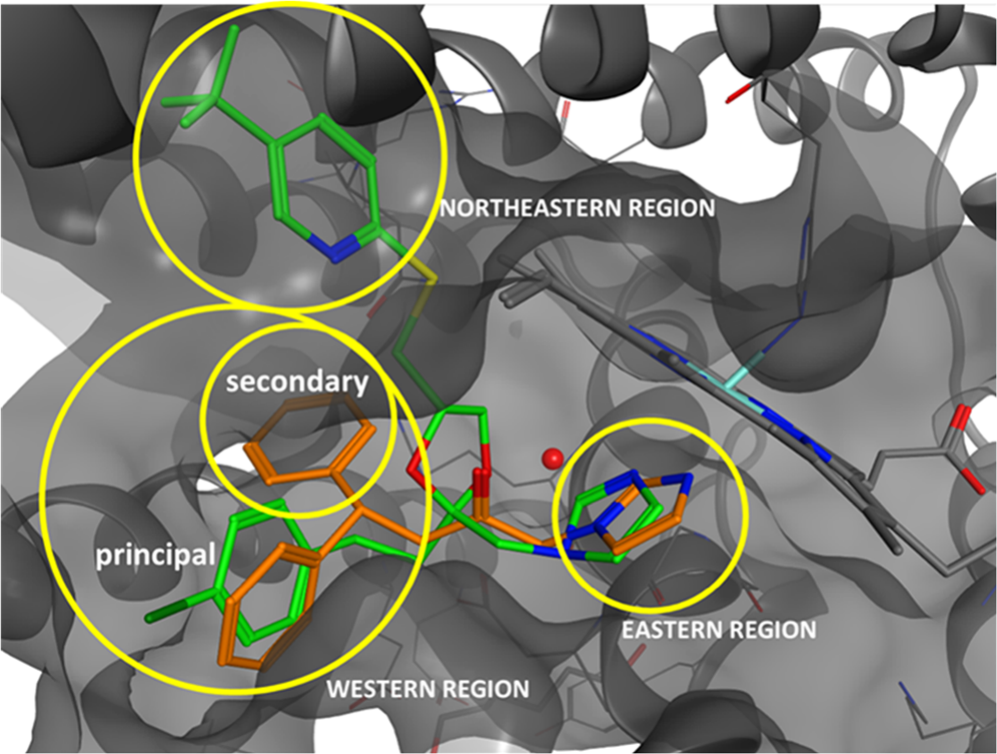
Compounds **1** (green) and **3** (orange) inside the HO-1 binding pocket.

Over the years, cocrystallization studies of HO-1 with selected inhibitors (including azalanstat and **1**–**3**; Figure 1) contributed to understanding the binding mode and the critical requirements for binding (Figure 2).^20−22^ Our research group has long been focused on the design of inhibitors oriented toward HO-1 and HO-2 using azole-based scaffolds, including the identification of the new hit compound **4** (Figure 1).^23^

Based on such premises, we designed a new series of HO-1 inhibitors with an amide function in the central connecting chain. The obtained compounds (Table 1) behaving as potent HO-1 inhibitors have been screened for their antiproliferative activity and HO-1 expression levels against a small panel of cancer cell lines, showing the best activity toward the GBM cell line U87MG. This exploration also identified compound **7l** as suitable for further investigating its in-cell enzymatic HO-1 activity and its effects on cell invasion. Encouraging results obtained on compound **7l** will pave the way to study in depth its pharmacological profile.

**Table 1:**
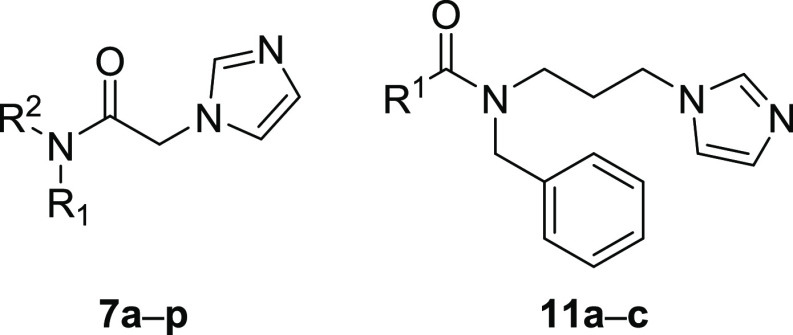
Experimental IC_50_ Values (μM) of Compounds **7a–p** and **11a–c** toward HO-1 and HO-2

			IC_50_ (μM)		
compd	R^1^	R^2^	HO-1	HO-2	SI (HO-2/HO-1)
**7a**	Ph	H	110.0 ± 3.1	ND	
**7b**	Ph	CH_3_	2.50 (mM)	ND	
**7c**	3-BrPh	H	35.71 ± 1.20	ND	
**7d**	4-IPh	H	8.34 ± 0.21	ND	
**7e**	4-IPh	CH_3_	96.14 ± 1.44	ND	
**7f**	PhCH_2_	H	57.60 ± 2.38	ND	
**7g**	PhCH_2_	CH_3_	50.63 ± 1.84	ND	
**7h**	PhCH_2_	PhCH_2_	64.80 ± 1.56	ND	
**7i**	Ph(Ph)CH	CH_3_	0.90 ± 0.07	0.90 ± 0.05	1
**7j**	C_5_H_9_(Ph)CH	CH_3_	11.43 ± 0.97	ND	
**7k**	C_6_H_11_(Ph)CH	CH_3_	26.84 ± 1.61	ND	
**7l**	4-ClPh(Ph)CH	CH_3_	0.95 ± 0.04	1.2 ± 0.07	1.3
**7m**	3-BrPh(Ph)CH	CH_3_	0.90 ± 0.03	1.1 ± 0.04	1.2
**7n**	4-IPh(Ph)CH	CH_3_	0.95 ± 0.09	45.89 ± 1.67	48.3
**7o**	PhCH_2_OPh(Ph)CH	CH_3_	1.20 ± 0.11	11.19 ± 0.18	9.3
**7p**	4-BrPhCH_2_OPh(Ph)CH	CH_3_	8.0 ± 0.39	24.71 ± 0.14	3.1
**11a**	CH_3_		224.0 ± 8.10	ND	
**11b**	Ph		31.0 ± 1.74	ND	
**11c**	PhCH_2_		31.99 ± 1.48	ND	
**4**^b^	Ph(Ph)CH	H	28.8 ± 1.41	14.4 ± 0.9	0.5
Azalanstat^b^			5.30 ± 0.4	24.40 ± 0.8	4.6

a Data are reported as IC_50_ values in μM ± standard deviation (SD). Values are the mean of triplicate experiments.

b Data are from ref (23).

## Results and Discussion

### Rational Design

The classical pharmacophore for HO-1 inhibition consists of (i) an iron(II)-binding group that coordinates Fe^2+^ in the HO-1 active site, (ii) a hydrophobic portion, and (iii) a central spacer connecting the two groups. This pharmacophore pattern has been investigated in the past years to derive a novel series of HO-1 inhibitors and will be herein further exploited.^16−18^ Moreover, cocrystallization studies performed with compound **3** highlighted the presence of an additional smaller secondary hydrophobic pocket in HO-1 and HO-2.^21^ This information allowed us to explain the 15-fold higher inhibitory potency toward HO-1 of **3** with respect to its monophenyl analogue **2** (0.27 *vs* 4.0 μM, respectively; Figure 1). This “double-clamp” binding mode has been poorly exploited in the search for more powerful HO-1 inhibitors. Substituents in the aromatic rings can help fine-tune both the potency and the selectivity of the resulting compounds over HO-1 or HO-2.

SAR studies performed on HO-1 inhibitors revealed that the central connecting chain could contain heteroatoms such as sulfur, oxygen, hydroxyl groups, and carbonyl functions.^16^ However, insufficient information has been reported on the tolerability of an amide function in the central connecting chain. The ability to establish critical hydrogen-bonding interactions and the consequent increase in polarity can affect the pharmacological profile of compounds possessing this functional group. Thereby, amide functional groups represent one of the most easily found structural motifs in marketed drugs and drug candidates.^24^ Recently, our research group reported the synthesis of compound **4** (Figure 1) and its IC_50_ values toward both enzymatic isoforms.^23^ Insertion of the amide function in the central connecting chain increased the HO-1 IC_50_ value from 0.27 μM for **3** to 28.8 μM for compound **4**; besides, a slight preference for the constitutive isoform was detected (HO-2 IC_50_ = 14.4 μM). Nevertheless, the amide function’s chemical versatility in medicinal chemistry and the easy-to-synthesize compounds with this functional group are appealing. To expand the SAR studies of HO-1 inhibitors, we chose compound **4** as the hit compound to design novel amide-imidazole-based HO-1 inhibitors (Table 1). Five main strategies have been pursued: (i) structural simplification by removal of the secondary phenyl ring, (ii) shortening or elongation of the central connecting chain, (iii) substitution of the nitrogen atom on the amide function to obtain tertiary amides, (iv) inversion of the amide bond, and (v) insertion of substituents on one phenyl ring or its removal in favor of the saturated cycles to explore the steric and electronic requirements of the smaller secondary hydrophobic pocket.

### Chemistry

**Scheme 1:**
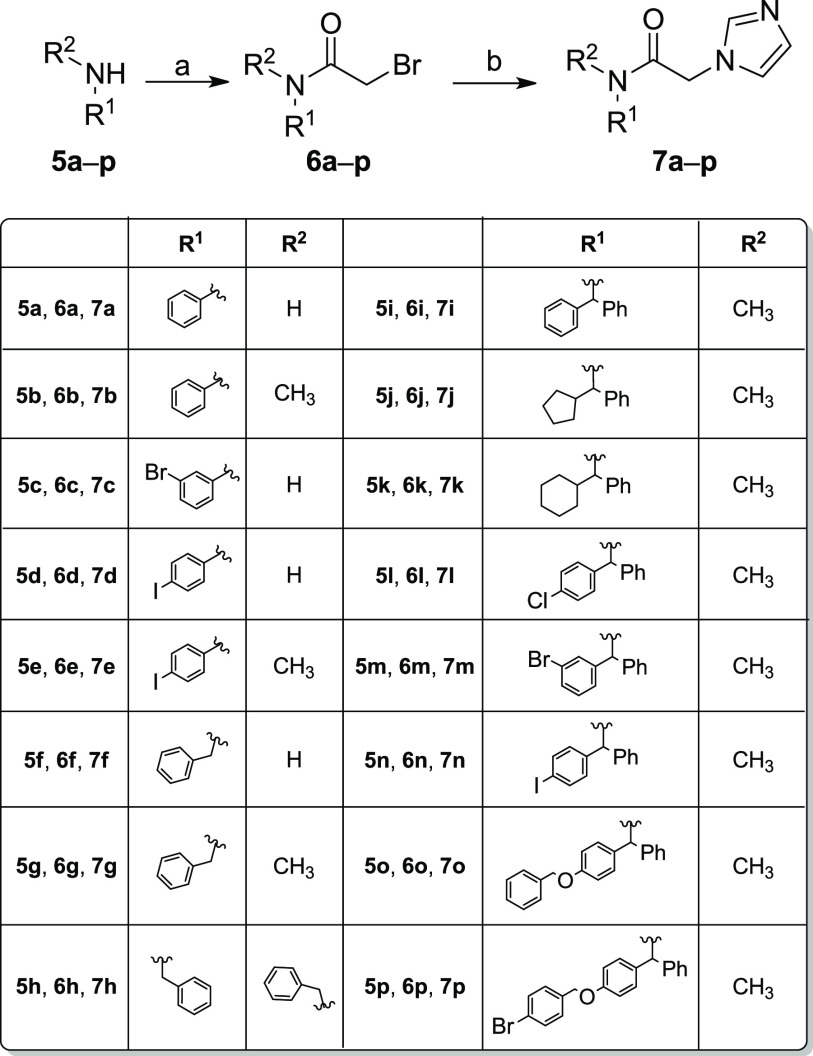
Reagents and Conditions (a) α-Bromoacetyl bromide, TEA, dry CH_3_CN, room temperature, 3 h; and (b) imidazole, K_2_CO_3_, dry DMF, room temperature, 2 h or 80% NaH in oil dispersion, dry THF, room temperature, 16 h.

Final compounds **7a**–**p** have been synthesized as depicted in Scheme 1. The first step involves the reaction of the appropriate primary or secondary amines **5a**–**p** with bromoacetyl bromide and triethylamine (TEA) in dry acetonitrile at room temperature for 3 h, affording the *N*-mono or *N*,*N*-disubstituted *α*-bromo-acetamide intermediates **6a**–**p**. The last step is an aliphatic nucleophilic substitution of compounds **6a**–**p** with imidazole. For compounds **7a**, **7c**–**d**, **7f**–**g**, **7i**, and **7o**, the reaction was conducted in dry DMF and K_2_CO_3_ at room temperature for 2 h. However, low reaction yields have been obtained with this synthetic strategy. To scale up the reaction yield and obtain larger quantities of compounds intended for biological tests, a more efficient base able to fully deprotonate the nitrogen atom of imidazole has been chosen for the nucleophilic substitution. Therefore, compounds **7b**, **7e**, **7h**, **7j**–**n**, and **7p** have been synthesized using an excess of NaH 80% in oil dispersion as base and dry tetrahydrofuran (THF) as reaction solvent at room temperature for 16 h.

**Scheme 2:**
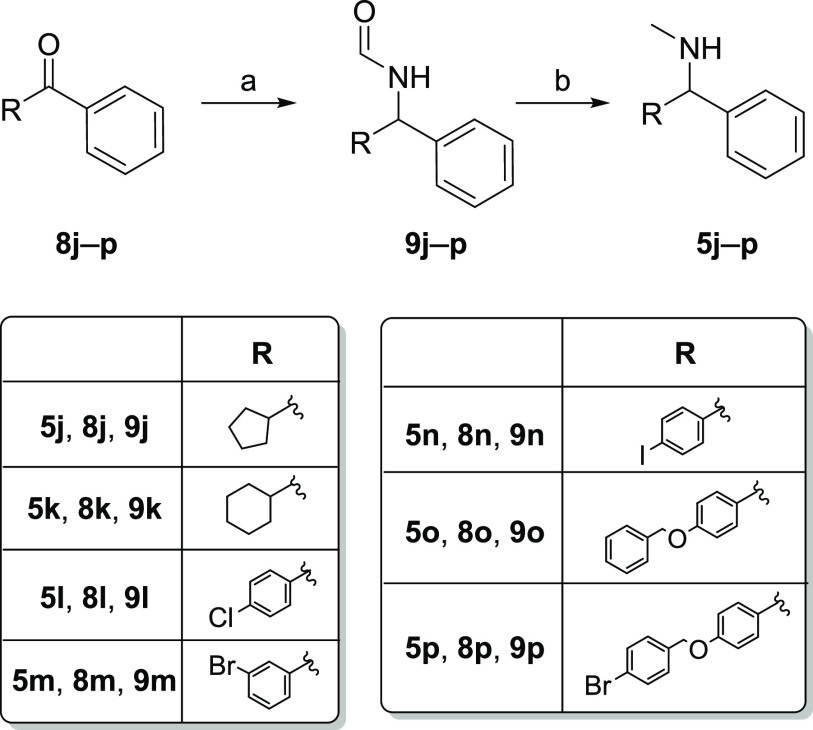
Reagents and Conditions (a) H_2_NCHO, HCOOH, MW, 150 W, 150 psi, 170 °C, 1.5 h; and (b) LiAlH_4_ in THF 1 M, dry THF, reflux, 2 h or DIBAL-H in 1 M *n*-hexane, THF, reflux, 3.5 h, and then room temperature, 16 h.

Amines **5j**–**p** have been synthesized employing the Leuckart reaction, a synthetic procedure in which an appropriate starting ketone **8j**–**p** is converted into the corresponding *N*-methyl amine derivative by reductive amination of the carbonyl function (Scheme 2). The reaction classically requires two steps: in the first one, ketones **8j**–**p** were treated with formamide and formic acid at 170 °C for 18 h. To reduce the reaction time, a microwave-assisted procedure has been developed, and the best results have been obtained at 170 °C for 90 min. The formamide function of derivatives **9j**–**p** has been subsequently reduced to a methylamino group using LiAlH_4_ in THF 1 M (for the synthesis of compounds **5j**–**k** and **5o**–**p**) or diisobutylaluminum hydride (DIBAL-H) in 1 M *n*-hexane (for the synthesis of compounds **5l**–**n**).

**Scheme 3:**
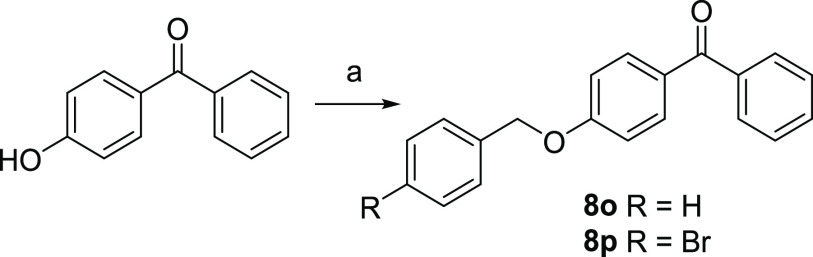
Reagents and Conditions (a) Appropriate benzyl bromide, K_2_CO_3_, KI, acetone, reflux, 3 h.

4-Benzyloxyketones **8o**–**p** were synthesized as depicted in Scheme 3. The reaction involves the etherification of 4-hydroxybenzophenone with the appropriate benzyl bromide in refluxing acetone with K_2_CO_3_ and KI as catalysts for 3 h.

**Scheme 4:**
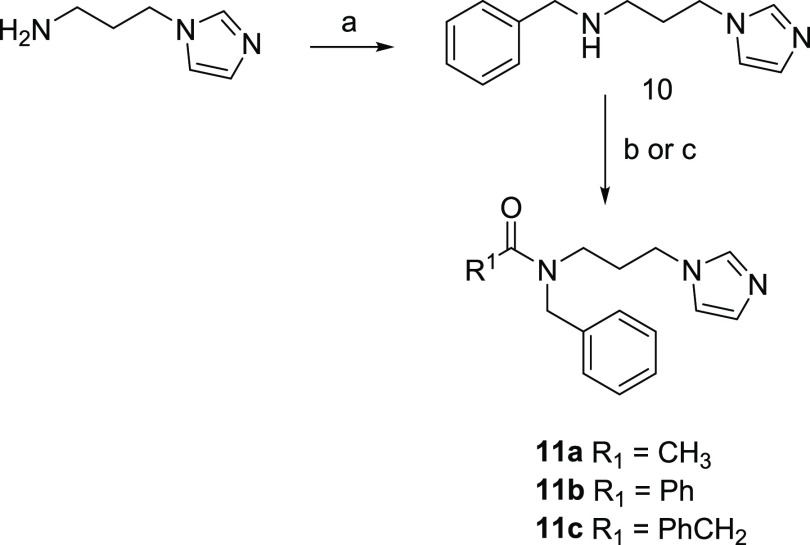
Reagents and Conditions (a) Benzaldehyde, CH_3_CO_2_Na, activated 3 Å molecular sieves, dry CH_3_OH, room temperature, 12 h, then NaBH_4_, room temperature, 3 h; (b) acetic anhydride, TEA, 4-dimethylaminopyridine (DMAP), dry CH_2_Cl_2_, 0 °C, 10 min, then room temperature, 12 h; and (c) appropriate acyl chloride, TEA, dry CH_2_Cl_2_, room temperature, 12 h.

Scheme 4 reports the synthesis of final compounds **11a**–**c**. The synthesis of these compounds required two steps: a reductive alkylation of 3-(1*H*-imidazol-1-yl)propan-1-amine with benzaldehyde and NaBH_4_ in dry methanol afforded intermediate **10**, which was condensed with acetic anhydride or an appropriate acyl chloride in dry dichloromethane, affording compounds **11a** and **11b**,**c**, respectively.

All compounds have been characterized by IR, ^1^H NMR, melting point, and elemental analysis; the final compounds have also been characterized by ^13^C NMR. Intermediates and final compounds possessing a tertiary amide functional group exist as an *E*/*Z* mixture, as evidenced by NMR spectra. The steric hindrance around the amide bond and the partial nature of the C–N double bond do not grant the free rotation of the different parts of the molecule around a single bond, giving rise to “*E*/*Z*” isomers. From NMR spectra, it has been possible to highlight this phenomenon by doubling specific identifying peaks, such as the methyl group linked to the nitrogen atom, CH_2_, or imidazole CH groups.

### HO Inhibition and SAR Analysis

Compounds **7a**–**p** and **11a**–**c** were tested to evaluate their inhibitory properties toward HO-1 obtained from rat spleen microsomal fractions. Data are expressed as IC_50_ (μM) and are summarized in Table 1. Compounds with an HO-1 IC_50_ ≤ 8 μM have also been screened to inhibit the constitutive HO-2, which was obtained from rat brain microsomal fractions. IC_50_ values of **1** and **4** are also reported. Based on the results obtained by the enzymatic assays, SARs can be built. Shortening of the central connecting chain of compound **4** from four to three atoms afforded anilide compounds **7a**–**e**. This specific structural modification does not allow the presence of the secondary phenyl ring. Except for **7d**, which displayed an HO-1 IC_50_ value of 8.34 μM, all anilides exhibited poor inhibitory activity, and all of them are less potent than hit compound **4**. Furthermore, N-methylation of anilides **7a** and **7d** afforded **7b** and **7e**, respectively, showing a strong drop in activity. To verify if the “double-clamp” binding mode also plays a role in this class of amide derivatives, we removed the second phenyl ring of compound **4** affording **7f**, which showed a 2-fold decrease in potency with respect to **4** (57.60 *vs* 28.8 μM, respectively). N-Methylation of **7f** (compound **7g**) did not produce a significant improvement of the inhibitory potency. A bulkier substituent such as a benzyl moiety linked to the nitrogen atom of the amide function (compound **7h**) showed comparable activity to its *N*-methyl analogue **7g** (64.80 *vs* 50.63 μM, respectively). From these results, we can assume that shortening of the central connecting chain or deletion of the secondary phenyl ring reduces the affinity. In light of the previous considerations, we then decided to add a small substituent at the nitrogen atom of compound **4**. The corresponding *N*-methyl derivative **7i** displayed an IC_50_ value of 0.9 μM and is about 32-fold more potent than the parent compound **4**. Therefore, compound **7i** has been considered the lead compound for the design and synthesis of derivatives **7j**–**p**. Interestingly, all N-methylated **7i** derivatives improved potency, showing an opposite trend than anilide derivatives **7b** and **7e**. Considering that the two hydrophobic pockets of the HO-1 enzyme seem smaller than the HO-2 ones, we can speculate that both enzymes will accommodate bulky hydrophobic moieties differently and with different affinities.^25^ Consequently, we removed one phenyl ring in favor of a saturated cyclopentyl or cyclohexyl one (compounds **7j** and **7k**, respectively). However, both compounds showed slightly reduced inhibitory activity against HO-1; specifically, **7j** displayed a value of 11.43 μM, whereas **7k** exhibited a value of 26.84 μM being 13- and 30-fold less potent than compound **7i**, respectively. Insertion of electron-withdrawing or electron-donating groups in one phenyl ring led to compounds **7l**–**p**. Halogen-substituted compounds **7l**–**n** showed inhibitory potencies perfectly comparable with that of the unsubstituted compound **7i**. A benzyloxy or 4-bromobenzyloxy moiety at the 4-position of one phenyl ring of **7i** afforded compounds **7o** and **7p**, respectively. Both compounds can inhibit the HO-1 enzyme, although at different levels. Indeed, **7o** has an inhibitory potency similar to **7i** (1.2 *vs* 0.9 μM), whereas **7p** is about 6.5- and 9-fold less potent than **7o** and **7i**, respectively (8.0 *vs* 1.2 μM and 0.9 μM). HO-1 inhibition data for compounds **7i**-**p** overall reconfirm the importance of the secondary hydrophobic pocket and the so-called “double-clamp” binding mode, as already highlighted by compound **3**.

Elongation of the central connecting chain to five atoms and concomitant inversion of the amide bond led to the design of compounds **11a**–**c** maintaining a benzyl substituent at the nitrogen atom of the amide bond.

The lipophilic portion of the molecule has been changed from an acetyl group to a more lipophilic phenyl or phenylmethyl substituents. The *N*-acetyl derivative **11a** resulted in being almost inactive, whereas compounds **11b** and **11c** showed IC_50_ values comparable to the hit compound **4** (Table 1). Interestingly, these results confirmed that the HO-1 enzyme less tolerates substituents linked to the *N*-atom bulkier than a methyl group.

The most potent HO-1 inhibitors of this series, possessing an IC_50_ ≤ 8 μM, have also been tested to evaluate their selectivity toward the HO-2 isozyme. Compounds **7i**, **7l**, and **7m** showed superimposable HO-1 and HO-2 IC_50_ values, and compounds **7o** and **7p** are 9- and 3-fold more selective for the inducible isoform, respectively (1.20 *vs* 11.19 μM for **7o**, 8.0 *vs* 24.71 μM for **7p**). The most selective compound of this series, 4-iodo-monosubstituted derivative **7n**, is about 48-fold more selective for HO-1 (0.95 *vs* 45.89 μM).

### Molecular Modeling Studies

**Figure 3:**
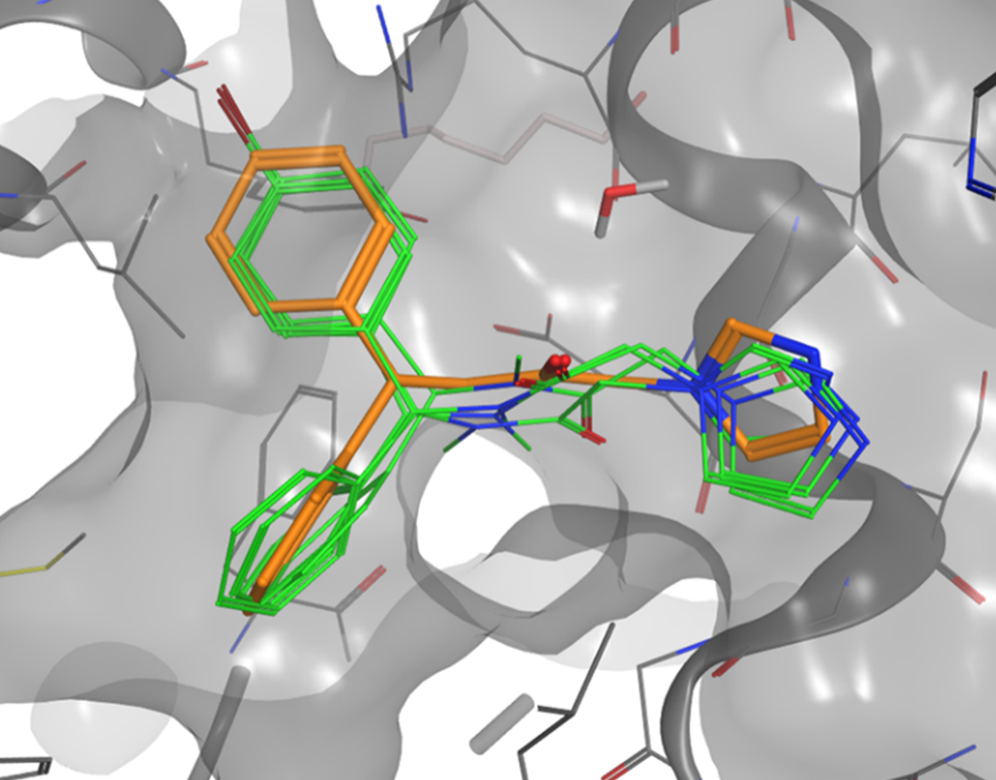
Binding interactions of the four different isomers of **7n** (green) compared with the binding pose of **3** in HO-1.

A molecular docking study was performed as described in the Experimental Section to study the interactions of the reported compounds with HO-1 and HO-2. Initially, we focused our attention on the most potent and selective compound **7n** to explain the selectivity and understand the possible different potencies of the *R*/*S* and *E*/*Z* isomers. The poses of the four different isomers inside the binding pocket of HO-1 are shown in Figure 3. The four different isomers all have a similar pose inside the pocket, where the iron(II) of the heme substrate in HO-1 is correctly coordinated by the nitrogen atom of the imidazole ring of the analyzed molecules in the eastern pocket. Through this coordination binding, iron(II) is protected from oxidation by disrupting an ordered solvent structure involving the critical Asp140 hydrogen-bond network (Tyr58, Tyr114, Arg136, and Asn210) and consequent displacement of water residues needed for catalysis. In all of the docked structures, the unsubstituted phenyl moiety is always located in the principal western region of the binding pocket, whereas the 4-I substituted phenyl ring is always perfectly allocated inside the secondary western region. As shown in Figure 3, the consensus water was retained during the calculation inside the pocket; in fact, it was already shown that this molecule could have a fundamental importance in the enantiomers’ recognitions for ethanolic linkers.^26^ However, different from the ethanolic linker compounds, our new series of the reported molecule cannot interact with the water molecule nor the *R* or the *S* stereoisomer. Actually, compounds **7n** all interact with a pose similar to **3**, where the lone pairs of the carbonyl oxygen in the linker are located in a different plane far away from acting as a H-bond acceptor with the conserved water molecule. Notably, the carbonyl oxygens of compounds *Z*,*R*, and *Z*,*S* are placed in an upward-like fashion as in **3**; differently, the carbonyl oxygens of compounds *E*,*R*, and *E*,*S* are placed in a downward style. In none of the four different isomers, the oxygen is engaging relevant interactions with the protein. The calculated binding energies for the four isomers of **7n** are reported in Table 2. As expected from the binding poses, the calculated energies are very similar for all of the compounds, with slightly higher energies for the isomers where the carbonyl linker’s oxygen is located in an upward specimen similar to **3**. These findings could lead to the conclusion that an enantiomeric resolution could not be an avenue worth purchasing. On the other hand, the *E*/*Z* interconversion, which occurs at room temperature, would move the *E* to *Z* equilibrium toward the most active isomer consumed during the reaction with HO-1.

**Table 2:** Docking Results for **7n** Isomers

compd	Δ*G*_B_ calcd (kcal/mol)	*K*_i_ calcd (μM)
*R*,*E*	–6.51	16.81
*R*,*Z*	–6.63	13.73
*S*,*E*	–6.55	15.71
*S*,*Z*	–6.60	14.44

**Figure 4:**
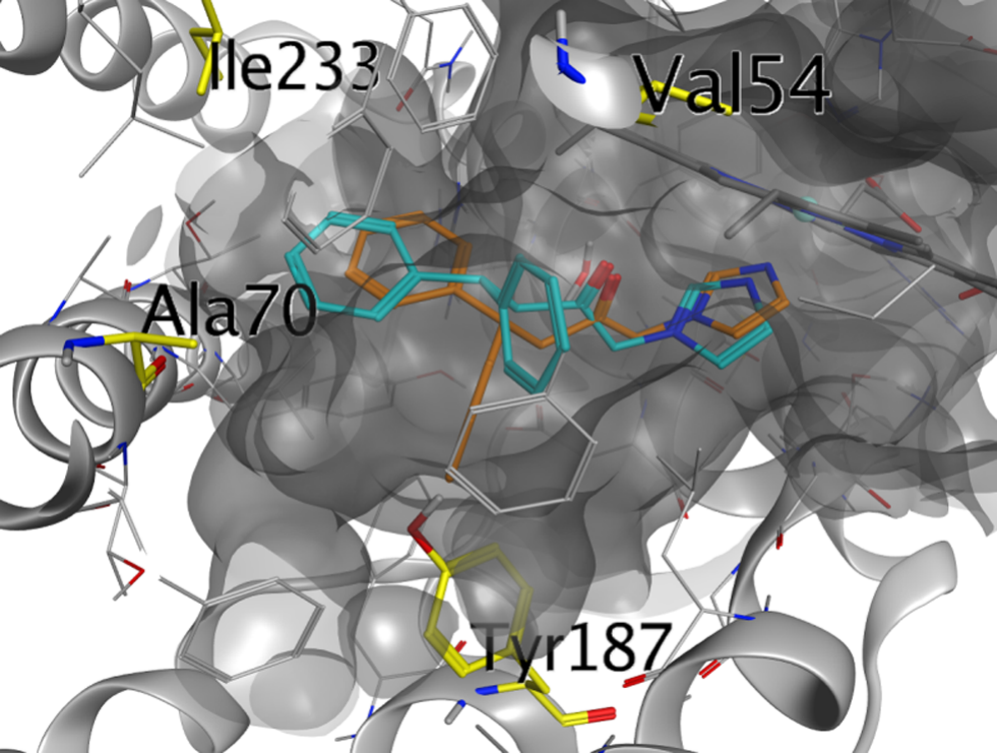
Comparison of compound **3** poses in HO-1 (orange) and HO-2 (light blue). The different residues between HO-2 and HO-1 binding pockets are highlighted in yellow. The highlighted residues belong to HO-2.

Once the most active compound’s binding pose was studied, our attention was focused on the selectivity of the same compound **7n** toward HO-2. The sequence alignment of the HO-2 holoenzyme with that of the HO-1 confirmed that the catalytic cores of these two enzymes are structurally conserved with an root-mean-square deviation (RMSD) of 0.874 Å over the 202 amino acid alignment lengths.^21^ Indeed, among the binding pocket residues, only four differences were seen between the two HO-1 and HO-2: Phe167Tyr187, Val50Ala70, Met34Val54, and Leu213Ile233. The modeling calculation started with the docking of **3** inside HO-2; interestingly, the molecule interacts with the enzyme in a similar but different pose to the one it has in the HO-1 isoform. Remarkably, the predicted most active pose has the two phenyl rings pointing higher in the hydrophobic pocket, with a ring in the secondary western region, as in HO-1, and the second ring pointing in a different area of the pocket, probably due to the presence of Tyr 167 in HO-2 (Figure 4), which partially occupies the binding area.^25^

Closer inspection of the residues involved in compound’s **3** interaction in HO-1 and HO-2 showed that the already reported gate closure by 167 Tyr in HO-2 is most likely the reason for this different pose inside the HO-2 isoform.^25^ Molecules **7i** and **7l**–**n** were then docked in both isoforms to study the binding interactions and the selectivity; only the *R*, *Z* isomers were studied considering the already reported considerations for compound **7n**.

**Figure 5:**
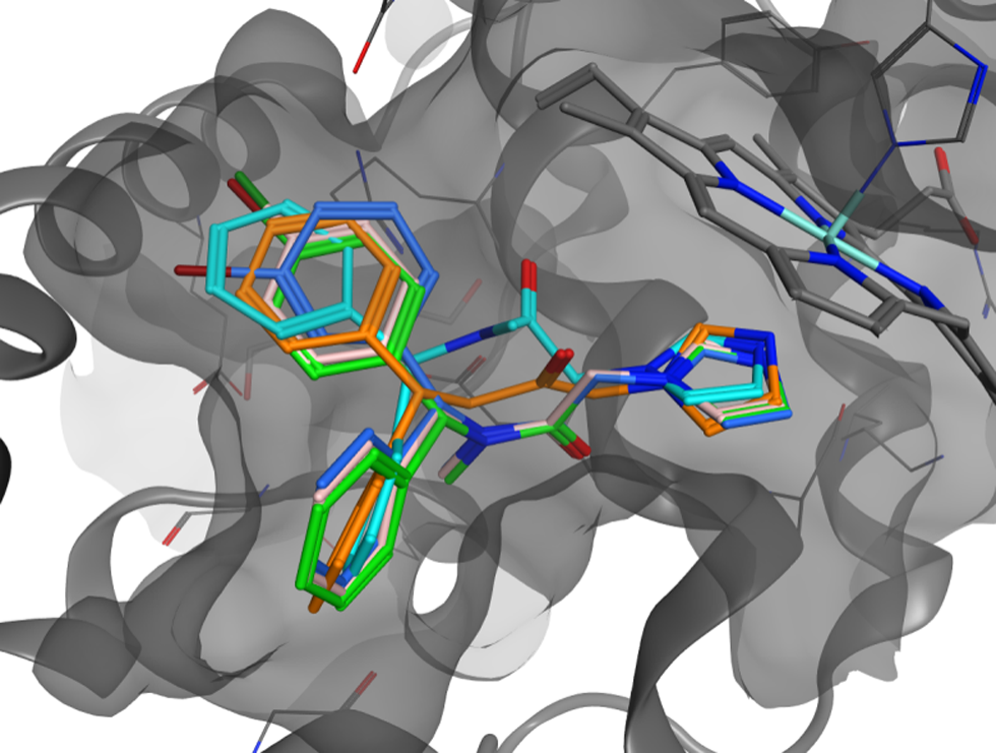
Comparison of **3** binding pose (orange) and **7i** (light blue), **7l** (light pink), **7m** (blue), and **7n** (green) in HO-1.

**Figure 6:**
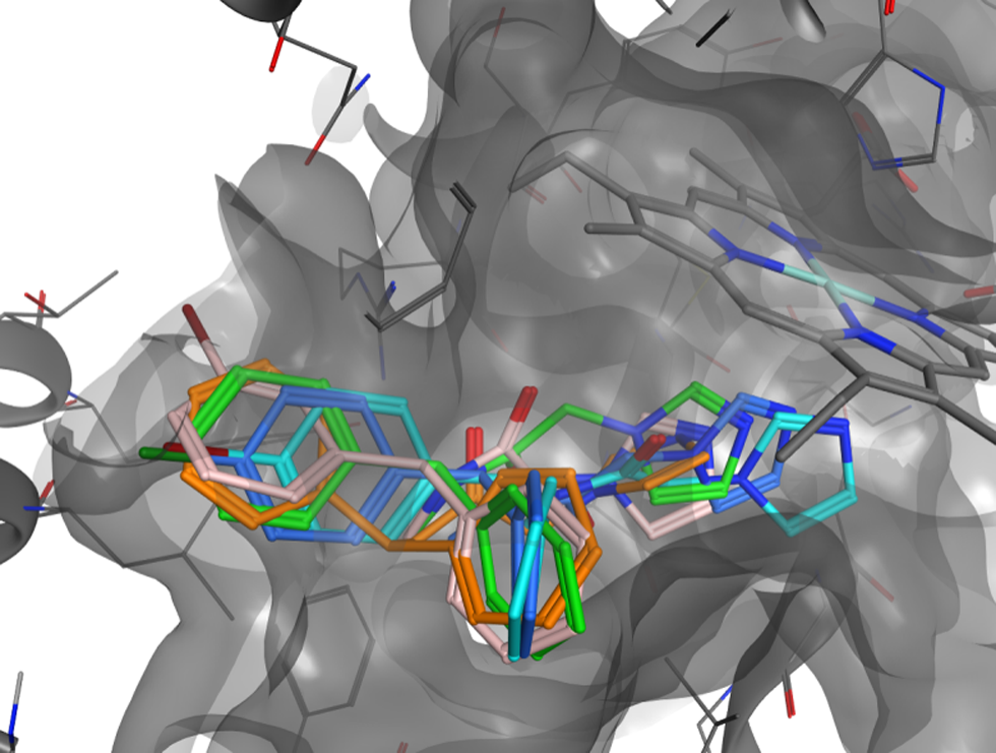
Comparison of **3** binding pose (orange) and **7i** (light blue), **7l** (light pink), **7m** (blue), and **7n** (green) in HO-2.

The docked poses of **7i** and **7l**–**n** inside HO-1 and HO-2 are reported in Figures 5 and 6. All of the molecules have similar poses of **3** inside both the HO-1 and HO-2 isoforms. The calculated binding potencies are in good agreement with the experimental values in the HO-1 and HO-2 inhibition assays (Table 3). These findings suggest that the proposed “double-clamp” binding interaction of **3** can be fine-tuned by the presence of a substituent in a phenyl ring, increasing both the potency and the selectivity of the resulting compounds. The presence of no substituent, as in molecule **7i**, resulted in a similar no-selectivity as in **3** for HO-2. Small halogen atoms in molecules **7l** and **7m** are easily accommodated inside the HO-2 pocket as in HO-1, resulting in an overall no-selectivity over the two isoforms. Unlike the 4-I in the phenyl ring of **7n**, it is too sterically hindered for the pocket and pushes the imidazole ring away from the optimal distance for an effective interaction with the iron, resulting in less potency of the molecule in HO-2.

**Table 3:** Docking Results for **7i** and **7l–n** in HO-1 and HO-2

cmpd	HO-1 Δ*G*_B_ calcd (kcal/mol)	HO-1 *K*_i_ calcd (μM)	HO-2 Δ*G*_B_ calcd (kcal/mol)	HO-2 *K*_i_ calcd (μM)
**7i**	–6.76	11.02	–6.61	14.20
**7l**	–6.52	16.53	–6.46	18.29
**7m**	–6.71	11.99	–6.54	15.98
**7n**	–6.63	13.73	–5.64	73.07

**Figure 7:**
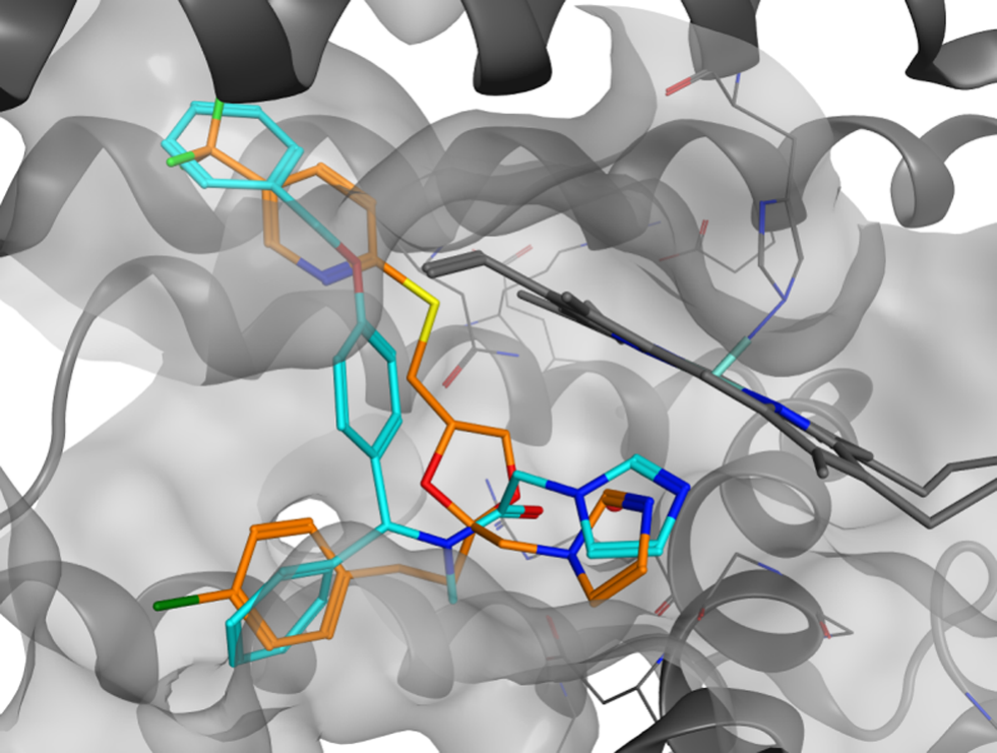
Comparison of compound 1 binding pose (orange) and 7o (light blue) in HO-1.

Finally, the lower selectivity and potency of the most potent compound with the more sterically hindered group, molecule **7o**, could be explained if the compound does not allocate the hindered group in the secondary binding pocket of the western region of HO-1 but in the northeastern pocket (Asn210, Ala31, Ile211, Ala28, and Glu32) in a similar pose of the aromatic region (trifluoromethylpyridine) analogue in compound **1**. Unfortunately, it was concluded that modification in this region would result in neither potency nor selectivity increases and may not be an efficient avenue in developing highly selective HO-1 inhibitors.^23^ Our docking calculation confirmed this; in fact, when the molecule is docked inside the HO-1, it prefers to allocate the hindered group in the northeastern pocket as in compound **1** (Figure 7), with calculated binding energies of −6.63 kcal/mol for HO-1 and −5.78 kcal/mol for HO-2 in agreement with the experimental ones.

This result led to the conclusion that the double-clamp binding pose can increase both potency and selectivity. However, a bigger phenyl substituent would not help target the secondary western pocket because of the northeastern bigger pocket that will preferentially accommodate the sterically more hindered substituent.

### **7l***In Silico* ADMET Assessment

A suitable drug-like profile is an essential element for increasing the chance to advance a preclinical candidate through the drug discovery stages successfully. Therefore, we performed an *in silico* absorption, distribution, metabolism, and excretion-toxicity (ADMET) pharmacokinetics evaluation. The *in silico* assessment has been generated through the evaluation of pharmacokinetic profiles and possible adverse side effects for molecule **7l**. ADMET molecular studies were conducted using SwissADME (http://swissadme.ch)^27^ and pkCSM (http://biosig.unimelb.edu.au/pkcsm/);^28^ the results are reported in Tables S2 and S3. Compound **7l** was predicted as orally available, with high gastrointestinal absorption and soluble in water. The compound does not result as P-glycoprotein and CYP2D6 and CYP2C9 substrates but differently can be a substrate for CYP3A4 (Table S3). Moreover, most of the classical enzyme of the CYP family may be inhibited by our compound, i.e., CYP1A2, CYP2C19, CYP2C9, CYP2D6, and CYP3A4 (Table S2). Interestingly, compound **7l** has no violation to the Lipinski rule of 5; it also has no violation to other drug-likeness rules (Ghose, Egan, Veber, and Muegge).^29−33^ The absorption and distribution calculated parameters have been depicted by the Edan–Egg model in Figure S35 (Brain or IntestinaL EstimateD, BOILED-Egg). The Edan–Egg model highlights that compound **7l** was predicted to passively permeate the blood–brain barrier. pkCSM calculated absorption properties showed a higher than 94% intestinal absorption due to the optimal (> 0.90) Caco-2 cell permeability. The calculated value of steady-state volume of distribution is relatively high for the compounds (Log VDss> 0.45); differently, the compound’s unbound fraction in the plasma is relatively low resulting in a calculated unbound fraction in a human of 0.019. The calculated values of the total clearance indicate that the compound has a good renal elimination (0.662 log mL/min/kg), and it is a substrate of the renal organic cation transporter 2. Finally, no toxicity issues were pointed out by pkCSM; also, the compound resulted as nontoxic in the AMES test, no hepatotoxic, no skin sensitization properties, and generally well tolerated.

### **7l** Preliminary *In Vitro* ADMET Assessment

To further corroborate the *in silico* evaluation, preliminary experimental *in vitro* ADME (i.e., aqueous solubility, bidirectional permeability, metabolic stability, CYP450 inhibition) and toxicology testing (i.e., binding toward hERG potassium channel) were performed on compound **7l** (Table 4).

**Table 4:** *In Vitro* ADMET Profile of **7l**

test type	**7l**	reference cmpd
aqueous solubility^a^	195.8 μM	
bidirectional permeability (Caco-2, pH 6.5/7.4)^b^	*P*_app A-B_ = 44.1 × 10^–6^ cm/s *P*_app B-A_ = 19.4 × 10^–6^ cm/s	*P*_app A-B_ = 26.9 × 10^–6^ cm/s *P*_app B-A_ = 39.7 × 10^–6^ cm/s (propranolol)
microsomal stability^c^ (half-life/intrinsic clearance)	*t*_1/2_ = 118.9 min Clint = 58.3 μL/(min mg) protein	*t*_1/2_ = 88.5 min Clint = 7.8 μL/(min mg) protein (imipramine)
CYP2D6 inhibition^d^	2.5 μM	0.018 μM (quinidine)
CYP3A4 inhibition^d^	0.18 μM	0.046 μM (ketoconazole)
potassium channel hERG binding	5.7^e^	78^f^ (terfenadine)

a Measured in simulated gastric fluid.

b Tested concentration of 10 μM.

c Assessed at a protein concentration of 0.1 mg/mL in the human liver microsome assay.

d CYP isoform inhibition is expressed as an IC_50_ value.

e % inhibition at 1 μM.

f *K*_i_ value in nM.

Since poor solubility and permeability are important factors that might affect both the ADME and the pharmacokinetic properties of a molecule, we initially investigated whether compound **7l** was soluble in mimicking gastric fluid media and able to move across the intestinal epithelial barrier. As predicted by the *in silico* calculations, compound **7l** showed good aqueous solubility at the selected pH, supporting its possible absorption from the stomach (Table 4). In addition, **7l** displayed a suitable apparent permeability coefficient (*P*_app_) in either the A–B (apical to basolateral) or B–A (basolateral to apical) direction, with an efflux ratio <2 (*P*_app BA_/*P*_app AB_ = 0.44). Taken together, these data on solubility and permeability of **7l** are likely indicative of a proper oral bioavailability.

Afterward, *in vitro* metabolic stability of **7l** in human liver microsomes was tested, revealing suitable stability over the period of incubation (up to 1 h) better than imipramine used as a relatively stable reference compound.

As pointed out in the *in silico* ADMET profiling, **7l** and many azole-based compounds are often able to inhibit heme-containing enzymes, including human cytochromes P450 (CYPs), thus potentially interfering and affecting the oxidative metabolism of other drugs.^34,35^ With this in mind, compound **7l** was tested for its effects on human CYP2D6 and CYP3A4, the two CYP isoforms most involved in drug metabolism. The reference compounds quinidine and ketoconazole showed 139-fold and 4-fold higher inhibition of the two CYP isoforms than **7l**.

Finally, to preliminarily investigate potential undesirable cardiovascular side effects of **7l**, binding toward the off-target hERG potassium channel was assessed (**7l** was predicted as a noninhibitor of hERG I but a hERG II inhibitor; Table S3). Notably, **7l** did not display any significant affinity for the selected target (% inhibition at 1 μM = 5.7%), thus suggesting a low risk of cardiovascular liabilities.

### **7l** Isosteric Replacement and SAR Analysis

To enlarge the chemical landscape evaluation and the SAR evaluation of **7l**, a bioisosteric and fragment replacement software tool (Spark v10.4.0, Cresset, New Cambridge House, Hertfordshire, United Kingdom) was adopted to produce a scaffold-hopping analysis and to generate a virtual library of HO-1 ligands. Molecule **7l** was divided into two parts, and a scaffold-hopping analysis was performed for each part (Figure S36). In series 1, the two aromatic rings located in the western region were substituted, and in series 2, the amide linker was substituted. The imidazole nucleus was not replaced due to the important interaction with heme. Two hundred molecules were generated for each substitution, and the best 10 molecules of each series are reported in Tables S4 and S5. All of the molecules were evaluated by the three-dimensional (3D) superposition on an already published HO-1 3D-QSAR model, as reported in the Experimental Section, allowing a fast screening of the data set. As demonstrated by the result of series 1, a different aromatic ring can be used instead of the two phenyls (western region); particularly, the substitution achieved optimal results with an imidazole, tetrazole, and a pyridine ring. Moreover, an aromatic ring of molecule **7l** can also be substituted by nonaromatic substituents (entries 7, 8, and 10; Table S4). The pivot carbon between the two phenyl rings of the western region can also be substituted by a nitrogen without losing activity (entries 4–6 and 9; Table S4). The length of the connecting unit between the imidazole (eastern region) and the aromatic rings of the western region was studied in series 2. As shown in the results of Table S5, the connecting unit can easily contain small alkyl substituents (entries 1, 2, 4 and 5, Table S5); a different length can also be used without losing HO-1 inhibitory activity (entries 3 and 10, Table S5). Overall, thanks to the scaffold-hopping analysis, the SAR of molecule **7l** was further explored and the results indicate that the scaffold replacement generated new structures with the appropriate chemical features for the binding to the HO-1. Some of the selected compounds were more potent than their precursor (3D-QSAR calculated pIC_50_ of **7l** = 6.0), showing again the potential of the models to identify new hits among the library of compounds and would deserve further research investigation to better understand the potential HO-1 inhibitory activity.

### Biological Evaluation: HO-1 Levels in Different Tumoral Cell Lines

**Figure 8:**
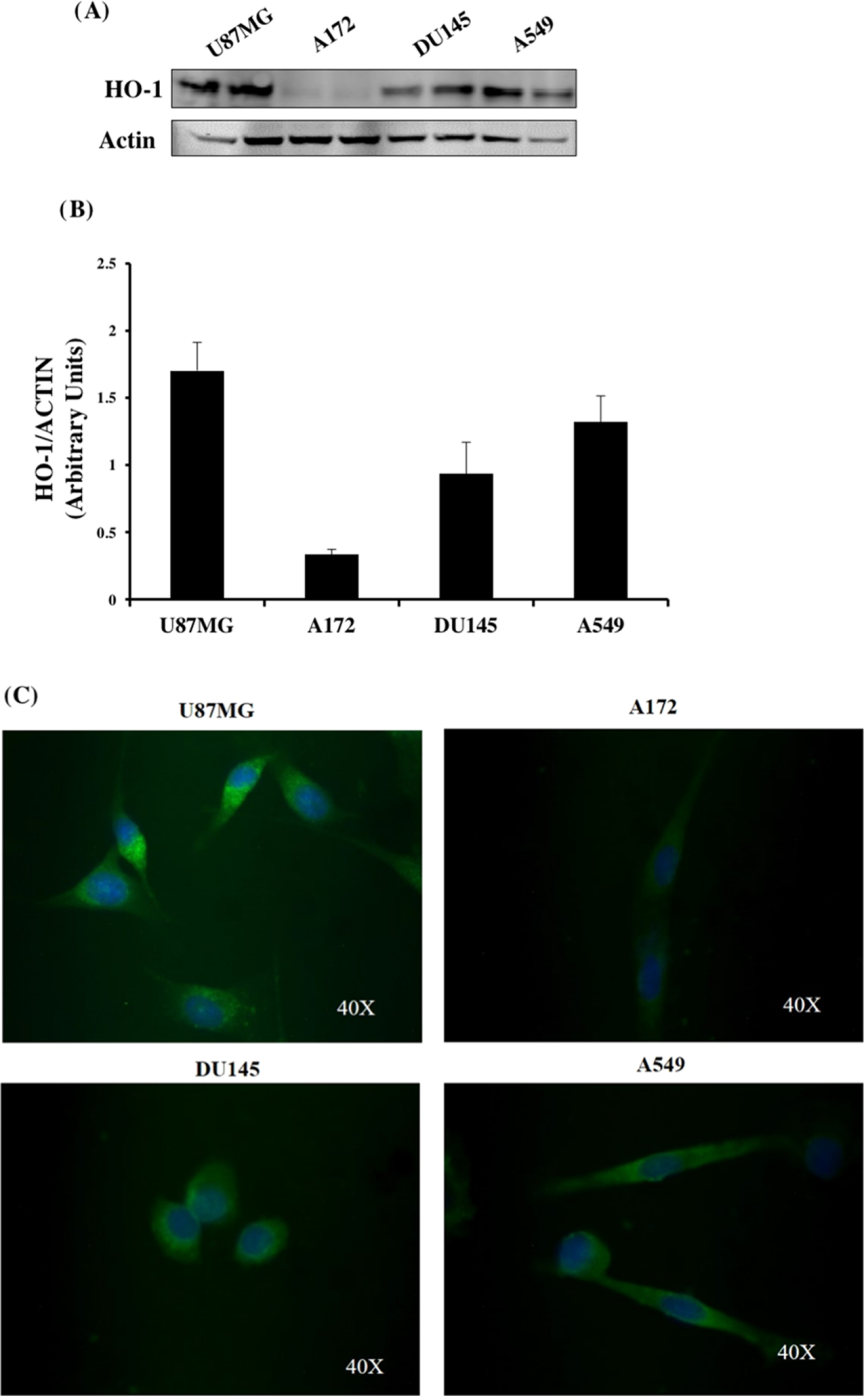
Expression levels of HO-1 in different cancer cell lines. (A) Representative immunoblot of basal HO-1 protein expression detected on cell homogenate of U87Mg, A172, DU145, and A549 cell lines. (B) Bar graphs are representative of results from three independent experiments. Each protein level was expressed as arbitrary units obtained after normalization to actin. (C) Immunolocalization of HO-1 (green fluorescence) in tumor cell lines under basal conditions. Nuclei were stained (blue) with 4′,6-diamidino-2-phenylindole (DAPI). Photomicrographs are representative results of fields taken randomly from slides and scanned by a Zeiss fluorescent microscope.

As suggested by data reported in the literature, HO-1 is differentially expressed in a cellular-specific manner.^36^ To select cancer cell lines more appropriate for studying the effects of our newly identified HO-1 inhibitors, we measured by Western blot analysis the basal HO-1 protein expression in four different cancer cell lines, namely, GBM (U87MG and A172), prostate carcinoma (DU145), and lung adenocarcinoma (A549) (Figure 8A). We choose these cell lines since the associated cancers overexpress HO-1 protein, and their treatment still represents an unmet clinical need.^6,8,10^ As clearly shown in Figure 8, panels A and B, HO-1 levels are significantly higher in U87MG when compared to those of the others. These data were confirmed by immunofluorescence analysis assessing HO-1 immunoreactivity (green fluorescence) in all cell lines under basal conditions (Figure 8C). Microphotographs show HO-1 high signal intensity in U87MG, whereas HO-1 is weakly expressed in A172 mirroring Western blot data. From the immunolocalization panel, we can also highlight that U87MG showed small spots in the perinuclear compartment, allowing us to suppose that in this cell line, HO-1 shows both cytoplasmic and nuclear localization.

### Effect of Compounds on Cancer Cell Viability and HO-1 Protein Expression

**Figure 9:**
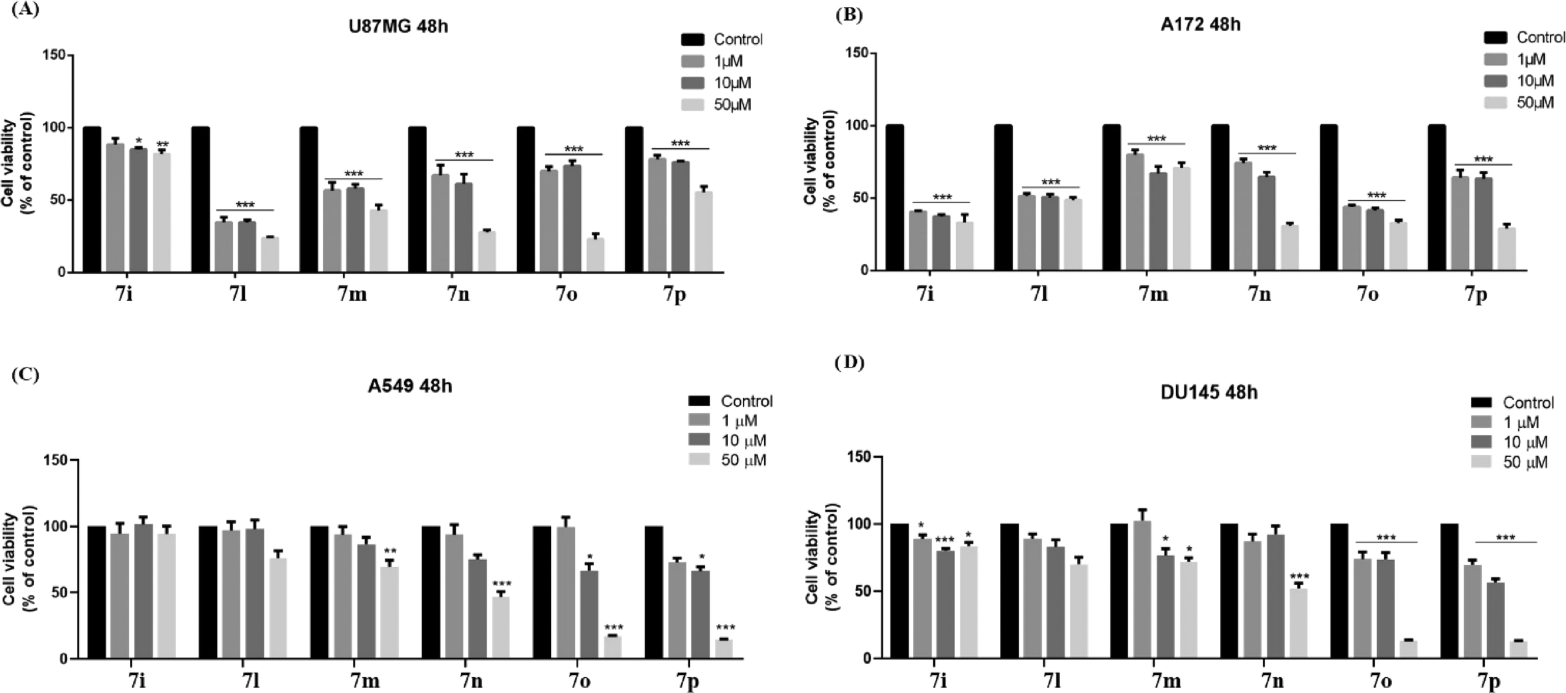
Effect of **7i** and **7l–p** treatments on cell viability of (A) U87MG, (B) A172, (C) A549, and (D) DU145 cell lines, assessed by the MTT assay. Results are representative of at least three independent experiments, and the values are expressed as a percentage of control (% of control). Data represent means ± standard error of the mean (SEM). *p < 0.05, **p < 0.01, or ***p < 0.001 *vs* control.

Following the results obtained from the evaluation of the microsomal enzymatic activity in the presence of the tested compounds, compounds possessing an IC_50_ value ≤8 μM (**7i** and **7l**–**p**) were selected for investigation on cell viability in cancer cell lines. To this extent, we performed an 3-[4,5-dimethylthiazol-2-yl]-2,5-diphenyltetrazolium bromide (MTT) assay at different concentrations (1, 10, and 50 μM) after 48 h of exposure to the selected compounds. Panels A and B of Figure 9 showed that the best results were obtained in both GBM cell lines with reduced cell viability at all concentrations tested. In the U87MG cell line (Figure 9A), all compounds, at 50 μM, showed potent cell viability reduction, except **7i**. Compounds **7m**,**n** determined more than 30% reduction at both concentrations 1 and 10 μM, whereas **7o**,**p** around 20%. By analyzing the cell viability rate induced by the compounds at various concentrations, we can observe that **7l** is the most efficacious inhibitor in the U87MG cell line inducing more than 60% cell viability reduction at all of the tested concentrations. In A172 cell lines (Figure 9B), compounds **7i**-**l** and **7o** showed a remarkable reduction of cell viability at all of the tested concentrations, whereas compounds **7n** and **7p** only at 50 μM and milder activity at 10 μM. Compound **7m** showed weak activity at all of the tested concentrations. Conversely, in the lung adenocarcinoma cell line (A549, Figure 9, panel C), no substances demonstrated efficacy at a 1 μM concentration, whereas **7o** and **7p** showed weak effect at 10 μM. Only compounds **7n**–**p** at a 50 μM concentration showed a significant reduction of cell viability. In prostate carcinoma cell line (DU145, Figure 9, panel D), exclusively **7o** and **7p** had efficacies at 1 μM and 10 μM, whereas **7n**–**p** showed a significant cell viability reduction at 50 μM.

**Figure 10:**
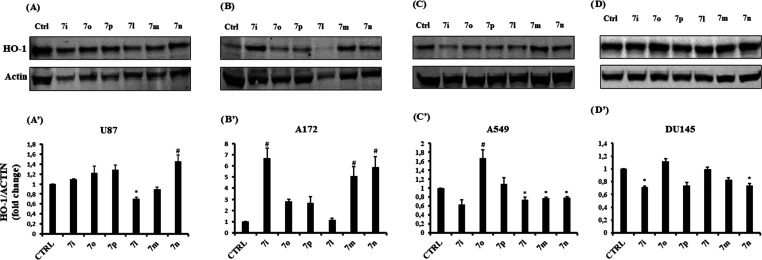
HO-1 expression after **7i** and **7l–p** treatments for 48 h. (A–D) Representative immunoblot of HO-1 protein expression detected on cell homogenate of U87Mg, A172, A549, and DU145 cell lines treated with the selected compounds (10 μM). (A′–D′) Bar graphs are representative of results from three independent experiments. Relative band density was quantified by using LI-COR software. Each protein level was expressed as a fold of change after normalization to actin used as a housekeeping protein. Data represent means ± SEM. *p < 0.05 *vs* control; ^#^*p* < 0.05 vs control.

To investigate the link between cell viability reduction and HO-1 expression, we measured HO-1 levels in cancer cells after 48 h of treatments with 10 μM of each compound, **7i** and **7l**–**p**. Representative immunoblots of the signal detected in U87MG, A172, A549, and DU145 are reported in Figure 10 (panels A–D, respectively). As shown in Figure 10A′,B′, **7l** reduced HO-1 expression only in U87MG. Quite unexpectedly, **7n** enhanced its levels in both U87MG and A172 cell lines, whereas **7i** and **7m** only in A172. In A549 (panel C′), **7l**–**n** weakly downregulated HO-1 levels; conversely, **7o** upregulated its expression. Finally, in DU145, treatment with **7i** and **7n** reduced HO-1 protein expression. By careful analysis of data, compound **7l** was selected further to investigate its molecular mechanism in the U87MG cell line since it was able to significantly reduce cell viability and concomitantly HO-1 expression (Figures 9A and 10A).

### Effect of Compound **7l** on HO-1 Levels and Enzymatic Activity in U87MG Cells

**Figure 11:**
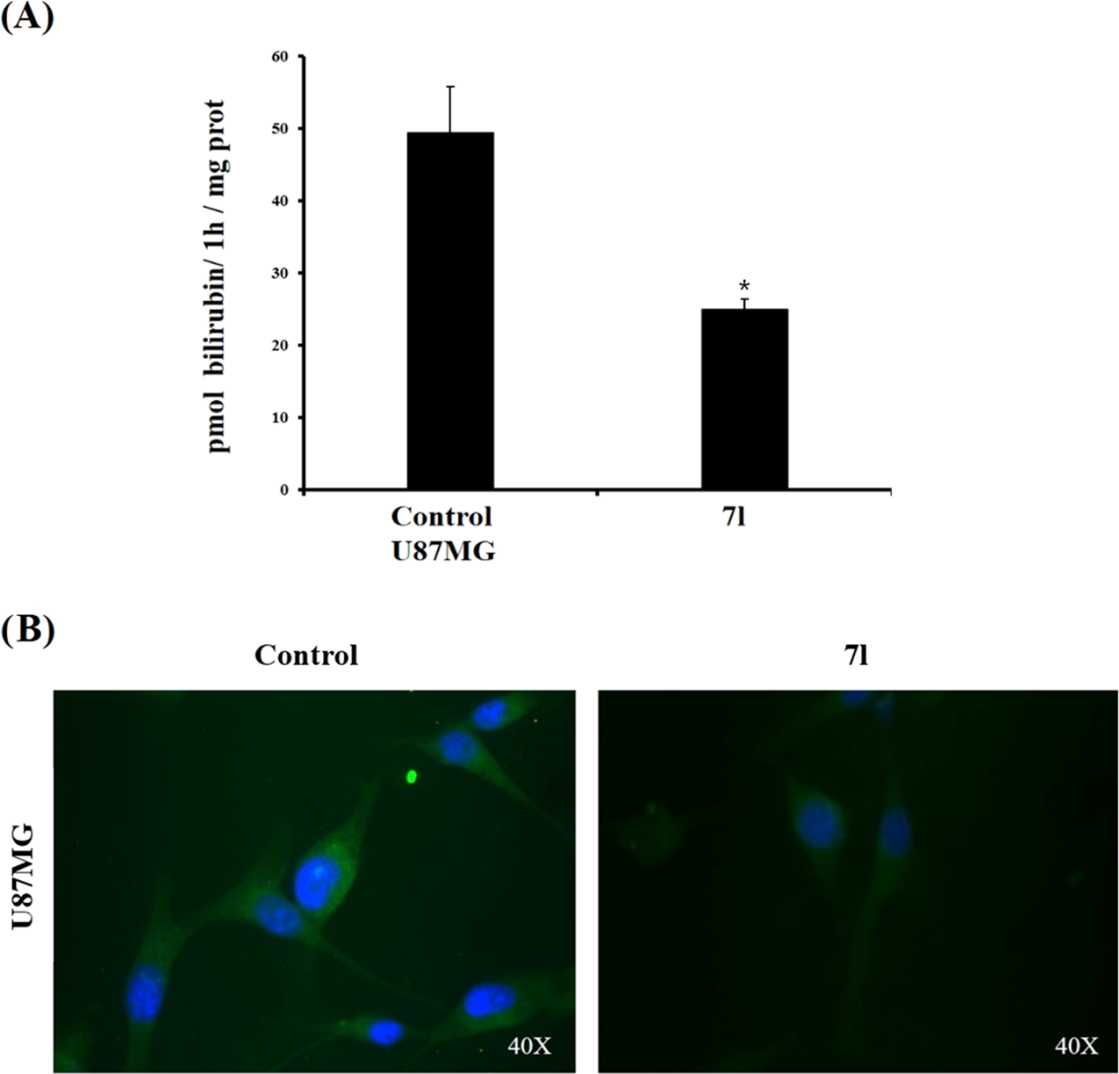
(A) HO enzymatic activity in the U87MG cell line untreated and treated with 10 μM compound **7l**; the results are representative of at least three independent experiments, and values are expressed as pmol of bilirubin/1 h/mg protein. Data represent means ± SEM. *p < 0.01; *vs* control. (B) Immunolocalization of HO-1 (green fluorescence) in the U87MG cell line under basal condition or after 48 h of treatment with **7l** at 10 μM. Nuclei were stained (blue) with DAPI. Photomicrographs are representative results of fields taken randomly from slides and scanned by Zeiss fluorescent microscope.

To evaluate whether HO activity inhibition was maintained in the intact cells, we measured HO enzymatic activity in the U87MG cell line untreated and treated with 10 μM of compound **7l**. The results, described in Figure 11 (Panel A), showed that **7l** reduced HO activity in cell lysates behaving as a HO inhibitor. Therefore, compound **7l** is effective in microsome preparation and in intact cells, suggesting that it can cross the cellular membrane and might have potential *in vivo* application. However, it is known that Mps, which are HO-1 inhibitors structurally related to heme and which consequently act as competitive HO-1 inhibitors, may induce upstream HO-1 expression *in vivo*, giving rise to opposite effects to the expected results.^37^ Consequently, HO-1 induction, together with other side effects of Mps, limits their clinical use. Therefore, we measured HO-1 expression in intact U87MG cells treated and untreated with compound **7l**.

Using immunofluorescence analysis, a technique that allows the detection of antigen in tissues or cells and localizes its distribution in cytoplasmic or perinuclear layers, we showed immunolocalization of HO-1 in the U87MG cell line under basal condition or after 48 h of treatment with **7l** at 10 μM. We demonstrated that the treatment with **7l** reduced the immunoreactivity of HO-1 in U87MG compared to that of control cells (Figure 11, panel B). These data are in agreement with immunoblot analysis (Figure 10A′). Thus, compound **7l**, contrarily to other HO-1 inhibitors such as Mps, does not behave as an HO-1 inducer but can downregulate HO-1 expression and inhibit HO-1 activity.

### Effect of **7l** Treatment on GBM Rate of Cell Invasion and Angiogenesis Process

**Figure 12:**
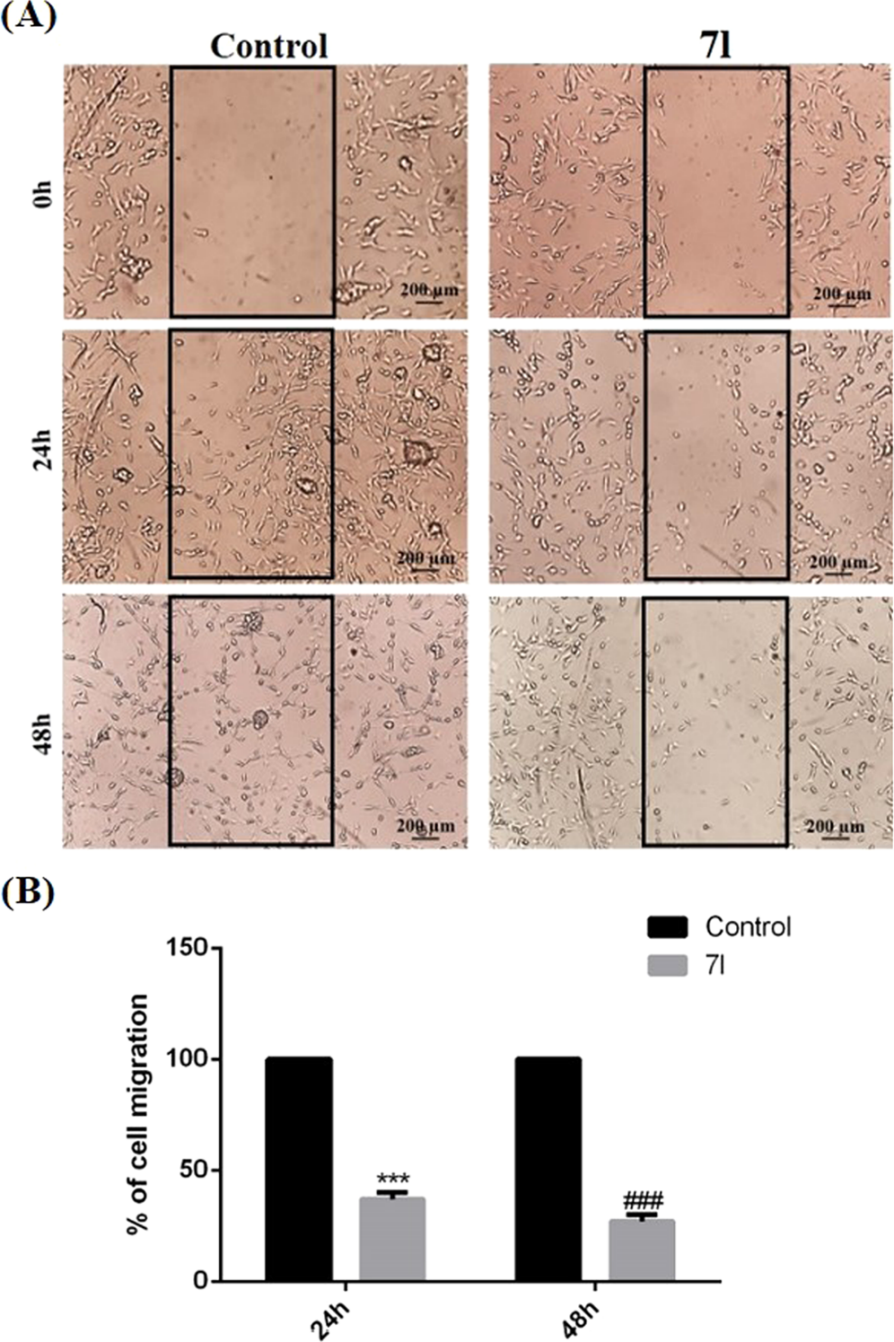
Effect of **7l** on GBM cell migration. (A) Cell monolayer was scraped by a pipette tip and incubated with **7l** compound or vehicle for 48 h. The wounded areas were visualized under a microscope for quantification. Migration was calculated as the average number of cells observed in three random wounded fields/per well in duplicate wells. Scale bar (200 μm). (B) Bar graph shows data expressed as the percentage of control (% of cell migration). Data represent means ± SEM. ***p < 0.0001 *vs* control 24 h; ^###^*p* < 0.0001 *vs* control 48 h.

**Figure 13:**
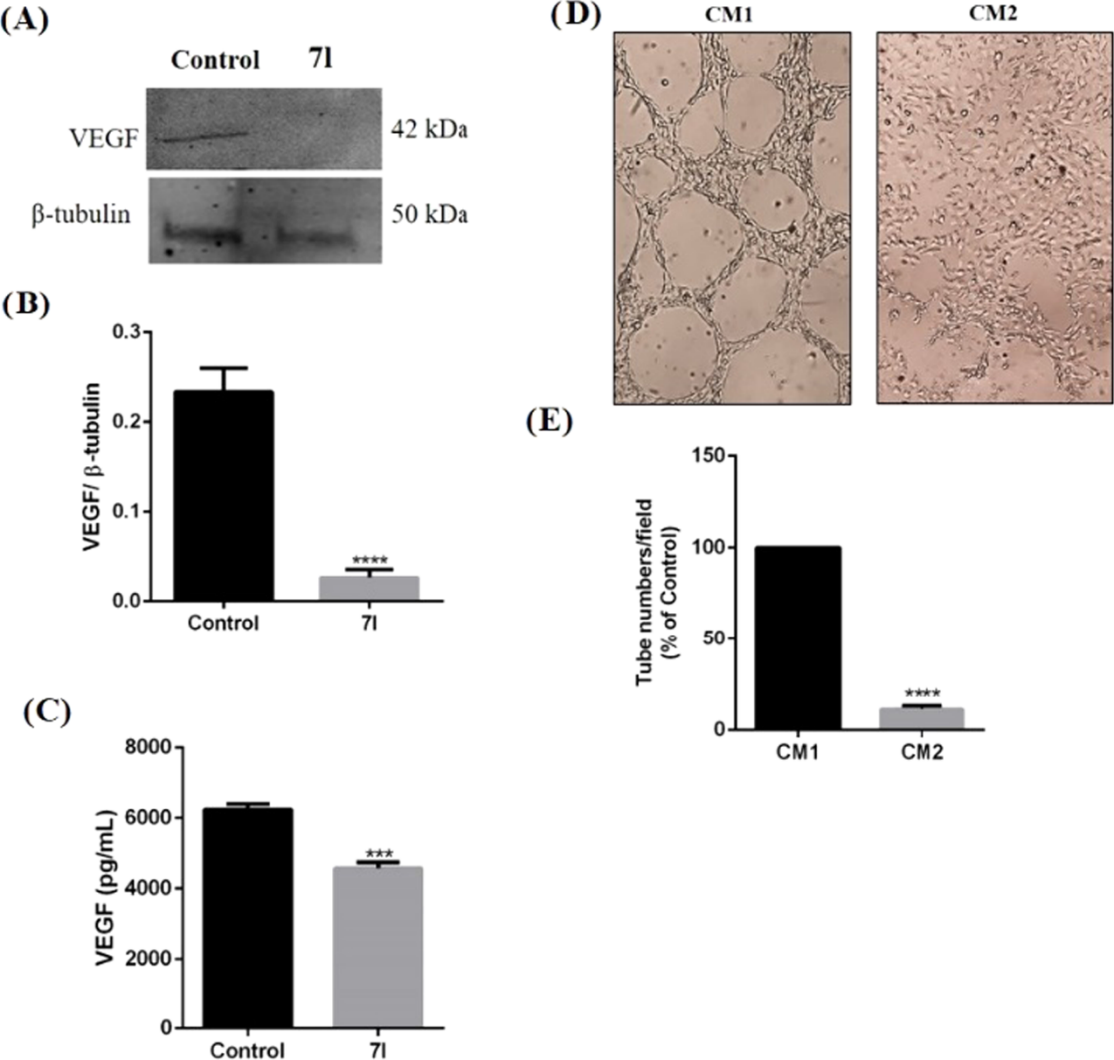
Effect of **7l** on VEGF expression/release in U87MG human GBM cells and new vessel formation. The expression and release of VEGF were evaluated in U87MG cells treated with vehicle or 10 μM of **7l** for 48 h by using Western blot analysis (A, B) and ELISA assay (C). New vessel formation was evaluated by using tube formation assay (D, E). H5V cells were cultured with conditioned medium (CM) derived from U87MG cells treated with vehicle (CM1) or 7l (CM2) for 48 h. In the bar graph, values are expressed as the percentage of control (****p < 0.0001 *vs* control).

It is largely demonstrated that HO-1 is directly linked to neoangiogenesis occurring in tumoral mass with consequent increase of cell invasion rate.^13,38^ In fact, HO-1 can upregulate vascular endothelial growth factor (VEGF) that represents the main trophic factor involved in cancer progression.^6^ Noteworthily, HO-1 gene is considered a potential marker of human glioma neovascularization; therefore, we evaluated the effect of **7l** on cell invasion and neoangiogenesis process.^39,40^ As shown in Figure 12 (panels A and B), we evaluated the effects of **7l** on U87MG cell motility through wound-healing assay. As reported in Figure 12B, cell motility was drastically reduced following 48 h of **7l** treatment compared to control at 24 and 48 h. Furthermore, as reported in Figure 13 (panels A–C), **7l** treatment caused a significant reduction of VEGF intracellular expression and its release in the culture medium of U87MG cells, as demonstrated by Western blot and ELISA assays, respectively. Since VEGF secretion in tumor microenvironment leads to neoangiogenesis, we have further investigated the effect of **7l** in this process. To this end, we have tested the effect of the compound by using endothelial H5V cells that are able to form a network of tube-like structures, mimicking neovessel formation. These cells were cultured with 200 μL of conditioned medium (CM) derived from U87MG cells treated with vehicle (CM1) or **7l** (CM2) for 48 h. As shown in Figure 13 (panels D and E), incubation of H5V cells with CM2, medium derived from U87MG cells cultured with 10 μM of compound **7l**, significantly decreased the number of tube-like structures with respect to control cells.

These data allow us to suggest that **7l** can reduce cell invasivity acting through modulation of HO-1 expression and would encourage further investigation to better understand the modulation of the angiogenesis process by compound **7l**.

## Conclusions

In the present paper, we report the knowledge and structure-based design of new HO-1 inhibitors. Synthetic pathways described above enabled the exploration of the hydrophobic portion and the central linker of the HO-1 pharmacophore employing five different strategies. This exploration clarified the importance of the secondary hydrophobic pocket and the so-called “double-clamp” binding mode. This binding interaction can be fine-tuned by the presence of a substituent in one out of the two phenyl rings, increasing both the potency and the selectivity of resulting compounds. Molecular modeling experiments showed how the newly designed compounds interact with the HO-1 and the molecular properties that lead to potent and selective compounds at the molecular level. Most potent compounds **7i** and **7l**–**p**, tested in a small panel of cancer cell lines, showed interesting antiproliferative profiles, especially in the GBM U87MG cell line. Potent antiproliferative activity and HO-1 expression levels in the U87MG cell line allowed the identification of **7l** as a promising lead compound for further characterization. Compound **7l** was able to potently inhibit enzymatic activity in intact U87MG cells in agreement with immunofluorescence analysis. Also, compound **7l** was showed to significantly reduce VEGF release and new tube formation, suggesting an important role in reducing cell invasivity. Considering that GBM remains still incurable due to its resistance to conventional therapies, this newly reported HO-1 inhibitor **7l** could be considered an interesting starting point to be further explored and optimized as a lead molecule in the management of GBM.

## Experimental Section

### General

Melting points were determined in an IA9200 Electrothermal apparatus equipped with a digital thermometer in capillary glass tubes and are uncorrected. Infrared spectra were recorded on a Perkin Elmer 281 FTIR spectrometer using KBr disks or NaCl plates. Purity of all compounds was ≥ 95% as determined by elemental analyses (C, H, N), which was performed on a Carlo Erba Elemental Analyzer Mod. 1108; results were within ± 0.4% of the theoretical values. ^1^H and ^13^C NMR spectra were recorded on Varian Unity Inova 200 and 500 MHz spectrometers in DMSO-*d*_6_ or CDCl_3_ solution. Chemical shifts are given in ppm values, using tetramethylsilane (TMS) as the internal standard; coupling constants (*J*) are given in Hz. Signal multiplicities are characterized as s (singlet), d (doublet), t (triplet), q (quartet), m (multiplet), br (broad). All reactions were monitored on thin-layer chromatography (TLC) (aluminum sheet coated with silica gel 60 F254, Merck, Kenilworth, NJ) and visualized by UV (λ = 254 and 366 nm) and iodine chamber. Purification of synthesized compounds by flash column chromatography was performed using silica gel 60 (Merck, Kenilworth, NJ) or a Biotage FlashMaster Personal Plus system with prepacked silica gel columns of 25, 50, and 100 g (Biotage SNAP cartridge KP-Sil, Uppsala, Sweden). Microwave-assisted reactions were accomplished with a CEM Discover instrument using closed Pyrex glass tubes with Teflon-coated septa. Where indicated, celite was used as a filter aid. All chemicals and solvents were of reagent grade and were purchased from commercial vendors (Sigma-Aldrich, Fluorochem, TCI chemicals). Compounds **5l**–**m**, **6a**–**d**, **6f**–**h**, **7a**, **7f**, **8o**, **9l**, and **10** have been reported in the literature, and characterization data matched those reported.^41−50^

### General Procedure for the Synthesis of 2-Bromo-*N*-substituted-acetamides (**6a–p**)

In a round-bottom flask, the appropriate starting amine **5a**–**p** (1 equiv) was dissolved in dry CH_3_CN (10 mL). TEA (1.1 equiv) was added, and then α-bromoacetyl bromide (1.1 equiv) was slowly dropped in the solution. The resulting mixture was left stirring at room temperature for 3 h. After this period of time, EtOAc (50 mL) was added, and the organic phase was washed with NaHCO_3_ (2 × 50 mL) and brine (50 mL). The organic phase was dried over anhydrous Na2SO4, filtered, and evaporated. The resulting crude was purified by flash chromatography eluting with a mixture of Cy/EtOAc (7:3). Using this procedure, the following intermediates have been obtained.

#### 2-Bromo-*N*-(4-iodophenyl)-*N*-methylacetamide (**6e**)

Yellow oil (17%): IR (neat) cm^–1^ 3580 (broad), 2983, 1665 (C=O stretch), 1595, 1482, 1431, 1377, 1298, 1221, 1110, 1008, 832; ^1^H NMR (200 MHz, CDCl_3_): mixture of two *E*/*Z* conformers (approximately 50:50) δ 7.79 (d, *J* = 8.6 Hz, 1H, aromatic), 7.50–7.40 (m, 1H, aromatic), 7.30 (m, 1H, aromatic), 7.06 (d, *J* = 8.8 Hz, 1H, aromatic), 3.69 (s, 2H, COCH_2_, conformer *E* (or *Z*)), 3.66 (s, 2H, COCH_2_, conformer *Z* (or *E*)), 3.32 (s, 3H, NCH_3_, conformer *E* (or *Z*)), 3.29 (s, 3H, NCH_3_, conformer *Z* (or *E*)). Anal. calcd for: C_9_H_9_BrINO: C, 30.54; H, 2.56; N, 3.96. Found: C, 30.44; H, 2.55; N, 3.97.

#### *N*-Benzhydryl-2-bromo-*N*-methylacetamide (**6i**)

Colorless oil (90%): IR (neat) cm^–1^ 3060, 3028, 2932, 1652 (C=O stretch.), 1495, 1447, 1396, 1328, 1081, 1031, 977, 751, 731, 699, 600; ^1^H NMR (200 MHz, DMSO-*d*_6_): mixture of two *E*/*Z* conformers (approximately 84:16) δ 7.43–7.29 (m, 6H, aromatic), 7.22–7.13 (m, 4H, aromatic), 6.86 (s, 1H, CHN, conformer *E* (or *Z*)), 6.49 (s, 1H, CHN, conformer *Z* (or *E*)), 4.28 (br s, 2H, COCH_2_), 2.82 (s, 3H, NCH_3_, conformer *E* (or *Z*)), 2.63 (s, 3H, NCH_3_, conformer *Z* (or *E*)). Anal. calcd for: C_16_H_16_BrNO: C, 60.39; H, 5.07; N, 4.40. Found: C, 60.21; H, 5.05; N, 4.41.

#### 2-Bromo-*N*-(cyclopentyl(phenyl)methyl)-*N*-methylacetamide (**6j**)

White semisolid (63%): mp 70–72 °C; IR (neat) cm^–1^ 3447, 2941, 2864, 1634 (C=O stretch.), 1498, 1448, 1402, 1328, 1215, 1134, 1091, 969, 744, 700, 610, 560, 520; ^1^H NMR (200 MHz, DMSO-*d*_6_): mixture of two *E*/*Z* conformers (approximately 88:12) δ 7.52–7.27 (m, 5H, aromatic), 5.36 (d, *J* = 12.0 Hz, 1H, CHN, conformer *E* (or *Z*)), 4.69 (d, *J* = 12.0 Hz, 1H, CHN, conformer *Z* (or *E*)), 4.18, 4.04 (ABq, *J*_AB_ = 28.0 Hz, 2H, CH_2_Br), 2.75 (s, 3H, NCH_3_, conformer *E* (or *Z*)), 2.64 (s, 3H, NCH_3_, conformer *Z* (or *E*)), 1.78–1.48 (m, 7H, cyclopentyl), 1.31–0.95 (m, 2H, cyclopentyl). Anal. calcd for: C_15_H_20_BrNO: C, 58.07; H, 6.50; N, 4.51. Found: C, 58.24; H, 6.52; N, 4.49.

#### 2-Bromo-*N*-(cyclohexyl(phenyl)methyl)-*N*-methylacetamide (**6k**)

Whitish solid (54%): mp 90–93 °C; IR (KBr) cm^–1^ 3448 (broad), 2936, 2851, 1633 (C=O stretch.), 1449, 1402, 1326, 1088, 982, 955, 924, 892, 744, 703, 616, 559; ^1^H NMR (200 MHz, DMSO-*d*_6_): mixture of two *E*/*Z* conformers (approximately 83:17) δ 7.52–7.23 (m, 5H, aromatic), 5.30 (d, *J* = 10.0 Hz, 1H, CHN, conformer *E* (or *Z*)), 4.65–4.34 (m, 2H + 1H, CH_2_Br + CHN, conformer *Z* (or *E*)), 4.16, 4.04 (ABq, *J*_AB_ = 24.0 Hz, 2H, CH_2_Br, conformer *E* (or *Z*)), 2.76 (s, 3H, NCH_3_, conformer *E* (or *Z*)), 2.63 (s, 3H, NCH_3_, conformer *Z* (or *E*)), 2.18 (m, 1H, cyclohexyl), 1.75–0.71 (m, 10H, cyclohexyl). Anal. calcd for: C_16_H_22_BrNO: C, 59.27; H, 6.84; N, 4.32. Found: C, 59.12; H, 6.82; N, 4.33.

#### 2-Bromo-*N*-((4-chlorophenyl)(phenyl)methyl)-*N*-methylacetamide (**6l**)

Yellow oil (34%): IR (neat) cm^–1^ 3030, 2886, 1659 (C=O stretch.), 1644, 1493, 1452, 1394, 1331, 1091, 1015, 978, 848, 742, 703; ^1^H NMR (200 MHz, CDCl_3_): mixture of two *E*/*Z* conformers (approximately 71:29) δ 7.40–7.32 (m, 5H, aromatic), 7.20–7.13 (m, 4H, aromatic), 7.04 (s, 1H, CHN, conformer *E* (or *Z*)), 6.38 (s, 1H, CHN, conformer *Z* (or *E*)) 3.95 (s, 2H, CH_2_Br, conformer *E* (or *Z*)), 3.85 (s, 2H, CH_2_Br, conformer *Z* (or *E*)), 2.89 (s, 3H, NCH_3_, conformer *E* (or *Z*)), 2.75 (s, 3H, NCH_3_, conformer *Z* (or *E*)). Anal. calcd for: C_16_H_15_BrClNO: C, 54.49; H, 4.29; N, 3.97. Found: C, 54.35; H, 4.27; N, 3.98.

#### 2-Bromo-*N*-((3-bromophenyl)(phenyl)methyl)-*N*-methylacetamide (**6m**)

Yellow oil (50%): IR (neat) cm^–1^ 3060, 2932, 1660 (C=O stretch.), 1644, 1568, 1471, 1393, 1325, 1250, 1232, 1174, 1079, 979, 882, 777, 740, 701; ^1^H NMR (200 MHz, CDCl_3_): mixture of two *E*/*Z* conformers (approximately 70:30) δ 7.47–7.28 (m, 5H, aromatic), 7.19 (m, 4H, aromatic), 7.04 (s, 1H, CHN, conformer *E* (or *Z*)), 6.37 (s, 1H, CHN, conformer *Z* (or *E*)), 3.96 (s, 2H, CH_2_Br, conformer *E* (or *Z*)), 3.84 (s, 2H, CH_2_Br, conformer *Z* (or *E*)), 2.90 (s, 3H, NCH_3_, conformer *E* (or *Z*)), 2.76 (s, 3H, NCH_3_, conformer *Z* (or *E*)). Anal. calcd for: C_16_H_15_Br_2_NO: C, 48.39; H, 3.81; N, 3.53. Found: C, 48.51; H, 3.82; N, 3.52.

#### 2-Bromo-*N*-((4-iodophenyl)(phenyl)methyl)-*N*-methylacetamide (**6n**)

Pale yellow oil (78%): IR (neat) cm^–1^ 3452 (broad), 2954, 2361, 1654 (C=O stretch.), 1482, 1399, 1081, 1006, 790; ^1^H NMR (200 MHz, DMSO-*d*_6_): mixture of two *E*/*Z* conformers (approximately 85:15) δ 7.75 (d, *J* = 8.2 Hz, 2H, aromatic), 7.40–7.33 (m, 3H, aromatic), 7.15–7.12 (m, 2H, aromatic), 6.96 (d, *J* = 8.4 Hz, 2H, aromatic), 6.80 (s, 1H, CHN), 4.28 (s, 2H, CH_2_Br), 2.81 (s, 3H, NCH_3_, conformer *E* (or *Z*)), 2.61 (s, 3H, NCH_3_, conformer *Z* (or *E*)). Anal. calcd for: C_16_H_15_BrINO: C, 43.27; H, 3.40; N, 3.15. Found: C, 43.38; H, 3.41; N, 3.14.

#### *N*-((4-(Benzyloxy)phenyl)(phenyl)methyl)-2-bromo-*N*-methylacetamide (**6o**)

White solid (91%): mp 170 °C (dec); IR (neat) cm^–1^ 3292, 3031, 2931, 1734, 1652 (C=O stretch.), 1610, 1509, 1454, 1396, 1244, 1176, 1080, 1025, 845, 736, 698; ^1^H NMR (200 MHz, DMSO-*d*_6_): mixture of two *E*/*Z* conformers (approximately 81:19) δ 7.48–7.28 (m, 8H, aromatic), 7.15–6.99 (m, 6H, aromatic), 6.80 (s, 1H, CHN), 5.10 (s, 2H, CH_2_O), 4.27 (s, 2H, CH_2_Br), 2.81 (s, 3H, NCH_3_, conformer *E* (or *Z*)), 2.62 (s, 3H, NCH_3_, conformer *Z* (or *E*)). Anal. calcd for: C_23_H_22_BrNO_2_: C, 65.10; H, 5.23; N, 3.30. Found: C, 64.99; H, 5.21; N, 3.31.

#### 2-Bromo-*N*-((4-((4-bromobenzyl)oxy)phenyl)(phenyl)methyl)-*N*-methylacetamide (**6p**)

Colorless oil (78%): IR (neat) cm^–1^ 3032, 2365, 1654 (C=O stretch.), 1609, 1508, 1457, 1396, 1244, 1175, 1080, 1025, 736, 698, 666; ^1^H NMR (200 MHz, DMSO-*d*_6_): mixture of two *E*/*Z* conformers (approximately 83:17) δ 7.48–7.32 (m, 7H, aromatic), 7.20–7.00 (m, 6H, aromatic), 6.80 (s, 1H, CHN), 5.10 (s, 2H, CH_2_O), 4.56 (s, 2H, CH_2_Br, conformer *Z* (or *E*)), 4.27 (s, 2H, CH_2_Br, conformer *E* (or *Z*)), 2.81 (s, 3H, NCH_3_, conformer *E* (or *Z*)), 2.62 (s, 3H, NCH_3_, conformer *Z* (or *E*)). Anal. calcd for: C_23_H_21_Br_2_NO_2_: C, 54.90; H, 4.21; N, 2.78. Found: C, 54.77; H, 4.20; N, 2.79.

### General Procedure for the Synthesis of Final Compounds **7c–d**, **7g–i**, and **7o** (Method A)

In a round-bottom flask, K_2_CO_3_ (3 equiv) was suspended in dry DMF (6 mL). Imidazole (3 equiv) was added to the suspension under stirring. The appropriate α-bromo-acetamide intermediate **6** (1 equiv) was dissolved in dry DMF (6 mL) and dropped to the suspension, which was left stirring at room temperature for 2 h. The resulting mixture was concentrated under vacuum, and then EtOAc (100 mL) was added. The organic phase was washed with 1 N NaOH (2 × 100 mL) and brine (1 × 100 mL). The organic phase was dried over anhydrous Na_2_SO_4_, filtered, and evaporated. The resulting crude was purified by crystallization in EtOAc or flash chromatography using a mixture of CH_2_Cl_2_/MeOH (9.5:0.5) as eluent. Using this procedure, the following final compounds have been synthesized.

#### *N*-(3-Bromophenyl)-2-(1*H*-imidazol-1-yl)acetamide (**7c**)

Purified by flash chromatography (9.5 CH_2_Cl_2_/0.5 MeOH). Brownish solid (16%): mp 158–162 °C; IR (KBr) cm^–1^ 3245, 2915, 2849, 2783, 1698 (C=O stretch.), 1623, 1594, 1543, 1509, 1472, 1420, 1307, 1269, 1198, 1108, 1080, 1033, 870, 830, 783, 741, 684, 656; ^1^H NMR (500 MHz, DMSO-*d*_6_): δ 10.50 (s, 1H, NH), 7.91 (s, 1H, imidazole), 7.70 (s, 1H, imidazole), 7.48 (d, J = 5.0 Hz, 1H, aromatic), 7.31–7.25 (m, 2H, aromatic), 7.18 (s, 1H, aromatic), 6.93 (s, 1H, imidazole), 4.92 (s, 2H, COCH_2_); ^13^C NMR (125 MHz, DMSO-*d*_6_): δ 166.17, 140.20, 131.02, 127.58, 126.38, 121.72, 121.67, 121.00, 118.08, 49.32. Anal. calcd for: C_11_H_10_BrN_3_O: C, 47.16; H, 3.60; N, 15.00. Found: C, 47.23; H, 3.60; N, 14.97.

#### 2-(1*H*-Imidazol-1-yl)-*N*-(4-iodophenyl)acetamide (**7d**)

Purified by flash chromatography (9.5 CH_2_Cl_2_/0.5 MeOH). Beige crystals (66%): mp 222–225 °C; IR (KBr) cm^–1^ 3258, 3181, 2954 (broad), 1706 (C=O stretch.), 1617, 1585, 1549, 1508, 1484, 1391, 1302, 1250, 1198, 1108, 1101, 1081, 916, 825; ^1^H NMR (500 MHz, DMSO-*d*_6_): δ 10.45 (s, 1H, NH), 7.66 (d, *J* = 10.0 Hz, 2H, aromatic), 7.64 (s, 1H, imidazole), 7.43 (d, *J* = 10.0 Hz, 2H, aromatic), 7.16 (s, 1H, imidazole), 6.90 (s, 1H, imidazole), 4.90 (s, 2H, CH_2_CO); ^13^C NMR (125 MHz, DMSO-*d*_6_): δ 165.95, 138.47, 138.32, 137.51, 127.89, 121.28, 120.74, 87.12, 49.17. Anal. calcd for: C_11_H_10_IN_3_O: C, 40.39; H, 3.08; N, 12.85. Found: C, 40.43; H, 3.09; N, 12.82.

#### *N*-Benzyl-2-(1*H*-imidazol-1-yl)-*N*-methylacetamide (**7g**)

Purified by flash chromatography (9.5 CH_2_Cl_2_/0.5 MeOH). Whitish solid (27%): mp 106–111 °C; IR (KBr) cm^–1^ 3313, 3132, 3113, 3025, 2982, 2916, 1674 (C=O stretch.), 1603, 1485, 1453, 1406, 1357, 1341, 1307, 1291, 1235, 1204, 1121, 1071, 1038, 1004, 966, 948, 908, 824, 757, 735, 697, 665, 640, 593; ^1^H NMR (500 MHz, DMSO-*d*_6_): mixture of two *E*/*Z* conformers (approximately 70:30) δ 7.55 (s, 1H, imidazole), 7.42–7.25 (m, *5*H, aromatic), 7.08 (s, 1H, imidazole, conformer *E* (or *Z*)), 7.06 (s, 1H, imidazole, conformer *Z* (or *E*)), 6.87 (s, 1H, imidazole, conformer *E* (or *Z*)), 6.86 (s, 1H, imidazole, conformer *Z* (or *E*)), 5.09 (s, 2H, ArCH_2_N, conformer *E* (or *Z*)), 5.06 (s, 2H, ArCH_2_N, conformer *Z* (or *E*)), 4.63 (s, 2H, COCH_2_, conformer *E* (or *Z*)), 4.53 (s, 2H, COCH_2_, conformer *Z* (or *E*)), 2.97 (s, 3H, NCH_3_, conformer *E* (or *Z*)), 2.80 (s, 3H, NCH_3_, conformer *Z* (or *E*)); ^13^C NMR (125 MHz, DMSO-*d*_6_): δ 167.29, 167.06, 138.33, 137.29, 136.77, 128.80, 128.49, 127.60, 127.52, 127.16, 127.09, 120.96, 120.91, 51.69, 50.41, 47.30, 47.16, 33.83, 33.50. Anal. calcd for: C_13_H_15_N_3_O: C, 68.10; H, 6.59; N, 18.33. Found: C, 68.24; H, 6.60; N, 18.27.

#### *N*,*N*-Dibenzyl-2-(1*H*-imidazol-1-yl)acetamide (**7h**)

Purified by flash chromatography (9.5 CH_2_Cl_2_/0.5 MeOH). White solid (42%): mp 99–102 °C; IR (KBr) cm^–1^ 3026, 2943, 1641 (C=O stretch.), 1513, 1495, 1451, 1438, 1422, 1346, 1315, 1291, 1261, 1218, 1199, 1105, 1078, 1038, 909, 819, 775, 753, 693, 666, 625, 579, 506; ^1^H NMR (500 MHz, DMSO-*d*_6_): δ 7.57 (s, 1H, imidazole), 7.40 (t, *J* = 7.0 Hz, 2H, aromatic), 7.32 (t, *J* = 7.5 Hz, 3H, aromatic), 7.26 (t, *J* = 7.0 Hz, 3H, aromatic), 7.21 (d, *J* = 7.5 Hz, 2H, aromatic), 7.07 (s, 1H, imidazole), 6.87 (s, 1H, imidazole), 5.10 (s, 2H, COCH_2_), 4.58 (s, 2H, ArCH_2_N), 4.48 (s, 2H, ArCH_2_N); ^13^C NMR (125 MHz, DMSO-*d*_6_): δ 167.74, 138.57, 137.10, 136.56, 129.01, 128.64, 127.88, 127.76, 127.38, 127.20, 121.12, 49.60, 48.56, 47.39. Anal. calcd for: C_19_H_19_N_3_O: C, 74.73; H, 6.27; N, 13.76. Found: C, 74.98; H, 6.29; N, 13.75.

#### *N*-Benzhydryl-2-(1*H*-imidazol-1-yl)-*N*-methylacetamide (**7i**)

Crystallized from EtOAc. White crystals (11%): mp 121–126 °C; IR (KBr) cm^–1^ 3445 (broad), 3030, 2937, 1659 (C=O stretch.), 1643, 1508, 1498, 1477, 1451, 1400, 1233, 1109, 1073, 1031, 969, 901, 818, 751, 725, 699; ^1^H NMR (500 MHz, DMSO-*d*_6_): mixture of two *E*/*Z* conformers (approximately 85:15) δ 7.56 (s, 1H, imidazole), 7.41–7.32 (m, 6H, aromatic), 7.24–7.17 (m, 4H, aromatic), 7.09 (s, 1H, imidazole), 6.89 (s, 1H, imidazole), 6.87 (s, 1H, CHN, conformer *E* (or *Z*)), 6.53 (s, 1H, CHN, conformer *Z* (or *E*)), 5.17 (s, 2H, COCH_2_, conformer *E* (or *Z*)), 5.13 (s, 2H, COCH_2_, conformer *Z* (or *E*)), 2.83 (s, 3H, NCH_3_, conformer *E* (or *Z*)), 2.64 (s, 3H, NCH_3_, conformer *Z* (or *E*)); ^13^C NMR (125 MHz, DMSO-*d*_6_): δ 167.77, 138.87, 138.34, 128.53, 128.50, 127.79, 127.61, 127.46, 120.98, 60.34, 47.53, 30.97. Anal. calcd for: C_19_H_19_N_3_O: C, 74.73; H, 6.27; N, 13.76. Found: C, 74.81; H, 6.28; N, 13.73.

#### *N*-((4-(Benzyloxy)phenyl)(phenyl)methyl)-2-(1*H*-imidazol-1-yl)-*N*-methylacetamide (**7o**)

Purified by flash chromatography (9.5 CH_2_Cl_2_/0.5 MeOH). White solid (14%): mp 119–121 °C; IR (KBr) cm^–1^ 3447 (broad), 3111, 3034, 2977, 1654 (C=O stretch.), 1638, 1513, 1476, 1456, 1401, 1301, 1254, 1235, 1178, 1113, 1074, 1042, 1028, 844, 732, 705; ^1^H NMR (500 MHz, DMSO-*d*_6_): mixture of two *E*/*Z* conformers (approximately 80:20) δ 7.58 (s, 1H, imidazole), 7.46 (d, *J* = 5.0 Hz, 2H, aromatic), 7.40 (q, *J* = 7.0 Hz, 3H, aromatic), 7.34 (q, *J* = 7.0 Hz, 2H, aromatic), 7.24–7.23 (m, 1H, aromatic), 7.17 (d, *J* = 10.0 Hz, 2H, aromatic), 7.09 (d, *J* = 5.0 Hz, 2H, aromatic), 7.03 (d, *J* = 10.0 Hz, 2H, aromatic), 6.88 (s, 1H, imidazole), 6.82 (s, 1H, imidazole), 5.19–5.11 (m, 2H + 1H, COCH_2_ + CHN), 3.62 (s, 2H, CH_2_O) 2.82 (s, 3H, NCH_3_, conformer *E* (or *Z*)), 2.63 (s, 3H, NCH_3_, conformer *Z* (or *E*)); ^13^C NMR (125 MHz, DMSO-*d*_6_): δ 167.87, 157.86, 139.32, 138.58, 137.19, 131.10, 130.19, 128.93, 128.72, 128.37, 128.15, 127.96, 127.69, 127.58, 121.31, 114.97, 69.46, 60.07, 47.79, 31.03. Anal. calcd for: C_26_H_25_N_3_O_2_: C, 75.89; H, 6.12; N, 10.21. Found: C, 76.12; H, 6.12; N, 10.18.

### General Procedure for the Synthesis of Final Compounds **7b**, **7e**, **7j**–**n**, and **7p** (Method B)

In a two-necked round-bottom flask, imidazole (3 equiv) was dissolved in dry THF (10 mL) under a N_2_ flow. Subsequently, NaH (oil dispersion 80%) (5 equiv) was added, and the resulting suspension was left stirring for 15 minutes. The appropriate α-bromo-acetamide derivative **6** (1 equiv) was dissolved in anhydrous THF (10 mL) in an inert atmosphere and subsequently dropped on the suspension of NaH and imidazole via syringe. The suspension was left under stirring at room temperature for 16 h. Then, deionized water was added, and the resulting mixture was extracted three times with EtOAc (3×100 mL). The combined organic phases were washed with 150 mL of an aqueous solution of 1 M NaOH, dried on Na_2_SO_4_, filtered, and concentrated under vacuum. The resulting oil was purified by flash chromatography using a mixture of CH_2_Cl_2_/MeOH (9.5:0.5) as eluent. The pure oils were then triturated with cold Et_2_O affording final compounds as white solids, except for compounds **7e** and **7j–l**.

#### 2-(1*H*-Imidazol-1-yl)-*N*-methyl-*N*-phenylacetamide (**7b**)

White solid (31%): mp 117–119 °C; IR (KBr) cm^–1^ 3446 (broad), 3112, 2938, 1670 (C=O stretch.), 1595, 1513, 1495, 1418, 1393, 1329, 1295, 1236, 1125, 1080, 907, 826, 799, 774, 752, 697, 664, 562; ^1^H NMR (500 MHz, DMSO-*d*_6_): δ 7.50–7.45 (m, 6H, aromatic + imidazole), 7.01 (s, 1H, imidazole), 6.81 (s, 1H, imidazole), 4.58 (s, 2H, COCH_2_), 3.18 (s, 3H, NCH_3_).; ^13^C NMR (125 MHz, DMSO-*d*_6_): δ 166.36, 142.17, 138.32, 129.96, 128.25, 127.59, 120.83, 47.86, 37.31. Anal. calcd for: C_12_H_13_N_3_O: C, 66.96; H, 6.09; N, 19.52. Found: C, 67.03; H, 6.10; N, 19.48.

#### 2-(1*H*-Imidazol-1-yl)-*N*-(4-iodophenyl)-*N*-methylacetamide (**7e**)

Light yellowish oil (47%): IR (neat) cm^–1^ 3375 (broad), 3113, 2919, 1671 (C=O stretch.), 1595, 1508, 1497, 1419, 1394, 1327, 1292, 1235, 1124, 1079, 914, 800, 774, 739, 702, 663^1^H NMR (500 MHz, DMSO-*d*_6_): mixture of two *E*/*Z* conformers (approximately 73:27) δ 7.86–7.84 (m, 1H, aromatic), 7.53–7.42 (m, 2H + 1H, aromatic + imidazole), 7.28–7.26 (m, 1H, aromatic), 7.01 (s, 1H, imidazole), 6.82 (s, 1H, imidazole), 4.63 (s, 2H, COCH_2_, conformer *E* (or *Z*)), 4.59 (s, 2H, COCH_2_, conformer *Z* (or *E*)), 3.20 (s, 3H, NCH_3_, conformer *E* (or *Z*)), 3.18 (s, 3H, NCH_3_, conformer *Z* (or *E*)); ^13^C NMR (125 MHz, DMSO-*d*_6_): δ 166.27, 142.15, 138.64, 138.21, 129.85, 128.11, 127.54, 120.72, 109.81, 47.77, 37.21, 37.02. Anal. calcd for: C_12_H_12_IN_3_O: C, 42.25; H, 3.55; N, 12.32. Found: C, 42.39; H, 3.56; N, 12.28.

#### *N*-(Cyclopentyl(phenyl)methyl)-2-(1*H*-imidazol-1-yl)-*N*-methylacetamide (**7j**)

White solid (84%): mp 134–136 °C; IR (KBr) cm^–1^ 3447 (broad), 2952, 2865, 1654 (C=O stretch.), 1638, 1508, 1456, 1403, 1313, 1234, 1109, 1073, 908, 817, 760, 742, 702, 665, 567; ^1^H NMR (500 MHz, DMSO-*d*_6_): mixture of two *E*/*Z* conformers (approximately 87:13) δ 7.60 (s, 1H, imidazole, conformer *E* (or *Z*)), 7.52 (s, 1H, imidazole, conformer *Z* (or *E*)), 7.48–7.25 (m, 5H, aromatic), 7.08 (s, 1H, imidazole, conformer *E* (or *Z*)), 7.03 (s, 1H, imidazole, conformer *Z* (or *E*)), 6.85 (s, 1H, imidazole), 5.33–5.16 (m, 2H + 1H, COCH_2_ + CHN, conformer *E* (or *Z*)), 5.00, 4.92 (ABq, *J*_AB_ =15.0 Hz, 2H, COCH_2_, conformer *Z* (or *E*)), 4.64 (d, *J* = 11.0 Hz, 1H, CHN, conformer *Z* (or *E*)), 2.75 (s, 3H, NCH_3_, conformer *E* (or *Z*)), 2.63 (s, 3H, NCH_3_, conformer *Z* (or *E*)), 1.75–1.69 (m, 1H, cyclopentyl), 1.63–1.49 (m, 5H, cyclopentyl), 1.25–1.18 (m, 1H, cyclopentyl), 1.00–0.93 (m, 1H, cyclopentyl); ^13^C NMR (125 MHz, DMSO-*d*_6_): δ 167.36, 166.72, 139.67, 139.34, 138.50, 128.75, 128.62, 128.45, 127.94, 127.70, 127.63, 121.19, 64.14, 60.85, 47.80, 47.67, 38.24, 30.59, 30.43, 29.96, 29.73, 28.61, 28.01, 25.59, 25.42, 25.29, 25.23. Anal. calcd for: C_18_H_23_N_3_O: C, 72.70; H, 7.80; N, 14.13. Found: C, 72.88; H, 7.82; N, 14.11.

#### *N*-(Cyclohexyl(phenyl)methyl)-2-(1*H*-imidazol-1-yl)-*N*-methylacetamide (**7k**)

White solid (70%): mp 130–133 °C; IR (KBr) cm^–1^ 3447 (broad), 2923, 2852, 1655 (C=O stretch.), 1645, 1508, 1448, 1405, 1317, 1294, 1234, 1138, 1107, 1077, 812, 742, 702, 662; ^1^H NMR (500 MHz, DMSO-*d*_6_): mixture of two *E*/*Z* conformers (approximately 83:17) δ 7.60 (s, 1H, imidazole, conformer *E* (or *Z*)), 7.50 (s, 1H, imidazole, conformer *Z* (or *E*)), 7.46–7.25 (m, 5H, aromatic), 7.07 (s, 1H, imidazole, conformer *E* (or *Z*)), 7.03 (s, 1H, imidazole, conformer *Z* (or *E*)), 6.86 (s, 1H, imidazole, conformer *E* (or *Z*)), 6.84 (s, 1H, imidazole, conformer *Z* (or *E*)), 5.28–5.15 (m, 2H + 1H, COCH_2_ + CHN, conformer *E* (or *Z*)), 5.01, 4.88 (ABq, *J*_AB_ = 15.0 Hz, 2H, COCH_2_, conformer *Z* (or *E*)), 4.52 (d, *J* = 10.0 Hz, 1H, CHN, conformer *Z* (or *E*)), 2.75 (s, 3H, NCH_3_, conformer *E* (or *Z*)), 2.61 (s, 3H, NCH_3_, conformer *Z* (or *E*)), 2.20–2.10 (m, 1H, cyclohexyl), 1.78–1.55 (m, 4H, cyclohexyl), 1.39–1.06 (m, 4H, cyclohexyl), 0.93–0.86 (m, 1H, cyclohexyl), 0.78–0.70 (m, 1H, cyclohexyl); ^13^C NMR (125 MHz, DMSO-*d*_6_): δ 167.54, 167.19, 138.63, 138.13, 128.99, 128.89, 128.16, 127.86, 127.68, 121.38, 64.87, 61.49, 48.01, 47.80, 36.85, 35.44, 30.61, 30.32, 29.41, 29.28, 28.70, 27.94, 26.23, 26.11, 25.84, 25.71, 25.57, 25.49. Anal. calcd for: C_19_H_25_N_3_O: C, 73.28; H, 8.09; N, 13.49. Found: C, 73.41; H, 8.10; N, 13.45.

#### *N*-((4-Chlorophenyl)(phenyl)methyl)-2-(1*H*-imidazol-1-yl)-*N*-methylacetamide (**7l**)

Colorless solid (84%): mp 60–61 °C: IR (neat) cm^–1^ 3384 (broad), 2932, 1660 (C=O stretch.), 1510, 1491, 1404, 1299, 1108, 1078, 1014, 828, 740, 704; ^1^H NMR (500 MHz, DMSO-*d*_6_): mixture of two *E*/*Z* conformers (approximately 87:13) δ 7.56 (s, 1H, imidazole), 7.48–7.32 (m, 5H, aromatic), 7.25–7.15 (m, 4H, aromatic), 7.08 (s, 1H, imidazole), 6.86 (s, 1H, imidazole), 6.83 (s, 1H, CHN, conformer *E* (or *Z*)), 6.50 (s, 1H, CHN, conformer *Z* (or *E*)), 5.13 (s, 2H, COCH_2_, conformer *E* (or *Z*)), 5.10 (s, 2H, COCH_2_, conformer *Z* (or *E*)), 2.81 (s, 3H, NCH_3_, conformer *E* (or *Z*)), 2.61 (s, 3H, NCH_3_, conformer *Z* (or *E*)); ^13^C NMR (125 MHz, DMSO-*d*_6_): δ 168.04, 138.55, 138.11, 132.32, 130.52, 128.87, 128.71, 127.88, 127.69, 121.27, 60.17, 47.78, 31.18. Anal. calcd for: C_19_H_18_ClN_3_O: C, 67.15; H, 5.34; N, 12.37. Found: C, 67.33; H, 5.34; N, 12.35.

#### *N*-((3-Bromophenyl)(phenyl)methyl)-2-(1*H*-imidazol-1-yl)-*N*-methylacetamide (**7m**)

Colorless solid (50%): mp 82–83 °C; IR (neat) cm^–1^ 3375 (broad), 2968, 2361, 1660 (C=O stretch.), 1593, 1568, 1514, 1472, 1402, 1301, 1236, 1108, 1078, 826, 739, 703, 665; ^1^H NMR (500 MHz, DMSO-*d*_6_): mixture of two *E*/*Z* conformers (approximately 84:16) δ 7.56 (s, 1H, imidazole), 7.53 (d, *J* = 8.0 Hz, 1H, aromatic), 7.41–7.33 (m, 4H, aromatic), 7.31 (s, 1H, aromatic), 7.22–7.16 (m, 3H, aromatic), 7.08 (s, 1H, imidazole), 6.86 (s, 1H, imidazole), 6.84 (s, 1H, CHN, conformer *E* (or *Z*)), 6.52 (s, 1H, CHN, conformer *Z* (or *E*)), 5.18, 5.13 (ABq, *J*_AB_ = 20.0 Hz, 2H, COCH_2_), 2.83 (s, 3H, NCH_3_, conformer *E* (or *Z*)), 2.63 (s, 3H, NCH_3_, conformer *Z* (or *E*)); ^13^C NMR (125 MHz, DMSO-*d*_6_): δ 168.11, 141.99, 138.55, 138.37, 131.10, 130.92, 130.63, 128.87, 128.76, 127.94, 127.64, 122.09, 121.24, 60.31, 47.76, 31.32. Anal. calcd for: C_19_H_18_BrN_3_O: C, 59.39; H, 4.72; N, 10.94. Found: C, 59.52; H, 4.73; N, 10.94.

#### 2-(1*H*-Imidazol-1-yl)-*N*-((4-iodophenyl)(phenyl)methyl)-*N*-methylacetamide (**7n**)

White solid (74%): mp 69–71 °C; IR (neat) cm^–1^ 3447 (broad), 2361, 1654 (C=O stretch.), 1508, 1483, 1400, 1300, 1236, 1108, 1078, 1006, 829, 739, 702; ^1^H NMR (500 MHz, DMSO-*d*_6_): mixture of two *E*/*Z* conformers (approximately 84:16) δ 7.75 (d, *J* = 8.0 Hz, 2H, aromatic), 7.55 (s, 1H, imidazole), 7.44–7.33 (m, 3H, aromatic), 7.17 (d, *J* = 7.0 Hz, 2H, aromatic), 7.08 (s, 1H, imidazole), 6.99 (d, *J* = 8.0 Hz, 2H, aromatic), 6.86 (s, 1H, imidazole), 6.83 (s, 1H, CHN, conformer *E* (or *Z*)), 6.49 (s, 1H, CHN, conformer *Z* (or *E*)), 5.15 (s, 2H, COCH_2_, conformer *E* (or *Z*)), 5.12 (s, 2H, COCH_2_, conformer *Z* (or *E*)), 2.82 (s, 3H, NCH_3_, conformer *E* (or *Z*)), 2.62 (s, 3H, NCH_3_, conformer *Z* (or *E*)); ^13^C NMR (125 MHz, DMSO-*d*_6_): δ 167.82, 138.80, 138.33, 137.26, 130.78, 128.60, 128.52, 127.60, 120.96, 93.58, 60.05, 47.52, 30.96. Anal. calcd for: C_19_H_18_IN_3_O: C, 52.91; H, 4.21; N, 9.74. Found: C, 53.01; H, 4.22; N, 9.72.

#### *N*-((4-((4-Bromobenzyl)oxy)phenyl)(phenyl)methyl)-2-(1*H*-imidazol-1-yl)-*N*-methylacetamide (**7p**)

White solid (40%): mp 120–121 °C; IR (KBr) cm^–1^ 3447 (broad), 3031, 2934, 1655 (C=O stretch.), 1609, 1509, 1456, 1400, 1304, 1245, 1177, 1111, 1076, 1104, 849, 750, 699, 662, 622; ^1^H NMR (500 MHz, DMSO-*d*_6_): mixture of two *E*/*Z* conformers (approximately 82:18) δ 7.54 (s, 1H, imidazole), 7.45–7.31 (m, 7H, aromatic), 7.20–7.14 (m, 2H, aromatic), 7.08–7.00 (m, 4H, aromatic), 6.85 (s, 1H, imidazole), 6.80 (s, 1H, imidazole), 5.16–5.09 (m, 3H, CHN + COCH_2_), 3.41 (s, 2H, CH_2_O), 2.80 (s, 3H, NCH_3_, conformer *E* (or *Z*)), 2.61 (s, 3H, NCH_3_, conformer *Z* (or *E*)); ^13^C NMR (125 MHz, DMSO-*d*_6_): δ 167.70, 157.74, 139.21, 138.41, 137.08, 130.98, 130.02, 128.55, 128.23, 127.96, 127.79, 127.61, 127.39, 121.08, 114.84, 69.35, 59.91, 47.62, 30.89. Anal. calcd for: C_26_H_24_BrN_3_O_2_: C, 63.68; H, 4.93; N, 8.57. Found: C, 63.70; H, 4.94; N, 8.55.

#### Synthesis of 4-Benzyloxy-substituted Benzophenones **8o–p**

4-Hydroxyacetophenone (5 mmol) and K_2_CO_3_ (10 mmol) were suspended in acetone (20 mL), and then the appropriate benzyl bromide (10 mmol) and a catalytic amount of KI were added. The mixture was refluxed for 3 h. The reaction solvent was removed under vacuum, and the resulting solid was crystallized from ethanol.

#### (4-((4-Bromobenzyl)oxy)phenyl)(phenyl)methanone (**8p**)

Whitish crystals (85%): mp 101–103 °C; IR (KBr) cm^–1^ 3448 (broad), 2920, 2861, 1639 (C=O stretch), 1602, 1503, 1489, 1446, 1404, 1378, 1290, 1247, 1224, 1174, 1149, 1074, 1021, 1010, 939, 925, 845, 802, 740, 695, 609, 508; ^1^H NMR (200 MHz, DMSO-*d*_6_): δ 7.78–7.43 (m, 11H, aromatic), 7.17 (d, *J* = 8.0 Hz, 2H, aromatic), 5.22 (s, 1H, CH_2_O). Anal. calcd for: C_20_H_15_BrO_2_: C, 65.41; H, 4.12. Found: C, 65.27; H, 4.11.

#### General Procedure for the Synthesis of Formamides **9j–p**

In a sealed vial equipped with a stirring bar were added ketones **8j–p** (1 equiv), formic acid (1.25 equiv), and formamide (3 mL). The suspension was stirred under microwave irradiation for 90 minutes (150 W, 150 psi, 170 °C). The resulting hot solution was diluted with EtOAc (100 mL) and washed three times with brine (3×50 mL). The organic phase was dried with Na_2_SO_4_, filtered, and evaporated. The crude was crystallized with a mixture of CHCl_3_/n-hexane or purified by flash chromatography using a mixture of cyclohexane/EtOAc (7:3). Using this procedure, the following compounds have been synthesized.

#### *N*-(Cyclopentyl(phenyl)methyl)formamide (**9j**)

Crystallized from CHCl_3_/n-hexane. Whitish solid (57%): mp 65–68 °C; IR (KBr) cm^–1^ 3331, 2957, 2859, 1657 (C=O stretch), 1510, 1385, 1223, 755, 701, 525; ^1^H NMR (200 MHz, DMSO-*d*_6_): δ 8.62 (d, *J* = 10.0 Hz, 1H, CONH), 8.02 (s, 1H, CHO), 7.34–7.19 (m, 5H, aromatic), 4.69–4.59 (m, 1H, CHN), 2.29–2.13 (m, 1H, cyclopentyl), 1.77–0.99 (m, 8H, cyclopentyl). Anal. calcd for: C_13_H_17_NO: C, 76.81; H, 8.43; N, 6.89. Found: C, 76.70; H, 8.41; N, 6.91.

#### *N*-(Cyclohexyl(phenyl)methyl)formamide (**9k**)

Crystallized from CHCl_3_/n-hexane. Whitish solid (77%): mp 128–130 °C; IR (KBr) cm^–1^ 3334, 3061, 3029, 2923, 2847, 1660 (C=O stretch), 1513, 1444, 1385, 1286, 1232, 1199, 1183, 1138, 1074, 1012, 966, 913, 893, 834, 768, 754, 703, 678, 632, 573, 526; ^1^H NMR (200 MHz, DMSO-*d*_6_): δ 8.56 (d, *J* = 8.0 Hz, 1H, CONH), 8.06 (s, 1H, CHO), 7.36–7.22 (m, 5H, aromatic), 4.69–4.60 (m, 1H, CHN), 1.78–1.50 (m, 5H, cyclohexyl), 1.33–0.83 (m, 6H, cyclohexyl). Anal. calcd for: C_14_H_19_NO: C, 77.38; H, 8.81; N, 6.45. Found: C, 77.57; H, 8.83; N, 6.43.

#### *N*-((3-Bromophenyl)(phenyl)methyl)formamide (**9m**)

Crystallized from CHCl_3_/n-hexane. Pale beige solid (57%): mp 97–100 °C; IR (KBr) cm^–1^ 3118, 3023, 2885, 2366, 1676 (C=O stretch), 1652, 1541, 1388, 1244, 1193, 1075, 1027, 788, 761, 745, 705, 610; ^1^H NMR (200 MHz, DMSO-*d*_6_): δ 9.18 (d, J = 8.0 Hz, 1H, CONH), 8.17 (s, 1H, CHO), 7.51–7.27 (m, 9H, aromatic), 6.20 (d, *J* = 8.0 Hz, 1H, CHN). Anal. calcd for: C_14_H_12_BrNO: C, 57.95; H, 4.17; N, 4.83. Found: C, 57.81; H, 4.16; N, 4.84.

#### *N*-((4-Iodophenyl)(phenyl)methyl)formamide (**9n**)

Crystallized from CHCl_3_/n-hexane. Beige solid (50%): mp 142–144 °C; IR (KBr) cm^–1^ 3327, 3227, 3007, 2904, 2344, 1654 (C=O stretch.), 1522, 1493, 1450, 1397, 1379, 1222, 1062, 1007, 850, 814, 762, 741, 700, 604, 537; ^1^H NMR (200 MHz, DMSO-*d*_6_): δ 9.13 (d, *J* = 8.0 Hz, 1H, CONH), 8.16 (s, 1H, CHO), 7.71 (d, *J* = 8.2 Hz, 2H, aromatic), 7.39–7.22 (m, 5H, aromatic), 7.10 (d, *J* = 8.2 Hz, 2H, aromatic), 6.14 (d, *J* = 8.0 Hz, 1H, CHN). Anal. calcd for: C_14_H_12_INO: C, 49.87; H, 3.59; N, 4.15. Found: C, 49.76; H, 3.60; N, 4.14.

#### *N*-((4-(Benzyloxy)phenyl)(phenyl)methyl)formamide (**9o**)

Crystallized from CHCl_3_/n-hexane. Whitish solid (73%): mp 127–130 °C; IR (KBr) cm^–1^ 3331, 3030, 2865, 1658 (C=O stretch.), 1610, 1511, 1452, 1385, 1236, 1181, 1011, 916, 873, 826, 819, 760, 698, 614, 542; ^1^H NMR (200 MHz, DMSO-*d*_6_): δ 9.07 (d, *J* = 8.0 Hz, 1H, CONH), 8.14 (s, 1H, CHO), 7.45–7.17 (m, 12H, aromatic), 6.97 (d, *J* = 8.0 Hz, 2H, aromatic), 6.12 (d, *J* = 8.0 Hz, 1H, CHN), 5.07 (s, 2H, CH_2_O). Anal. calcd for: C_21_H_19_NO_2_: C, 79.47; H, 6.03; N, 4.41. Found: C, 79.65; H, 6.01; N, 4.40.

#### *N*-((4-((4-Bromobenzyl)oxy)phenyl)(phenyl)methyl)formamide (**9p**)

Purified by flash chromatography (7 cyclohexane/3 EtOAc). Whitish solid (35%): mp 146–149 °C; IR (KBr) cm^–1^ 3303, 3030, 2919, 2867, 1657 (C=O stretch.), 1610, 1596, 1511, 1490, 1450, 1390, 1236, 1180, 1070, 1012, 876, 826, 816, 801, 761, 734, 702, 582, 542, 510; ^1^H NMR (200 MHz, DMSO-*d*_6_): δ 9.04 (d, *J* = 8.0 Hz, 1H, CONH), 8.14 (s, 1H, CHO), 7.58 (d, *J* = 8.0 Hz, 2H, aromatic), 7.41–7.17 (m, 9H, aromatic), 6.96 (d, *J* = 8.0 Hz, 2H, aromatic), 6.12 (d, *J* = 8.0 Hz, 1H, CHN), 5.06 (s, 2H, CH_2_O). Anal. calcd for: C_21_H_18_BrNO_2_: C, 63.65; H, 4.58; N, 3.53. Found: C, 63.46; H, 4.59; N, 3.54.

### General Procedure for the Synthesis of *N*-Methylamines **5j–k** and **5o–p**

A two-necked round-bottom flask equipped with a stirring bar was filled with N_2_. A suspension of LiAlH_4_ in 1 M THF (6 equiv) was added into the flask. The proper formamide **6** (1 equiv) was dissolved in dry THF (10 mL) in an inert atmosphere and slowly dropped via syringe to the suspension. The reaction was refluxed for 2 h. Then, the reaction mixture was cooled at 0 °C with an ice bath, and an aqueous solution of 2 M NaOH (20 equiv) was carefully added. The mixture was left under stirring for 30 minutes. The reaction mixture was diluted with EtOAc (50 mL) and extracted with water (3×75 mL) and brine (1 × 100 mL). The organic phases were dried on Na_2_SO_4_, filtered, and evaporated under vacuum. The crude product was purified by flash chromatography or column chromatography using a Biotage chromatographic system with Biotage SNAP KP-Sil flash chromatography cartridges using gradient mixtures of CH_2_Cl_2_/MeOH. Using this procedure, the following compounds have been synthesized.

#### 1-Cyclopentyl-*N*-methyl-1-phenylmethanamine (**5j**)

Pale yellow oil (52%): IR (neat) cm^–1^ 3337 (broad) (N–H stretch), 2951, 2868, 2785, 1490, 1474, 1452, 1136, 836, 761, 701; ^1^H NMR (200 MHz, DMSO-*d*_6_): δ 7.34–7.15 (m, 5H, aromatic), 3.12 (d, *J* = 8.0 Hz, 1H, CHN), 2.03 (s, 3H, NCH_3_), 1.99–1.76 (m, 2H, cyclopentyl), 1.61–1.28 (m, 5H, cyclopentyl), 1.23–0.97 (m, 2H, cyclopentyl). Anal. calcd for: C_13_H_19_N: C, 82.48; H, 10.12; N, 7.40. Found: C, 82.23; H, 10.11; N, 7.42.

#### 1-Cyclohexyl-*N*-methyl-1-phenylmethanamine (**5k**)

Pale yellow oil (35%): IR (neat) cm^–1^ 3343 (broad) (N–H stretch), 2925, 2851, 2787, 1734, 1636, 1602, 1492, 1450, 1133, 1062, 1029, 891, 826, 760, 703, 666; ^1^H NMR (200 MHz, DMSO-*d*_6_): δ 7.34–7.15 (m, 5H, aromatic), 3.12 (d, J = 8.0 Hz, 1H, CHN), 2.03–1.76 (m, 6H, NCH_3_ + cyclohexyl), 1.61–0.97 (m, 8H, cyclohexyl). Anal. calcd for: C_14_H_21_N: C, 82.70; H, 10.41; N, 6.89. Found: C, 82.98; H, 10.40; N, 6.91.

#### 1-(4-(Benzyloxy)phenyl)-*N*-methyl-1-phenylmethanamine (**5o**)

Whitish solid (76%): mp 67–69 °C; IR (neat) cm^–1^ 3030 (N–H stretch), 2946, 2784, 1699, 1608, 1583, 1509, 1493, 1469, 1454, 1386, 1341, 1300, 1242, 1172, 1115, 1014, 912, 827, 808, 748, 697, 643, 630, 607, 548; ^1^H NMR (200 MHz, DMSO-*d*_6_): δ 7.45–7.15 (m, 12H, aromatic), 6.92 (d, J = 10.0 Hz, 2H, aromatic), 5.04 (s, 2H, CH_2_O), 4.58 (s, 1H, CHN), 2.19 (s, 3H, NCH_3_). Anal. calcd for: C_21_H_21_NO: C, 83.13; H, 6.98; N, 4.62. Found: C, 83.33; H, 6.97; N, 4.61.

#### 1-(4-((4-Bromobenzyl)oxy)phenyl)-*N*-methyl-1-phenylmethanamine (**5p**)

White solid (63%): mp 80–82 °C; IR (KBr) cm^–1^ 3448 (broad) (N–H stretch), 3323, 3032, 2931, 2838, 2786, 1607, 1584, 1508, 1470, 1455, 1379, 1298, 1234, 1170, 1125, 1106, 1016, 814, 732, 700, 620, 550; ^1^H NMR (200 MHz, DMSO-*d*_6_): δ 7.44–7.12 (m, 11H, aromatic), 6.92 (d, *J* = 10.0 Hz, 2H, aromatic), 5.04 (s, 2H, CH_2_O), 4.58 (s, 1H, CHN), 2.20 (s, 3H, NCH_3_). Anal. calcd for: C_21_H_20_BrNO: C, 65.98; H, 5.27; N, 3.66. Found: C, 65.89; H, 5.26; N, 3.67.

#### Synthesis of 1-(4-Iodophenyl)-*N*-methyl-1-phenylmethanamine (**5n**)

Formamide **9n** (0.7 mmol, 1 equiv) was added in a hot-flamed three-necked round-bottom flask filled with argon and equipped with a magnetic stirring bar and solubilized in dry THF (7 mL). To the clear solution, DIBAL-H in 1 M *n*-hexane (2.1 mL, 3 equiv) was carefully added through a dropping funnel at room temperature. The reaction mixture was refluxed for 3.5 h and then was left under stirring at room temperature for 16 h. Then, the reaction mixture was cooled at 0 °C with an ice bath, and a solution of 2 M NaOH (12 equiv) was added dropwise. After 30 minutes of stirring at room temperature, the aqueous phase was separated from the organic phase, and the former was extracted three times with EtOAc (3×75 mL). The combined organic phases were dried on Na_2_SO_4_, filtered, and concentrated under vacuum. The crude was purified by flash chromatography using gradient mixtures of cyclohexane/EtOAc as eluent. The desired product was obtained as a pale yellow oil (51%): IR (neat) cm^–1^ 3336 (N–H stretch.), 3059, 3025, 2948, 2850, 2788, 2360, 1647, 1558, 1541, 1479, 1456, 1397, 1126, 1103, 1006, 801, 749, 698; ^1^H NMR (200 MHz, DMSO-*d*_6_): δ 7.67–7.61 (m, 2H, aromatic), 7.40–7.18 (m, 7H, aromatic), 4.61 (s, 1H, CHN), 2.20 (s, 3H, NCH_3_). Anal. calcd for: C_14_H_14_IN: C, 52.03; H, 4.37; N, 4.33. Found: C, 51.91; H, 4.38; N, 4.32.

#### Synthesis of *N*-Benzyl-3-(1*H*-imidazol-1-yl)propan-1-amine (**10**)

In a two-necked round-bottom flask, a mixture of benzaldehyde (480 μL, 1 equiv), 3-(1*H*-imidazol-1-yl)propan-1-amine (591 μL, 1.05 equiv), sodium acetate (386 mg, 1 equiv), and 3 Å molecular sieves (860 mg) in anhydrous MeOH (12 mL) was stirred under N_2_ at room temperature overnight. Then, the temperature was lowered to 0 °C, and NaBH_4_ (182 mg, 1.02 equiv) was added portionwise for 1 h. The reaction mixture was left under stirring at room temperature for 3 additional hours. The mixture was filtered through a celite pad and washed with MeOH. The filtrate was concentrated *in vacuo*, and the residue was diluted with CH_2_Cl_2_ and washed with an aqueous solution of 10% NaOH and brine. The organic phase was dried with Na_2_SO_4_, filtered, and concentrated. The pale yellow oil thus obtained was used in the next step without any further purification.

#### Synthesis of *N*-(3-(1*H*-Imidazol-1-yl)propyl)-*N*-benzylacetamide (**11a**)

A stirred mixture of *N*-benzyl-3-(1*H*-imidazol-1-yl)propan-1-amine (**10**) (150 mg, 1 equiv), TEA (194.3 μL, 2 equiv), and a catalytic amount of DMAP (0.1 equiv) in anhydrous CH_2_Cl_2_ (3 mL) was cooled to 0 °C. Acetic anhydride (65.3 μL, 1 equiv) was added dropwise, and the mixture was stirred for 10 minutes at 0 °C and then at room temperature for 12 h. The mixture was diluted with CH_2_Cl_2_, washed with a saturated solution of NaHCO_3_ (50 mL), deionized water (50 mL) and brine (50 mL), dried with Na_2_SO_4_, filtered, and concentrated under vacuum. The residue was purified by flash chromatography eluting with a mixture of CH_2_Cl_2_/MeOH. Pale yellow oil (69%): IR (neat) cm^–1^ 3437 (broad), 2937, 1628 (C=O stretch), 1509, 1450, 1362, 1232, 1110, 1081, 1029, 983, 917, 822, 735, 699, 666; ^1^H NMR (500 MHz, DMSO-*d*_6_): mixture of two *E*/*Z* conformers (approximately 50:50): δ 7.59 (s, 1H, imidazole), 7.36 (t, *J* = 7.5 Hz, 1H, aromatic), 7.31–7.21 (m, 2H, aromatic), 7.18–7.13 (m, 3H, aromatic + imidazole), 6.87 (d, *J* = 10.0 Hz, 1H, imidazole), 4.52 (s, 2H, CH_2_Ar, conformer *E* (or *Z*)), 4.46 (s, 2H, CH_2_Ar, conformer *Z* (or *E*)), 3.95–3.89 (m, 2H, CH_2_-imidazole, conformer *E* (or *Z*) + conformer *Z* (or *E*)), 3.22 (t, *J* = 7.5 Hz, 2H, NCH_2_, conformer *E* (or *Z*)), 3.14 (t, *J* = 7.5 Hz, 2H, NCH_2_, conformer *Z* (or *E*)), 2.01 (s, 2H, COCH_3_, conformer *E* (or *Z*)), 1.99 (s, 2H, COCH_3_, conformer *Z* (or *E*)), 1.98–1.92 (m, 2H, CH_2_C*H*_*2*_CH_2_, conformer *E* (or *Z*)), 1.90–1.84 (m, 2H, CH_2_C*H*_*2*_CH_2_, conformer *Z* (or *E*)); ^13^C NMR (125 MHz, DMSO-*d*_6_): δ 170.58, 170.05, 138.23, 137.72, 137.37, 137.30, 128.91, 128.55, 128.34, 127.63, 127.43, 127.13, 126.63, 119.36, 51.29, 47.46, 45.05, 44.07, 43.67, 42.83, 29.45, 28.84, 21.67, 21.13. Anal. calcd for: C_15_H_19_N_3_O: C, 70.01; H, 7.44; N, 16.33. Found: C, 70.14; H, 7.46; N, 16.27.

### General Procedure for the Synthesis of *N*-(3-(1*H*-Imidazol-1-yl)propyl)-*N*-benzylamides **11b,c**

*N*-Benzyl-3-(1*H*-imidazol-1-yl)propan-1-amine (**10**) (1.1 equiv) and TEA (1.5 equiv) were dissolved in a round-bottom flask with dry CH_2_Cl_2_ (6 mL); then the appropriate acyl chloride (1 equiv) was added dropwise at room temperature. The reaction mixture was stirred at room temperature for 12 h. The mixture was diluted with 20 mL of CH_2_Cl_2_ and 10 mL of deionized water; the organic phase was washed twice with a saturated solution of NaHCO_3_ and once with brine, dried with Na_2_SO_4_, filtered, and concentrated under vacuum. The crude was purified by flash chromatography using a mixture of EtOAc/MeOH (9.5:0.5). Using this procedure, the following compounds have been synthesized.

#### *N*-(3-(1*H*-Imidazol-1-yl)propyl)-*N*-benzylbenzamide (**11b**)

Yellow oil (48%): IR (neat) cm^–1^ 3434 (broad), 3111, 2940, 1627 (C=O stretch), 1577, 1509, 1496, 1446, 1426, 1360, 1320, 1282, 1231, 1144, 1109, 1080, 1028, 978, 917, 821, 789, 736, 702; ^1^H NMR (500 MHz, DMSO-*d*_6_): mixture of two *E*/*Z* conformers (approximately 55:45) δ 7.63 (s, 1H, imidazole), 7.41–7.26 (m, 10H, aromatic), 7.17–7.14 (m, 1H, imidazole), 6.93–6.77 (m, 1H, imidazole), 4.68 (br s, 2H, ArCH_2_, conformer *E* (or *Z*)), 4.45 (br s, 2H, ArCH_2_, conformer *Z* (or *E*)), 4.00 (br s, 2H, CH_2_-imidazole, conformer *E* (or *Z*)), 3.76 (br s, 2H, CH_2_-imidazole, conformer *Z* (or *E*)), 3.32 (br s, 2H, NCH_2_, conformer *E* (or *Z*)), 3.07 (br s, 2H, NCH_2_, conformer *Z* (or *E*)), 2.00–1.95 (m, 2H, CH_2_C*H*_*2*_CH_2_); ^13^C NMR (125 MHz, DMSO-*d*_6_): δ 171.04, 137.69, 137.12, 136.45, 129.32, 128.67, 128.48, 128.35, 127.42, 127.24, 126.83, 126.40, 126.21, 119.22, 118.94, 51.85, 46.99, 45.70, 43.88, 43.36, 41.72, 29.16, 28.33. Anal. calcd for: C_20_H_21_N_3_O: C, 75.21; H, 6.63; N, 13.16. Found: C, 75.43; H, 6.64; N, 13.12.

#### *N*-(3-(1*H*-Imidazol-1-yl)propyl)-*N*-benzyl-2-phenylacetamide (**11c**)

Pale yellow oil (49%): IR (neat) cm^–1^ 3418 (broad), 3110, 2938, 1640 (C=O stretch), 1496, 1453, 1426, 1360, 1282, 1230, 1155, 1109, 1080, 1030, 916, 821, 733, 698, 665; ^1^H NMR (500 MHz, DMSO-*d*_6_): mixture of two *E*/*Z* conformers (approximately 50:50) δ 7.57 (s, 1H, imidazole, conformer *E* (or *Z*)), 7.56 (s, 1H, imidazole, conformer *Z* (or *E*)), 7.37–7.11 (m, 10H + 1H, aromatic + imidazole), 6.91 (s, 1H, imidazole, conformer *E* (or *Z*)) 6.85 (s, 1H, imidazole, conformer *Z* (or *E*)), 4.60 (s, 2H, CH_2_Ar, conformer *E* (or *Z*)), 4.50 (s, 2H, CH_2_Ar, conformer *Z* (or *E*)), 3.93–3.87 (m, 2H, CH_2_-imidazole, conformer *E* (or *Z*) + conformer *Z* (or *E*)), 3.70 (s, 2H, ArCH_2_CO, conformer *E* (or *Z*)), 3.66 (s, 2H, ArCH_2_CO, conformer *Z* (or *E*)), 3.23 (t, *J* = 5.0 Hz, 2H, NCH_2_, conformer *E* (or *Z*)), 3.18 (t, *J* = 5.0 Hz, 2H, NCH_2_, conformer *Z* (or *E*)), 1.95–1.84 (m, 2H, CH_2_C*H*_*2*_CH_2_, conformer *E* (or *Z*) + conformer *Z* (or *E*)); ^13^C NMR (125 MHz, DMSO-*d*_6_): δ 170.71, 170.26, 138.09, 137.49, 137.24, 137.13, 135.80, 135.77, 129.07, 129.01, 128.71, 128.55, 128.37, 128.30, 127.47, 127.31, 126.99, 126.64, 126.43, 119.16, 50.57, 47.40, 44.32, 43.80, 43.40, 42.83, 29.42, 28.60. Anal. calcd for: C_21_H_23_N_3_O: C, 75.65; H, 6.95; N, 12.60. Found: C, 75.83; H, 6.97; N, 12.56.

### Molecular Modeling

The X-ray crystal structures of the cocrystal HO-1/QC-80 (PDB code 3HOK), cocrystal HO-1/QC-308 (PDB code 3TGM), and HO-2 (PDB code 2QPP) were used as protein structures. To validate the docking model, we used the procedure of our already published HO-1 inhibitor paper.^51^ The three-dimensional structures of all of the studied molecules were generated using Marvin Sketch (18.24, ChemAxon Ltd., Budapest, Hungary), and all geometries were fully optimized, in the same software, with the semiempirical PM6 Hamiltonian implemented in MOPAC2016 (17.130 W).^52−54^ Proteins and ligands were prepared within YASARA; the point charges were initially assigned according to the AMBER14 force field and then damped to mimic the less polar Gasteiger charges used to optimize the AutoDock scoring function.^55^ Fine docking was performed by applying the Lamarckian genetic algorithm (LGA) implemented in AutoDock. The ligand-centered maps were generated by the program AutoGrid with a spacing of 0.375 Å and dimensions that encompass all atoms extending 5 Å from the surface of the ligand. All of the parameters were inserted at their default settings. In the docking tab, the macromolecule and ligand are selected, and GA parameters are set as ga_runs = 100, ga_pop_size = 150, ga_num_evals = 20 000 000, ga_num_generations = 27 000, ga_elitism = 1, ga_mutation_rate = 0.02, ga_crossover_rate = 0.8, ga_crossover_mode = two points, ga_cauchy_alpha = 0.0, ga_cauchy_beta = 1.0, and number of generations for picking worst individual = 10. All protein amino acidic residues were kept rigid, whereas all single bonds of the ligands were treated as fully flexible.

### *In Vitro* ADMET Testing

In vitro ADMET testing has been performed at Eurofins Scientific. Aqueous solubility in simulated gastric fluid (catalogue product number 2061), intrinsic clearance in liver microsome-human (catalogue product number 607), CYP2D6 and CYP3A4 inhibition (catalogue product numbers 1338 and 391, respectively), and hERG human potassium ion channel binding (catalogue product number 4094) were conducted at Eurofins following their experimental protocols (https://www.eurofins.com/).

### Isosteric Replacement and Compound Alignment for the 3D-QSAR Evaluation

The isosteric replacement was performed using Spark as a software (10.4.0, Cresset, Litlington, Cambridgeshire, U.K., http://www.cresset-group.com/forge). The replacement was performed through the same 178,558 fragments for each part, which derive from ChEMBL and Zinc databases, as already reported.^56,57^ Two hundred compounds were generated for each substitution. Then, the newly designed molecules were imported into the software Forge (v10.4.2) for the alignment/evaluation of the data set in the 3D-QSAR model already published.^58^ The field points of each compound (negative, positive, shape, and hydrophobic) were calculated and generated using the XED (extended electron distribution) force field in Forge, and then the molecules were aligned with the training set of the QSAR model by a maximum common substructure algorithm using a customized and validated setup.^59,60^ The maximum number of conformations generated for each molecule was set to 500. The root-mean-square deviation of the atomic position cutoff, which is the similarity threshold below which two conformers are assumed identical, was set to 0.5 Å. The gradient cutoff for conformer minimization was set to 0.1 kcal/mol. The energy window was set to 2.5 kcal/mol, and all of the conformers with the calculated energy outside the selected energy window were discarded.

### Preparation of Spleen and Brain Microsomal Fractions

HO-1 and HO-2 were obtained, respectively, from the rat spleen and brain as the microsomal fraction prepared by differential centrifugation; the dominance of HO-1 protein in the rat spleen and HO-2 in the rat brain has been well documented.^24^ These particular microsomal preparations were selected to use the most native (*i.e*., closest to *in vivo*) forms of HO-1 and HO-2. Spleen and brain (Sprague-Dawley rats) microsomal fractions were prepared according to the procedure outlined by Ryter et al.^2^ The experiments reported in the present paper complied with the current Italian law and met the guidelines of the Institutional Animal Care and Use Committee of the Ministry of Health (Directorate General for Animal Health and Veterinary Medicines, Italy) “Dosing of enzymatic activities in rat microsomes” (2018–2022), project code 02769.N.VLY. The experiments were performed in male Sprague-Dawley albino rats (150 g body weight and age 45 d). They had free access to water and were kept at room temperature with a natural photoperiod (12 h light–12 h dark cycle). For measuring HO-1 and HO-2 activities, each rat was sacrificed and their spleen and brain were excised and weighed. A homogenate (15%, w/v) of spleens and brains pooled from four rats was prepared in ice-cold HO-homogenizing buffer (50 mM Tris buffer, pH 7.4, containing 0.25 M sucrose) using a Potter–Elvehjem homogenizing system with a Teflon pestle. The microsomal fraction of rat spleen and brain homogenate was obtained by centrifugation at 10 000*g* for 20 min at 4 °C, followed by centrifugation of the supernatant at 100 000*g* for 60 min at 4 °C. The 100 000*g* pellet (microsomes) was resuspended in 100 mM of potassium phosphate buffer, pH 7.8, containing 2 mM MgCl_2_ with a Potter–Elvehjem homogenizing system. The rat spleen and brain microsomal fractions were divided into equal aliquots, placed into microcentrifuge tubes, and stored at −80 °C for up to 2 months.

### Preparation of Biliverdin Reductase

Liver cytosol has been used as a source of biliverdin reductase. Rat liver was perfused through the hepatic portal vein with cold 0.9% NaCl; then, it was cut and flushed with 2 × 20 mL of ice-cold PBS to remove all of the blood. Liver tissue was homogenized in 3 volumes of a solution containing 1.15% KCl w/v and Tris buffer 20 mM, pH 7.8 on ice. Homogenates were centrifuged at 10 000*g* for 20 min at 4 °C. The supernatant was decanted and centrifuged at 100 000*g* for 1 h at 4 °C to sediment the microsomes. The 100 000*g* supernatant was saved and then stored in small amounts at −80 °C after its protein concentration was measured.

### Measurement of HO-1 and HO-2 Enzymatic Activities in Microsomal Fraction of Rat Spleen and Brain

The HO-1 and HO-2 activities were determined by measuring the bilirubin formation using the difference in absorbance at 464–530 nm, as described by Ryter et al.^2^ Reaction mixtures (500 μL) consisted of 20 mM of Tris–HCl, pH 7.4, (1 mg/mL) microsomal extract, 0.5–2 mg/mL of biliverdin reductase, 1 mM of NADPH, 2 mM of glucose 6-phosphate (G6P), 1 U of G6P dehydrogenase, 25 μM of hemin, and 10 μL of DMSO (or the same volume of DMSO solution of test compounds to a final concentration of 100, 10, and 1 μM). Incubations were carried out for 60 min at 37 °C in a circulating water bath in the dark. Reactions were stopped by adding the same volume of chloroform. After recovering the chloroform phase, the amount of bilirubin formed was measured with a double-beam spectrophotometer as OD464–530 nm (extinction coefficient, 40 mM/cm^–1^ for bilirubin). One unit of the enzyme was defined as the amount of enzyme catalyzing the formation of 1 nmol of bilirubin/mg protein/h.

### Cell Culture and Treatments

Experiments were performed on human GBM cell lines U87MG (ATCCC number #HTB-14) and A172 (ATCCC #CRL-1620) on DU145 (ATCC HTB-81) and human lung adenocarcinoma A549 (ATCC CCL-185-LUC2). All cell lines were obtained from the American Type Culture Collection (ATCC, Rockville, MD). Cells were grown in Dulbecco’s modified Eagle’s medium (DMEM) supplemented with 10% of heat-inactivated fetal bovine serum (FBS), 100 U/mL of penicillin, and 100 μg/mL of streptomycin (Sigma-Aldrich, Steinheim, Germany). Cells were incubated at 37 °C in a humidified atmosphere with 5% CO_2_.

### Cell Viability Assay

The effect of selected compounds **7i** and **7l**–**p** on cell viability was assessed by performing an MTT assay. Cells were seeded into 96-well plates at a density of 7 × 10^3^ cells/well in 100 μL of culture medium. After 24 h, cells were treated with selected compounds at three different concentrations (1 μM, 10 μM, and 50 μM) for 48 h. Following treatments, 0.5 mg/mL of 3-[4,5-dimethylthiazol-2-yl]-2,5-diphenyltetrazolium bromide (MTT) (Sigma-Aldrich) was added to each well and incubated for 4 h at 37 °C. Finally, dimethyl-sulfoxide (DMSO) was used to dissolve formazan salts, and absorbance was measured at 570 nm in a microplate reader (Biotek Synergy-HT). Eight replicate wells were used for each group.

### Western Blot Analysis

Proteins were extracted from total cell lysate, as previously described.^61^ Briefly, cell lines were added to a buffer containing 20 mM Tris (pH 7.4), 2 mM EDTA, 0.5 mM EGTA, 50 mM mercaptoethanol, and 0.32 mM sucrose supplemented with phosphatase and protease inhibitor cocktails (Roche Diagnostics, Monza, Italy). Subsequently, protein samples were sonicated twice for **20′** by using an ultrasonic probe, followed by centrifugation at 10 000*g* for 10 min at 4 °C. Sample proteins (30 μg) were diluted in 4X NuPage LDS sample buffer (Invitrogen, NP0007), heated at 80 °C for 5 min, and then separated on a Biorad Criterion XT 4–15% Bis–Tris gel (BIO-RAD) by electrophoresis and then transferred to a PVDF membrane (BIO-RAD). Blots were blocked using the Odyssey Blocking Buffer (LI-COR Biosciences) and probed with appropriate antibodies: HO-1 antibody (GeneTex GTX101147), goat polyclonal anti-VEGF (cat. no. sc-1836, Santa Cruz Biotechnology), and rabbit polyclonal anti-β-tubulin (cat n.sc-9104, Santa Cruz Biotechnology) and β-actin antibody (GeneTex GTX109639).

The secondary antibodies goat antirabbit (Odyssey, #926-32211; Odyssey #926-68021) and donkey antigoat IRDye 800CW (cat #926-32214; Li-Cor Biosciences) were used at 1:7000. Blots were scanned, and densitometric analysis was performed with the Odyssey Infrared Imaging System. Values were normalized to β-actin and to β-tubulin.

### Immunolocalization

To determine the cellular distribution of heme oxygenase-1 in tumor cells, immunofluorescence analysis was performed as previously described.^62^ Tumor cell lines were cultured on glass coverslips fixed in 4% paraformaldehyde in PBS (15′ at room temperature), permeabilized with 0.2% of Triton X100, blocked with 0.1% of BSA in PBS, and then probed with rabbit HO-1 antibody. The signal was revealed with Alexa Fluor 488 goat antirabbit for 1 h at room temperature and maintained shielded from light. DNA was counterstained stained with DAPI (#940110, Vector Laboratories). After a series of PBS and double-distilled water washes, the fixed cells were cover-slipped with the Vectashield mounting medium (Vector Laboratories, Inc., Burlingame, CA). Localization of the antibody was performed by Zeiss fluorescent microscopy.

### Measurement of HO Enzymatic Activity in U87MG Cell Line

U87MG cells were incubated for 24 h in the presence or absence of 10 μM of compound **7l**. Total HO activity in the cell lysate was determined by measuring the bilirubin formation using the difference in absorbance at 464–530 nm.^2^ Reaction mixtures (500 μL) consisted of 20 mM of Tris–HCl, pH 7.4, 1 mg/mL of cell lysate, 0.5–2 mg/mL of biliverdin reductase, 1 mM of NADPH, 2 mM of glucose 6-phosphate (G6P), 1 U of G6P dehydrogenase, 25 μM of hemin, and 10 μL of DMSO. Incubations were carried out for 60 min at 37 °C in a circulating water bath in the dark. Reactions were stopped by adding 1 volume of chloroform. After recovering the chloroform phase, the amount of bilirubin formed was measured with a double-beam spectrophotometer as OD464–530 nm (extinction coefficient, 40 mM/cm for bilirubin). One unit of the enzyme was defined as the amount of enzyme catalyzing the formation of 1 nmol of bilirubin/mg protein/h.

### Wound-Healing Assay

U87MG cells grown to confluence in 6-well dishes (5 × 10^4^ cells/well) were scratched with a 200 μL pipette tip. Cells were cultured in a 1% serum medium with or without 10 μM of **7l**. Quantitative assessment of the wound area was performed under an inverted microscope, as previously described.^63^ The distance that the advancing cells had moved into the wound area was measured after 24 and 48 h. The migration was calculated as the average number of cells observed in three random of high-power wounded fields/per well in duplicate wells and expressed in percentage of control (% of control).

### ELISA

VEGF-A release in conditioned media was measured using the ELISA sandwich enzymatic method with specific anti-VEGF-A (cat. No. ELH-VEGF) antibodies coated on a 96-well plate, according to the manufacturer’s guidelines. Briefly, confluent U87MG cells grown in media supplemented with 1% FBS were treated for 48 h with 10 μM of **7l**. Standards and supernatants from samples were pipetted into the wells containing the immobilized VEGF antibody. The wells were then washed before adding a biotinylated antihuman VEGF antibody. Following incubation, the unbound biotinylated antibody was washed off, and HRP-conjugated streptavidin was pipetted in each well. After an additional wash step, a TMB substrate solution was added to each well, resulting in blue coloration proportional to the amount of bound VEGF. Finally, the stop solution was added, and the colorimetric intensity of the blue substrate now turned yellow was measured at 450 nm.

### Conditioned Medium and Tube Formation Assay

Subconfluent U87MG cell cultures were placed in media supplemented with 1% FBS (representing the conditioned medium 1 (CM1) or control) or containing 10 μM of **7l** molecule (representing the conditioned medium 2 (CM2)) and incubated at 37 °C for 48 h. Subsequently, the CMs were collected and centrifuged at 2000 rpm for 5 min, and the supernatants were frozen at −80 °C until use. GeltrexTM reduced growth factor basement membrane matrix (Invitrogen) was thawed at 4 °C overnight before use. GeltrexTM matrix was added to a 24-well plate (95 μL/well) and then incubated at 37 °C for 30 min to allow polymerization. Murine microvascular endothelial cells (H5V) were starved overnight in the growth medium, and then the cells were seeded onto the layer of GeltrexTM matrix and cultured with 200 μL of CM1 and CM2 at 37 °C for 48 h. Three randomly selected fields of view were captured with a digital camera (Canon) connected to an inverted microscope (Axio Observer A1; Carl Zeiss, Göttingen, Germany). Tube numbers per field were calculated as the percentage of control.

### Statistical Analyses

Data are represented as mean ± standard error (SEM). One-way analysis of variance (ANOVA) was used to compare differences among groups, and statistical significance was assessed by the Tukey–Kramer post hoc test. The level of significance for all statistical tests was set at *p* ≤ 0.05.

## Supporting Information Available

Molecular formula strings (CSV)
^1^H and ^13^C NMR spectra; BOILED-Egg plot; series 1 and 2 of the scaffold-hopping analysis in **7l**; results of SwissADME calculations; results of pkCSM calculations; series 1 and 2 derived from isosteric replacement (PDF)
3D PDB file for Figure 3 (PDB)
3D PDB file for Figure 4 (PDB)
3D PDB file for Figure 5 (PDB)
3D PDB file for Figure 6 (PDB)
3D PDB file for Figure 7 (PDB)

(CSV)
(PDF)
(PDB)
(PDB)
(PDB)
(PDB)
(PDB)

## Supplementary Material

jm1c00633_si_002.pdf

jm1c00633_si_003.pdb

jm1c00633_si_004.pdb

jm1c00633_si_005.pdb

jm1c00633_si_006.pdb

jm1c00633_si_007.pdb

## Abbreviations Used

BSA: bovine serum albumin
CH_3_CN: acetonitrile
DAPI: 4′,6-diamidino-2-phenylindole
DIBAL-H: diisobutylaluminum hydride
DMAP: 4-dimethylaminopyridine
DMF: *N*,*N*-dimethylformamide
DMSO: dimethyl sulfoxide
HCOOH: formic acid
H2NCHO: formamide
HO-1: heme oxygenase-1
HO-2: heme oxygenase-2
G6P: glucose 6-phosphate
GBM: glioblastoma
IC_50_: inhibitory concentration_50_
MPs: metalloporphyrins
MTT: 3-(4,5-dimethylthiazol-2-yl)-2,5-diphenyl-2*H*-tetrazolium bromide
NaH: sodium hydride
NADPH: nicotinamide adenine dinucleotide phosphate
NMR: nuclear magnetic resonance
PBS: phosphate-buffered saline
RMSD: root-mean-square deviation
SAR: structure–activity relationship
SD: standard deviation
TEA: triethylamine
THF: tetrahydrofuran
TLC: thin-layer chromatography
VEGF: vascular endothelial growth factor
