# Supplementary material for: Discovery of Novel Acetamide-Based Heme Oxygenase-1 Inhibitors with Potent In Vitro Antiproliferative Activity
Source: J Med Chem. 2021 Sep 2;64(18):13373–93. doi: 10.1021/acs.jmedchem.1c00633 (PMC8474116; doi:10.1021/acs.jmedchem.1c00633)
Supplement: Supplementary file 2 — jm1c00633_si_002.pdf [file jm1c00633_si_002.pdf]

SUPPORTING INFORMATION

Discovery of novel acetamide-based heme oxygenase-1 inhibitors with potent *in vitro* antiproliferative activity

Antonino N. Fallica, [a ‡] Valeria Sorrenti, [a ‡] Agata G. D'Amico, [a] Loredana Salerno, [a] Giuseppe Romeo, [a] Sebastiano Intagliata, [a] Valeria Consoli, [a] Giuseppe Floresta, [b] Antonio Rescifina, [a] Velia D'Agata, [c] Luca Vanella, [a] Valeria Pittalà [a] *

[a] Department of Drug and Health Sciences, University of Catania, 95125 Catania, Italy.

[b] Department of Analytics, Environmental & Forensics, King's College London, Stamford Street, London SE1 9NH, UK

[c] Sections of Human Anatomy and Histology, Department of Biomedical and Biotechnological Sciences, University of Catania, 95123 Catania, Italy

KEYWORDS. Heme oxygenase-1, heme oxygenase-2, structure-activity relationships, inhibitors, glioblastoma, U87MG.

Corresponding Author

* Valeria Pittalà. Department of Drug and Health Sciences, University of Catania, Viale A. Doria 6, 95125, Catania, Italy. Phone +39 0957384269. Email: vpittala@unict.it. Orcid: 0000-0003-1856-0308

Table of contents

NMR spectra of compounds S2-S18
Figure S35. BOILED-Egg plot S19
Table S2. Results of SwissADME calculations S19-S21
Table S3. Results of pkCSM calculations S21-S23
Figure S36. Series 1 and 2 of the scaffold-hopping analysis in **7l**. S23
Table S4. Series 1 derived from isosteric replacement S23-S25
Table S5. Series 2 derived from isosteric replacement S25-S26

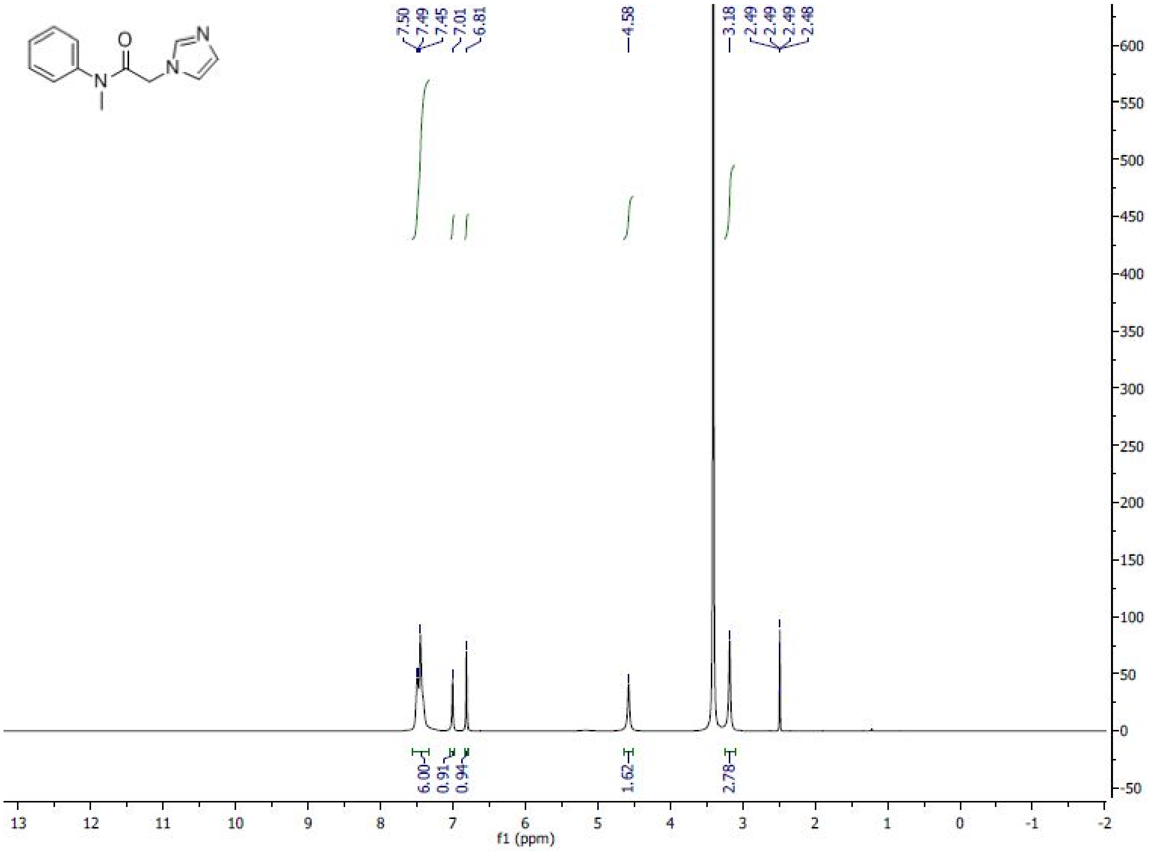

Figure S1. ^{1}H NMR (500 MHz, DMSO-d_6) of compound 7b.

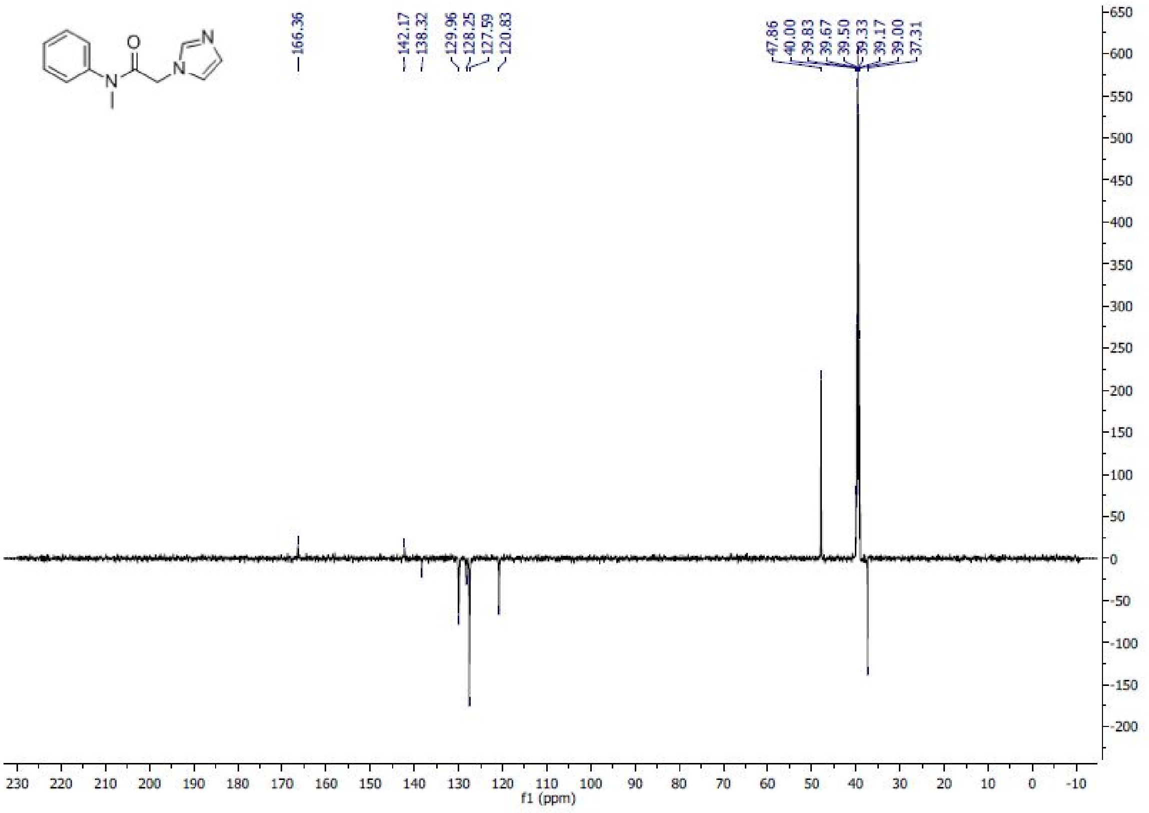

Figure S2. ^{13}C NMR (125 MHz, DMSO-d_6) of compound 7b.

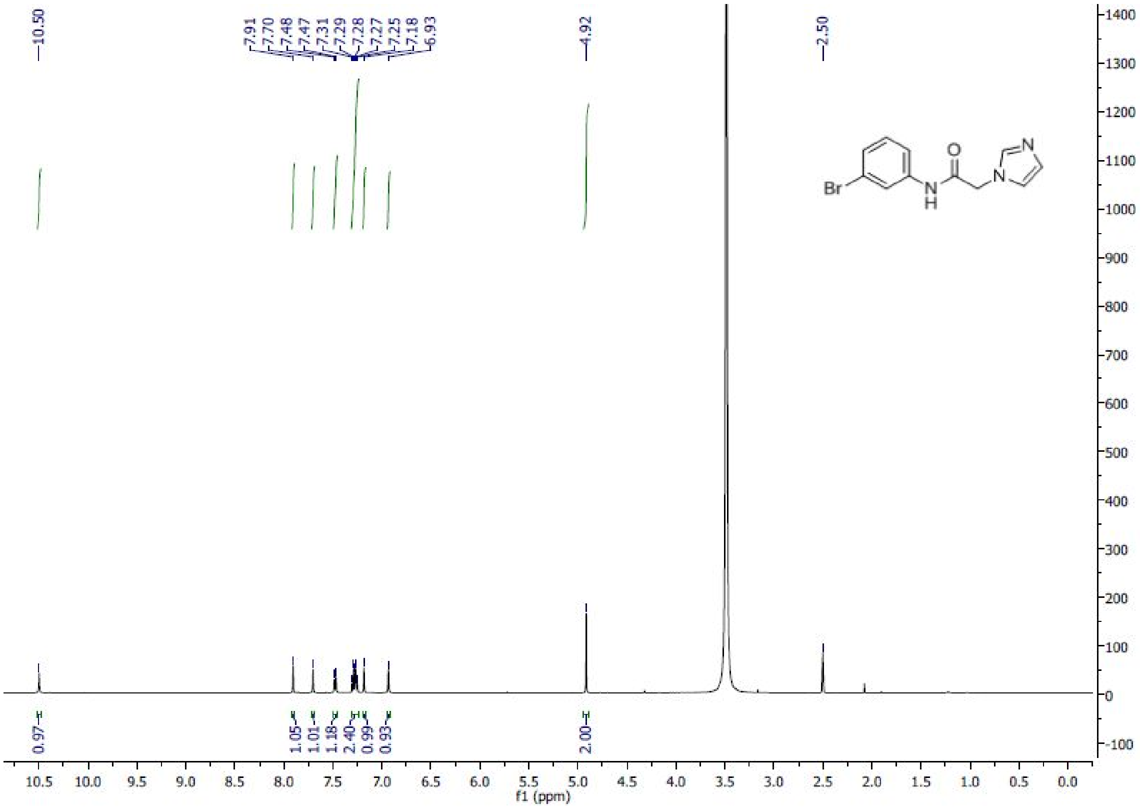

Figure S3. ¹H NMR (500 MHz, DMSO-d_6) of compound 7c.

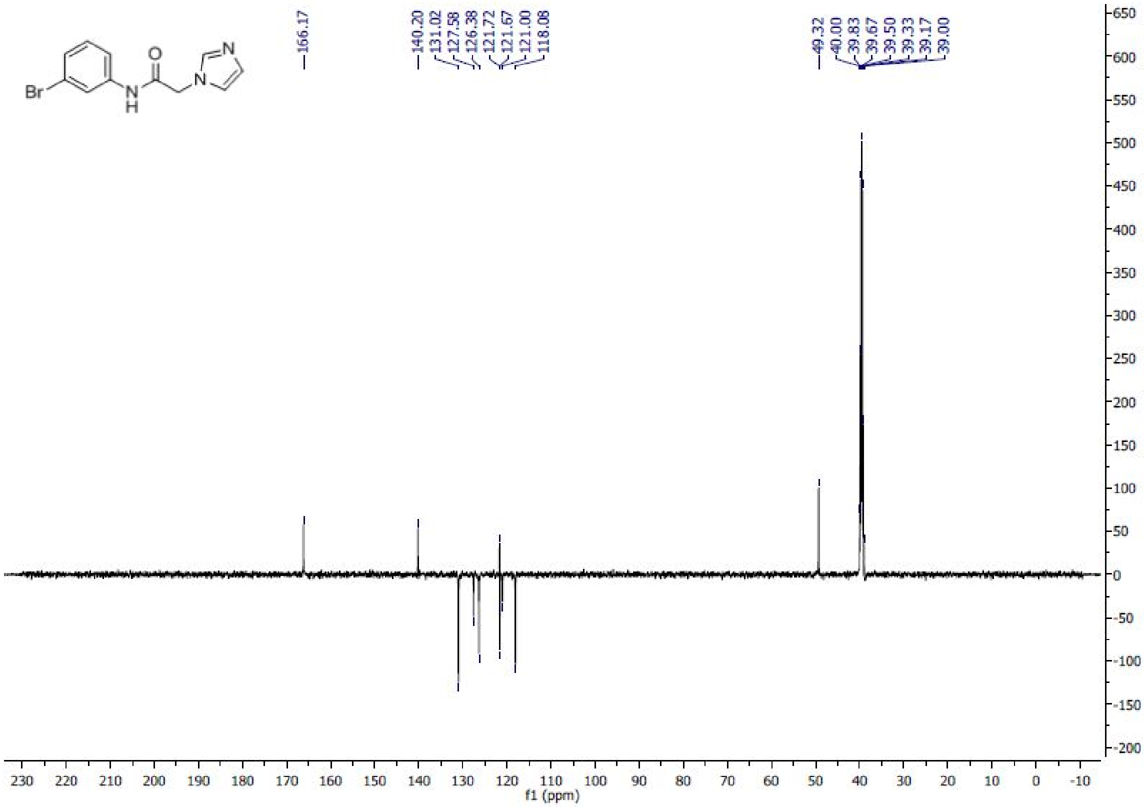

Figure S4. ^{13}C **NMR (125 MHz, DMSO-d_6) of compound 7c.**

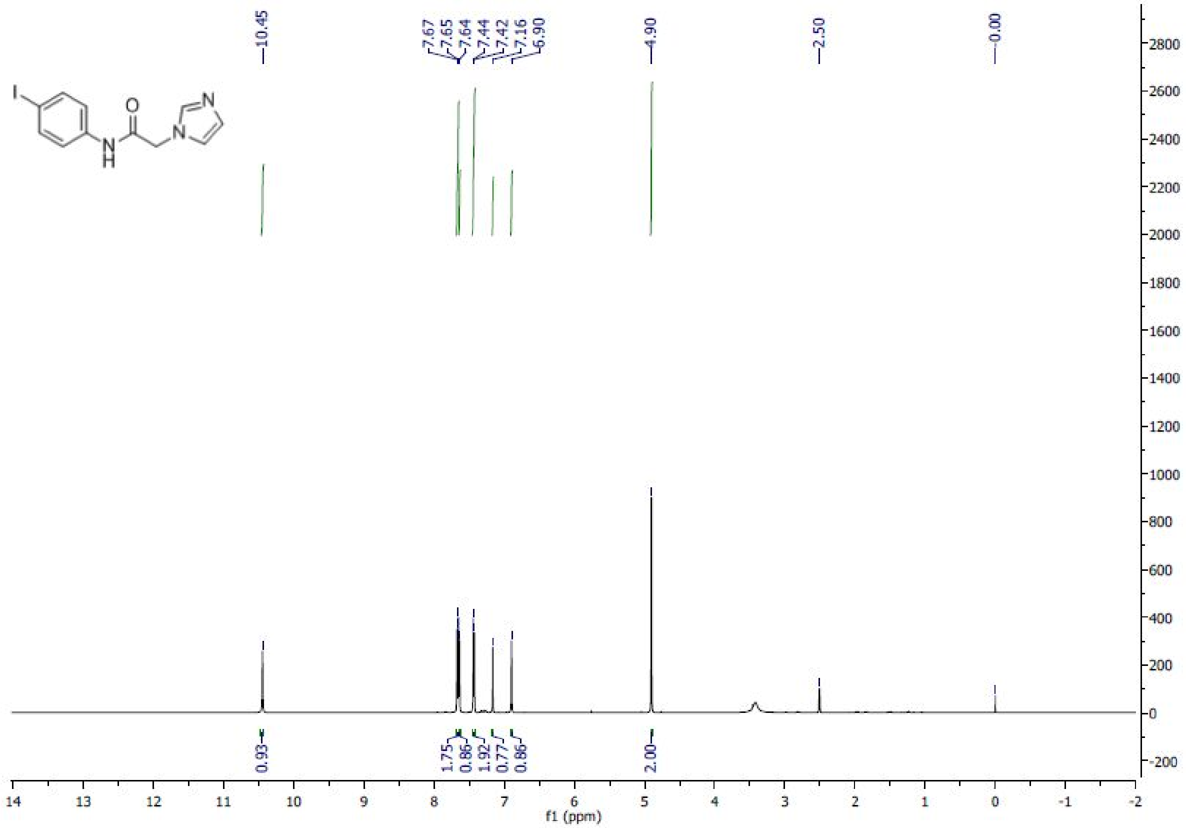

Figure S5. ^{1}H **NMR (500 MHz, DMSO-d_6) of compound 7d.**

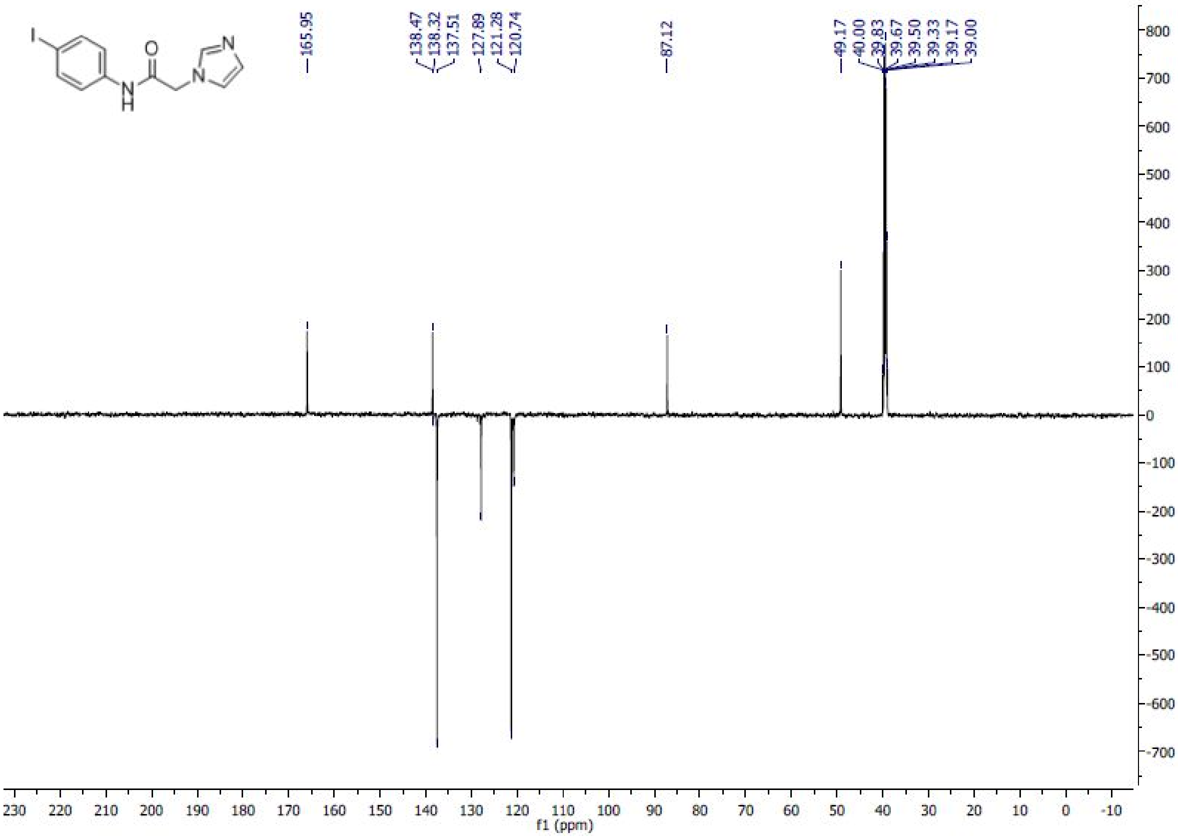

Figure S6. ¹³C NMR (125 MHz, DMSO-d_6) of compound 7d.

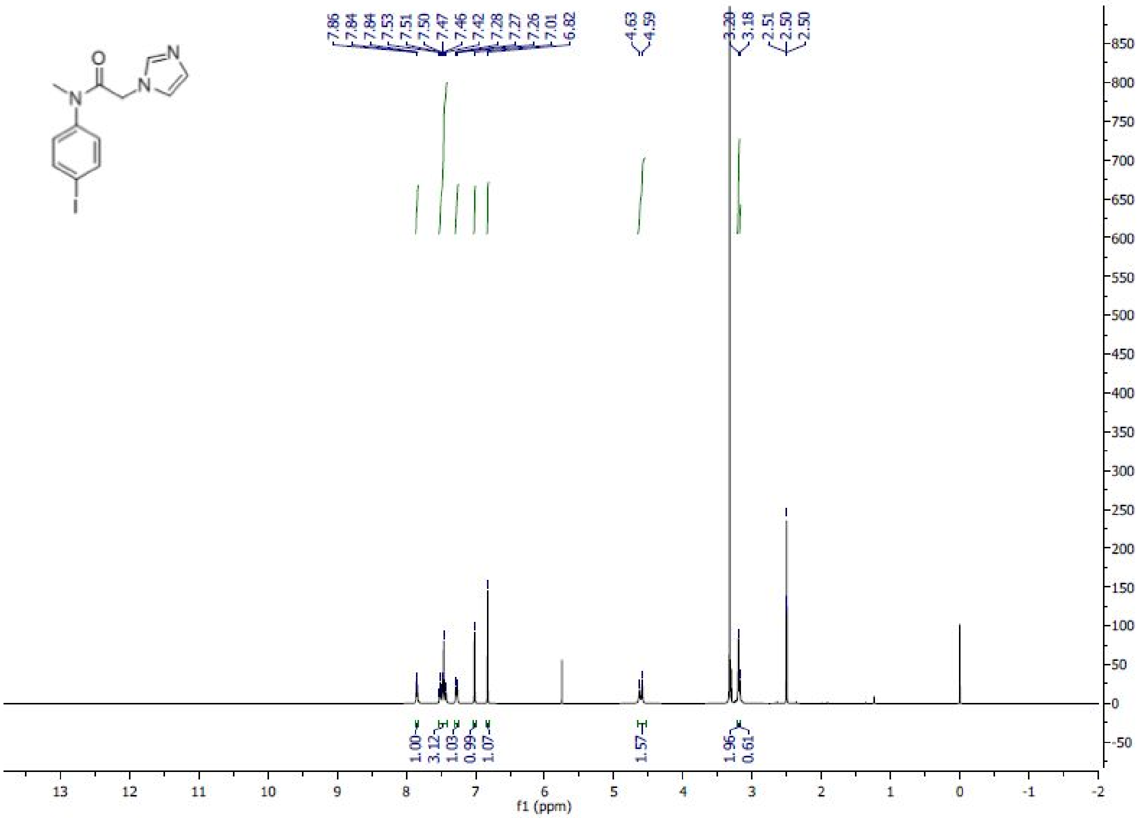

Figure S7. ¹H NMR (500 MHz, DMSO-d_6) of compound 7e.

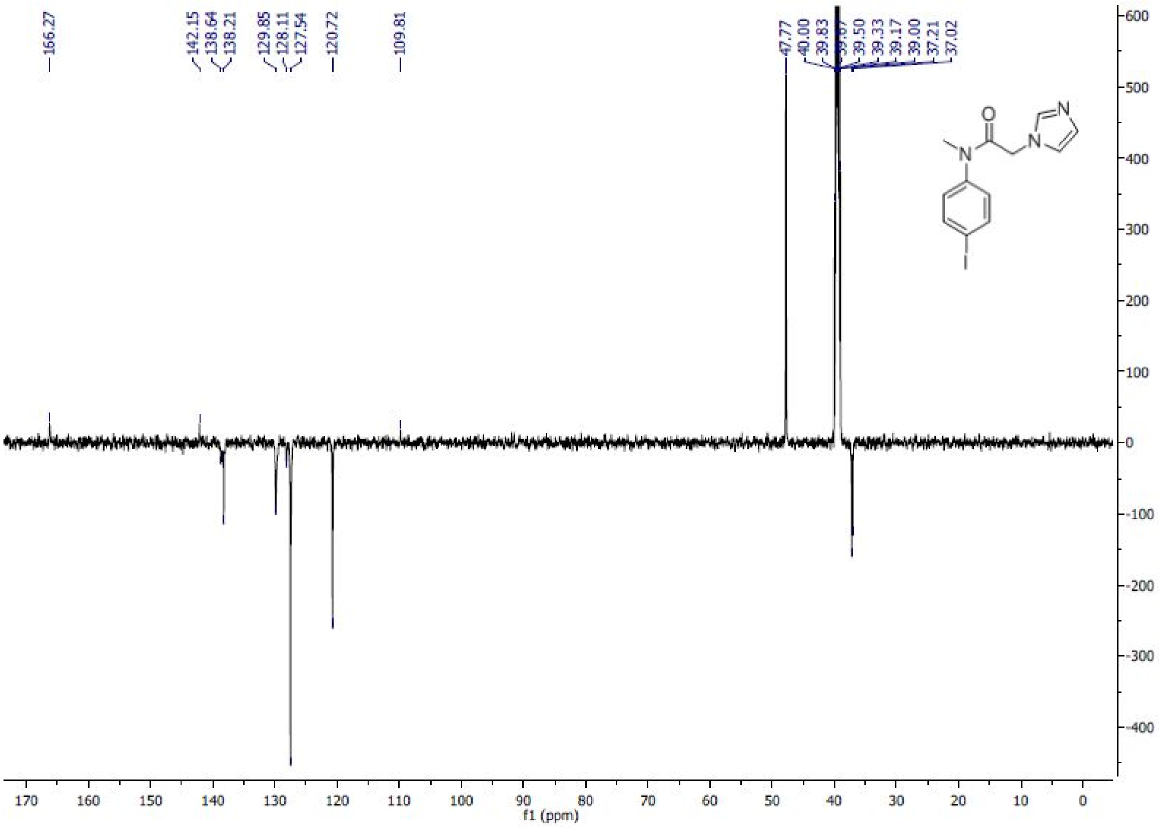

Figure S8. ^{13}C NMR (125 MHz, DMSO-d_6) of compound 7e.

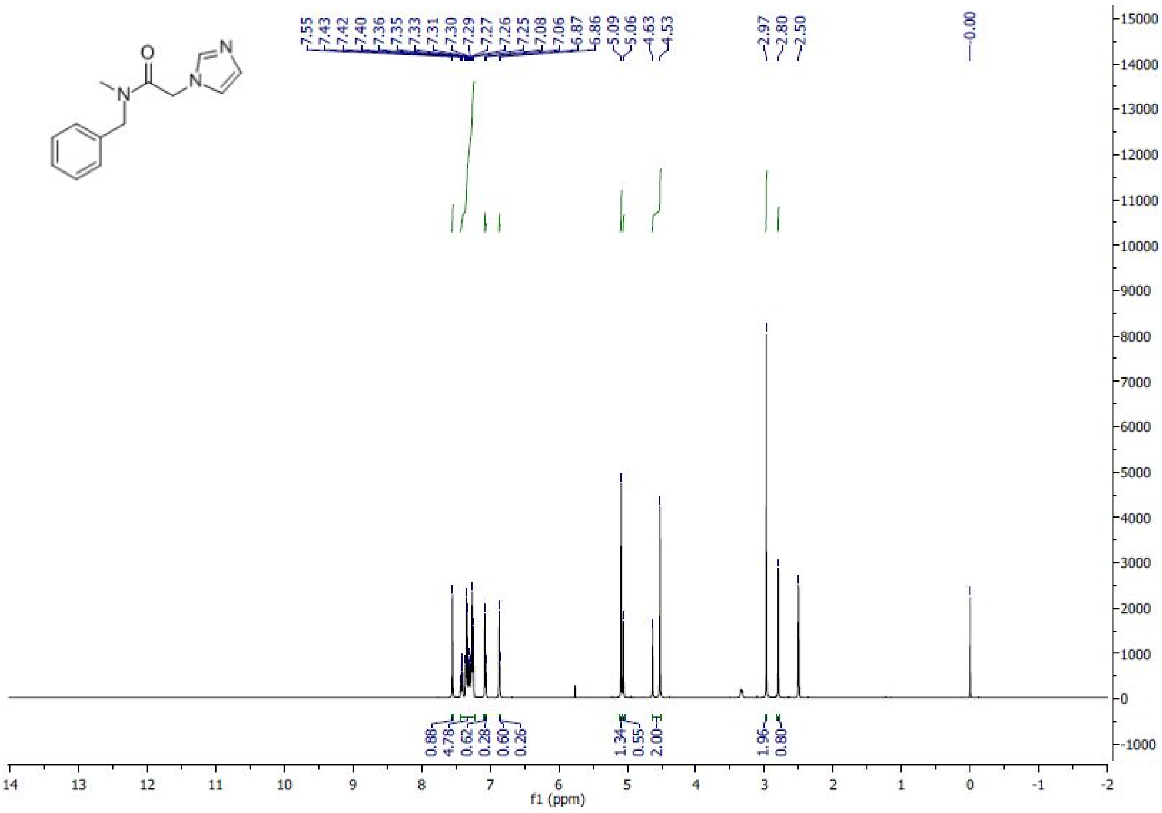

Figure S9. ^{1}H NMR (500 MHz, DMSO-d_6) of compound 7g.

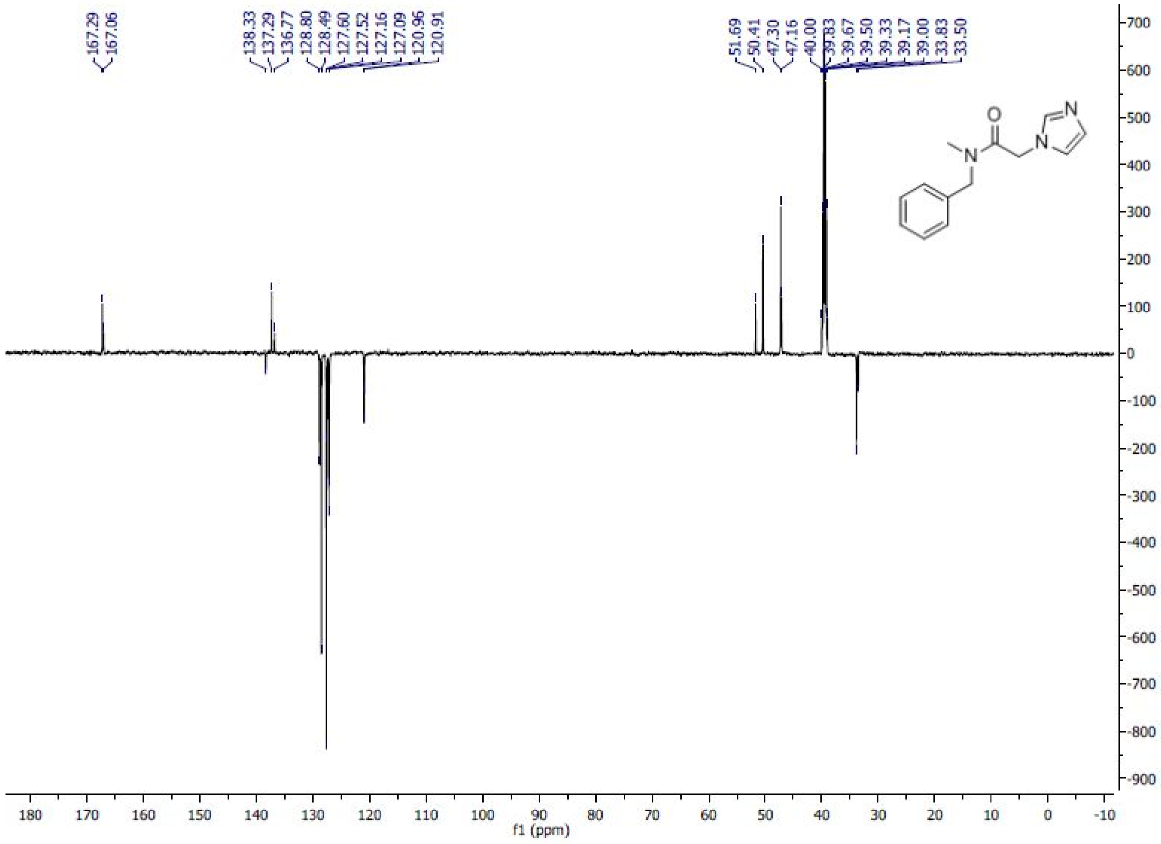

Figure S10. ^{13}C NMR (125 MHz, DMSO-d_6) of compound 7g.

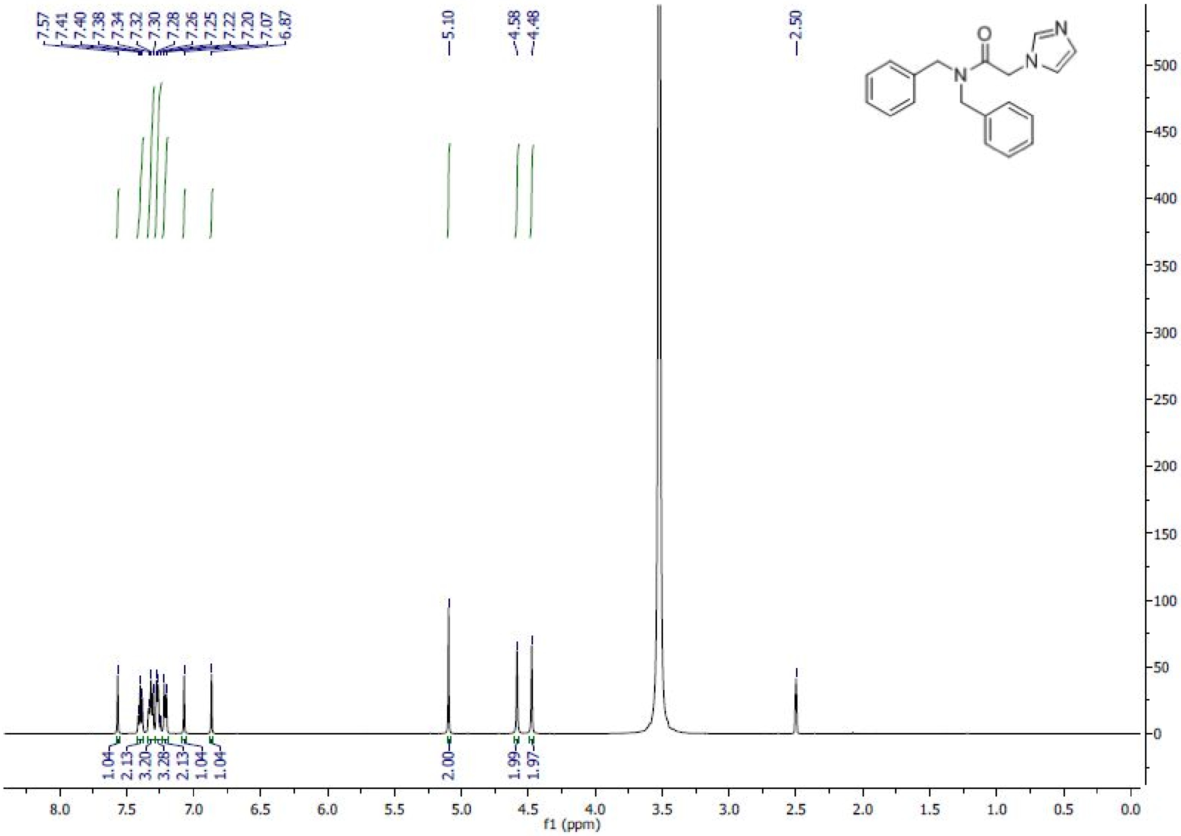

Figure S11. ^{1}H NMR (500 MHz, DMSO-d_6) of compound 7h.

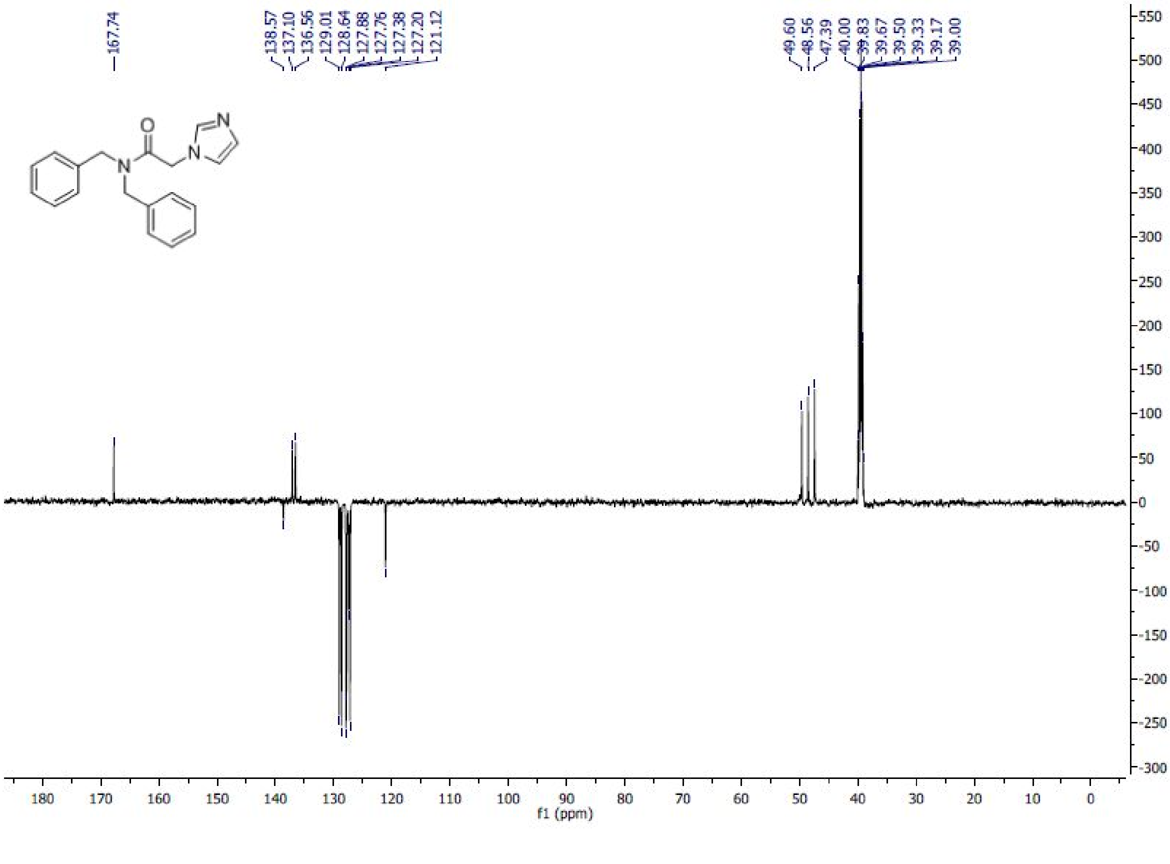

Figure S12. ¹³C NMR (125 MHz, DMSO-d_6) of compound 7h.

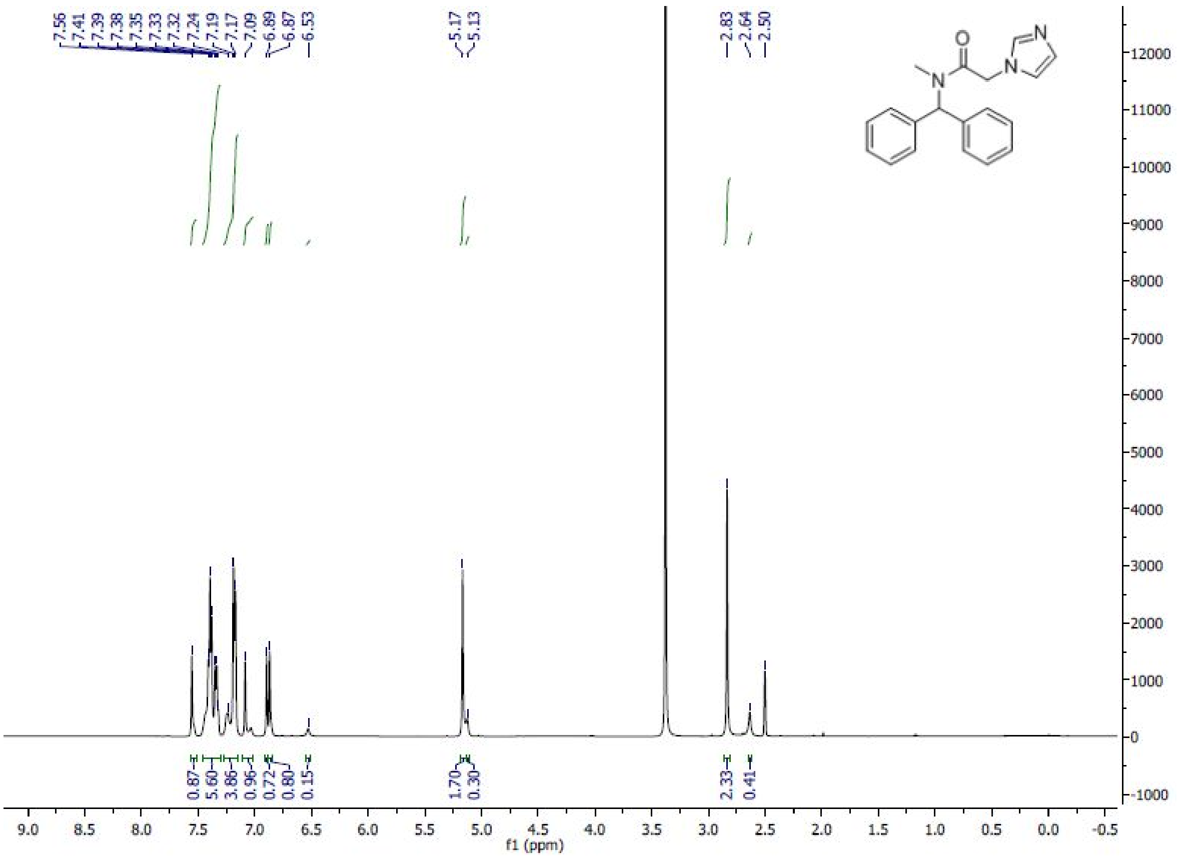

Figure S13. ¹H NMR (500 MHz, DMSO-d_6) of compound 7i.

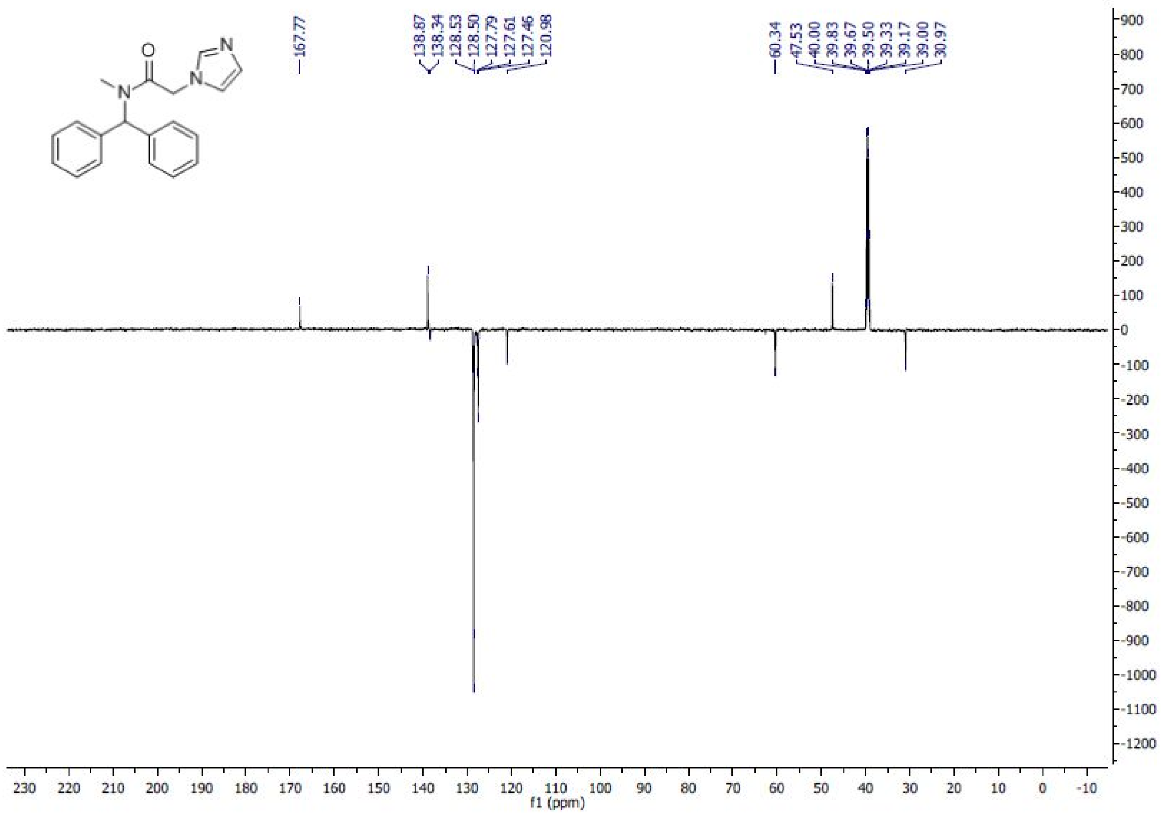

Figure S14. ¹³C NMR (125 MHz, DMSO-d_6) of compound 7i.

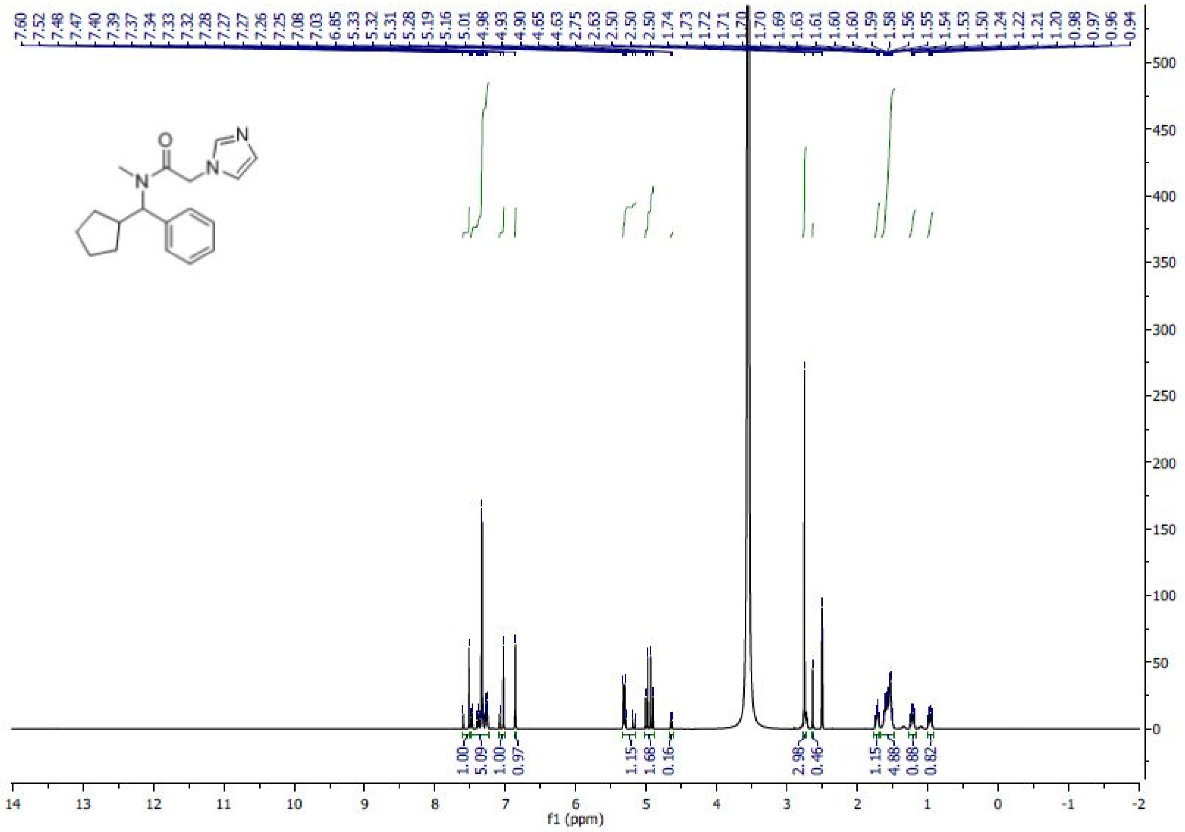

Figure S15. ¹H NMR (500 MHz, DMSO-d_6) of compound 7j.

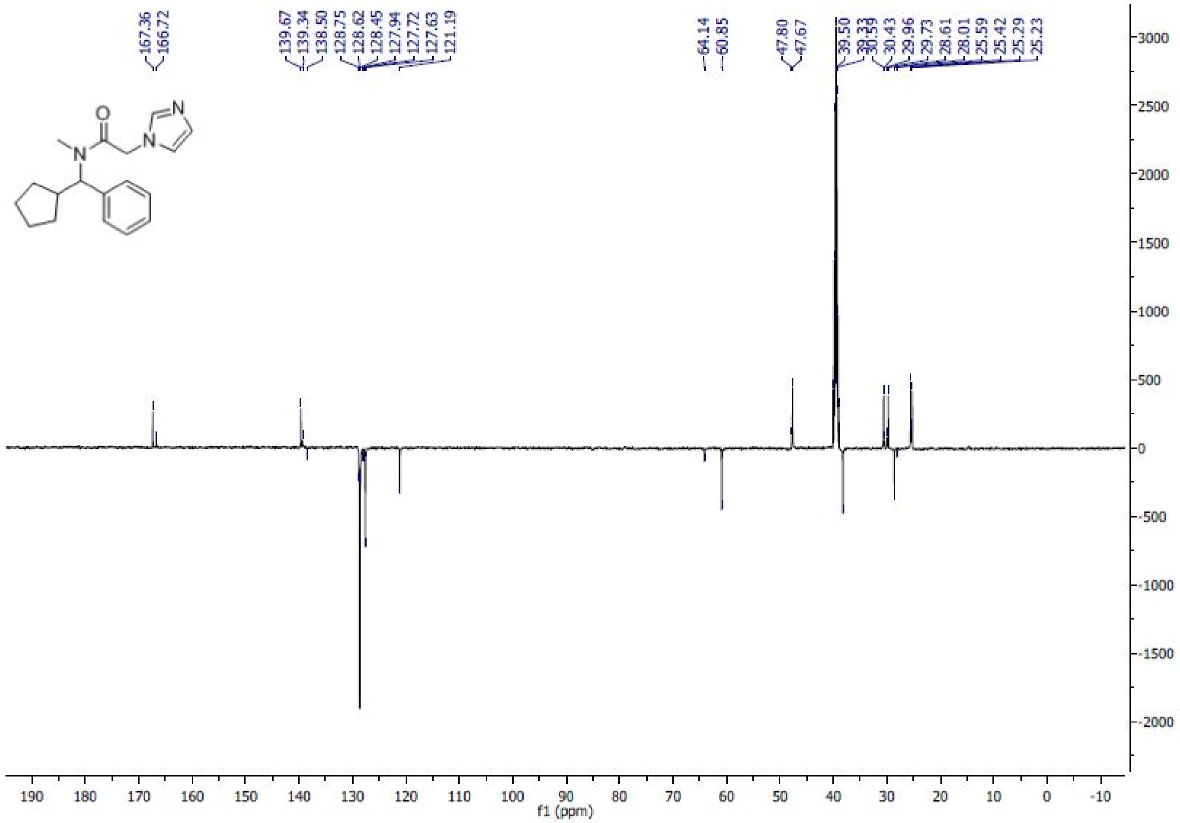

Figure S16. ^{13}C NMR (125 MHz, DMSO-d_6) of compound 7j.

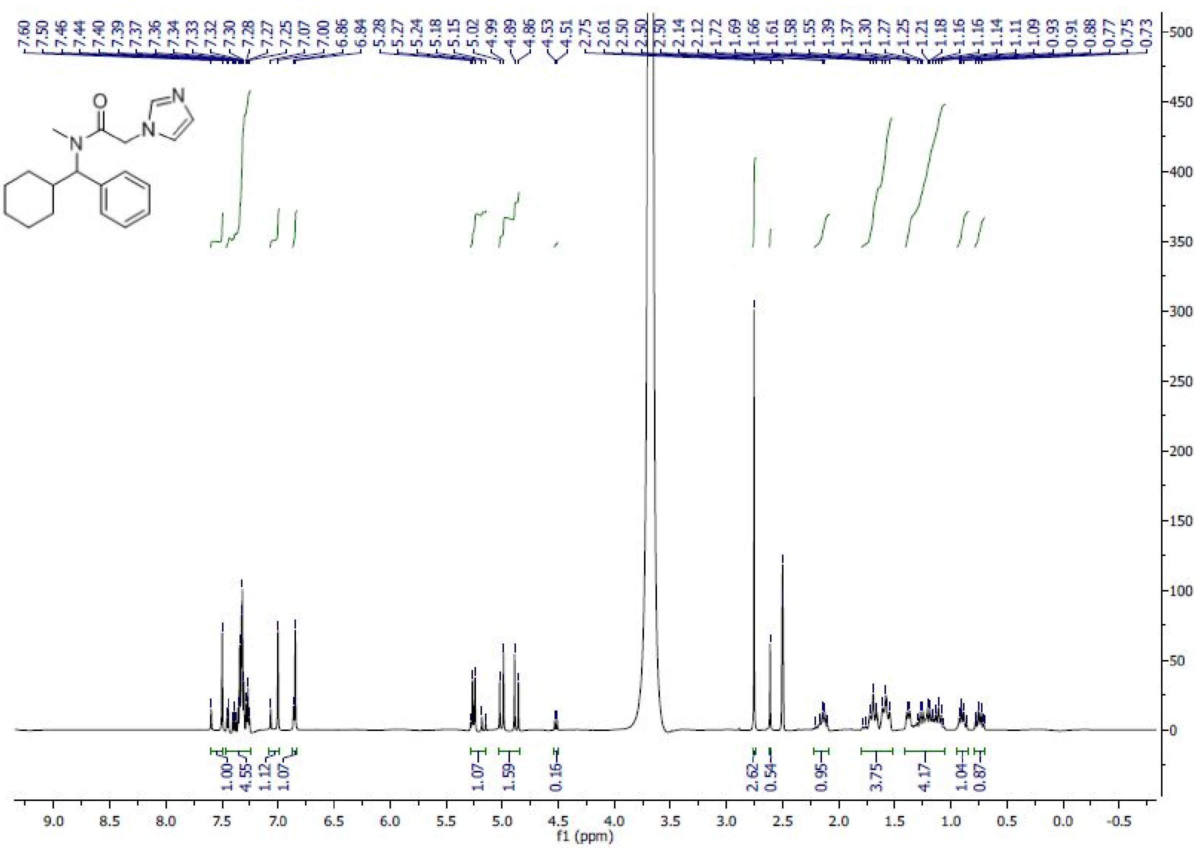

Figure S17. ^{1}H NMR (500 MHz, DMSO-d_6) of compound 7k.

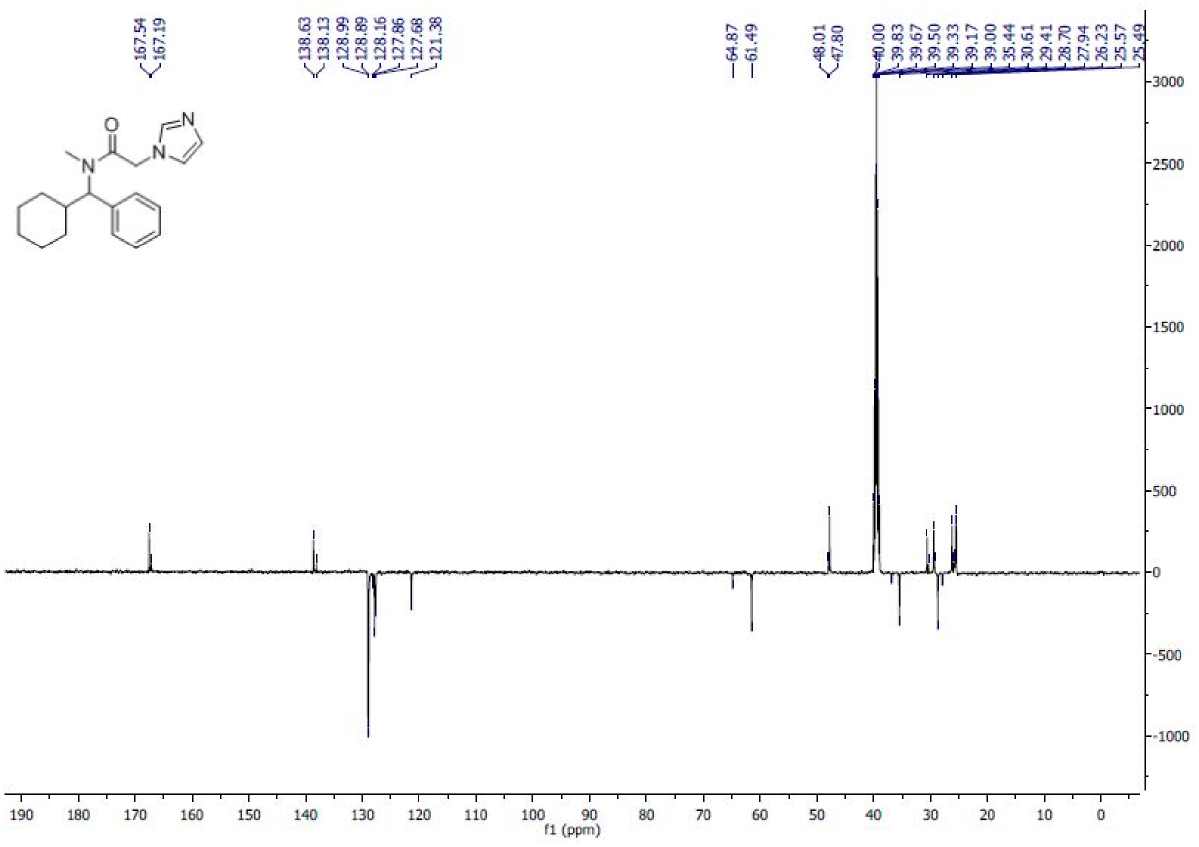

Figure S18. ^{13}C **NMR (125 MHz, DMSO-d_6) of compound 7k.**

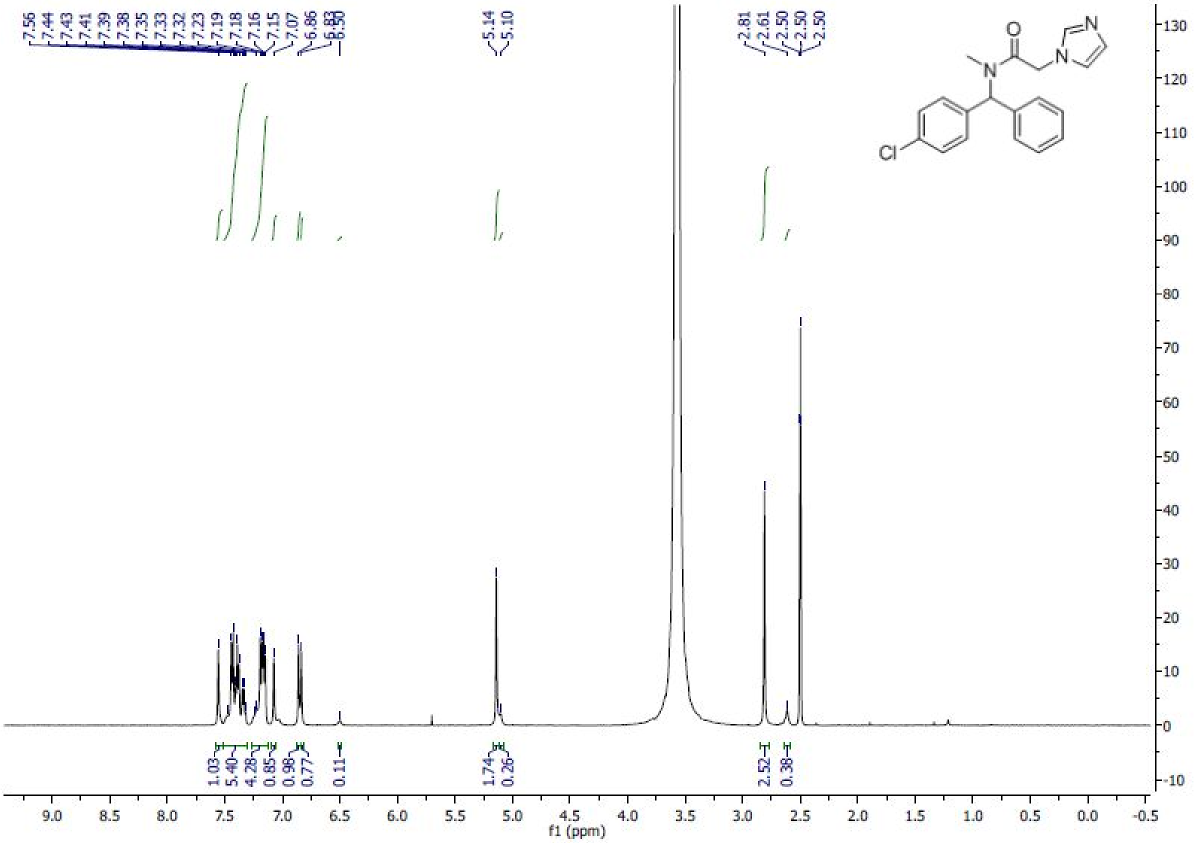

Figure S19. ^{1}H **NMR (500 MHz, DMSO-d_6) of compound 7l.**

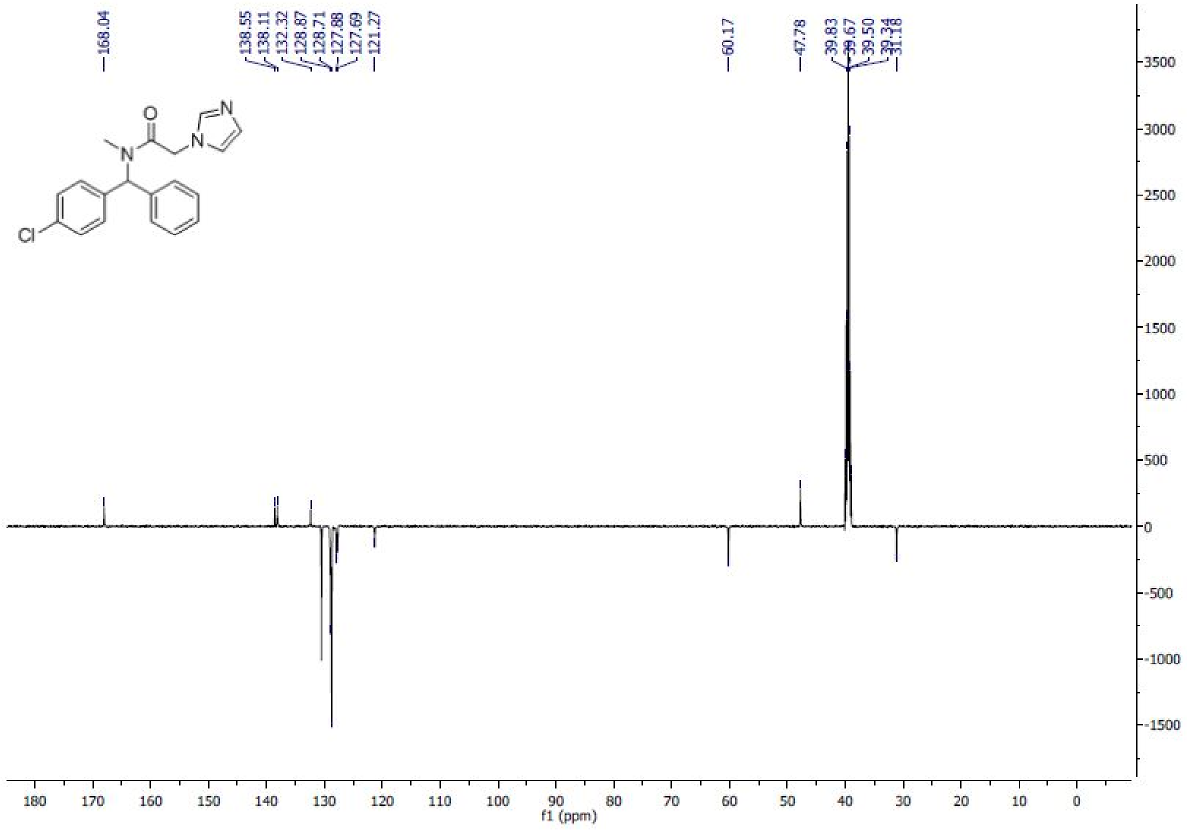

Figure S20. ^{13}C NMR (125 MHz, DMSO-d_6) of compound **7l**.

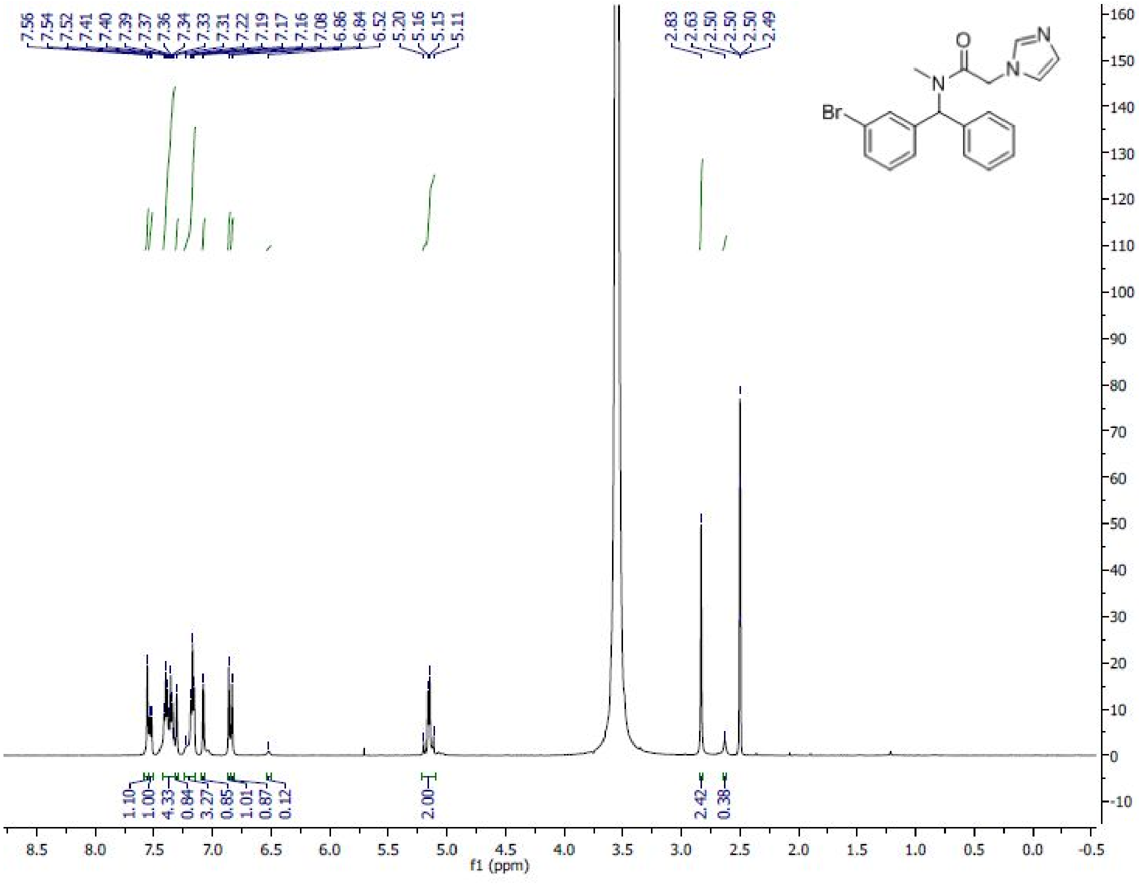

Figure S21. ^{1}H NMR (500 MHz, DMSO-d_6) of compound **7m**.

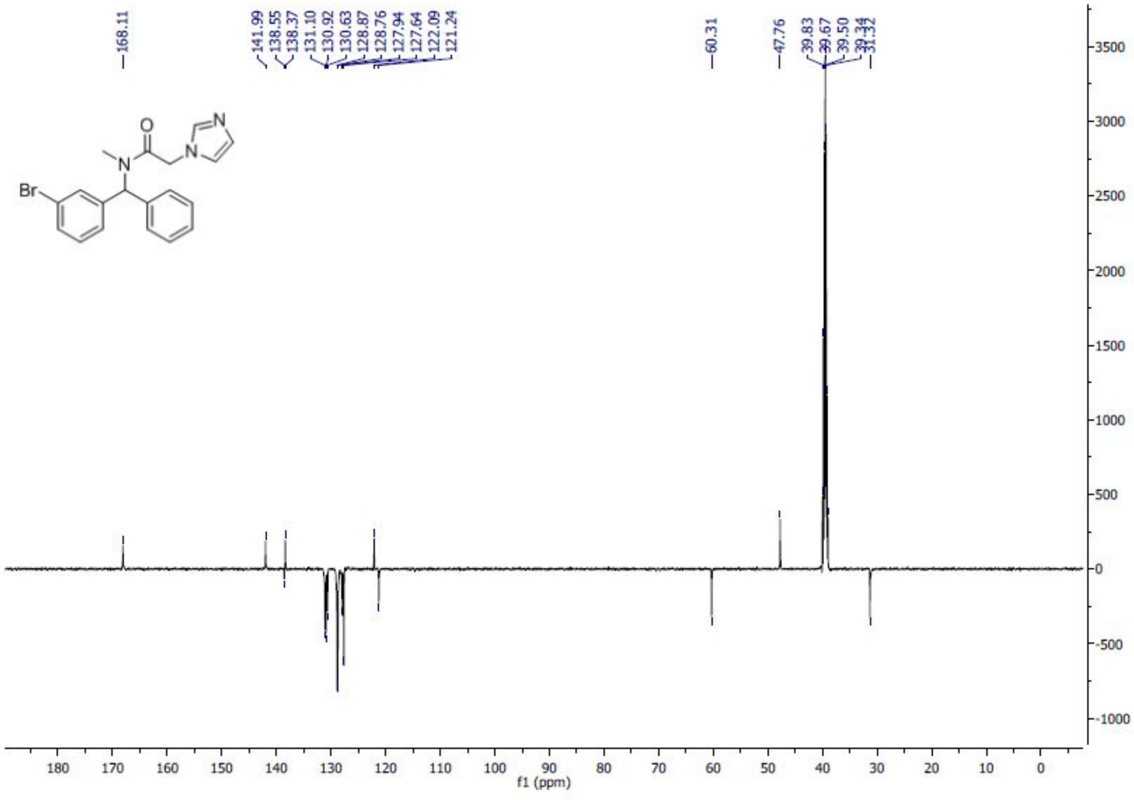

Figure S22. ¹³C NMR (125 MHz, DMSO-d_6) of compound 7m.

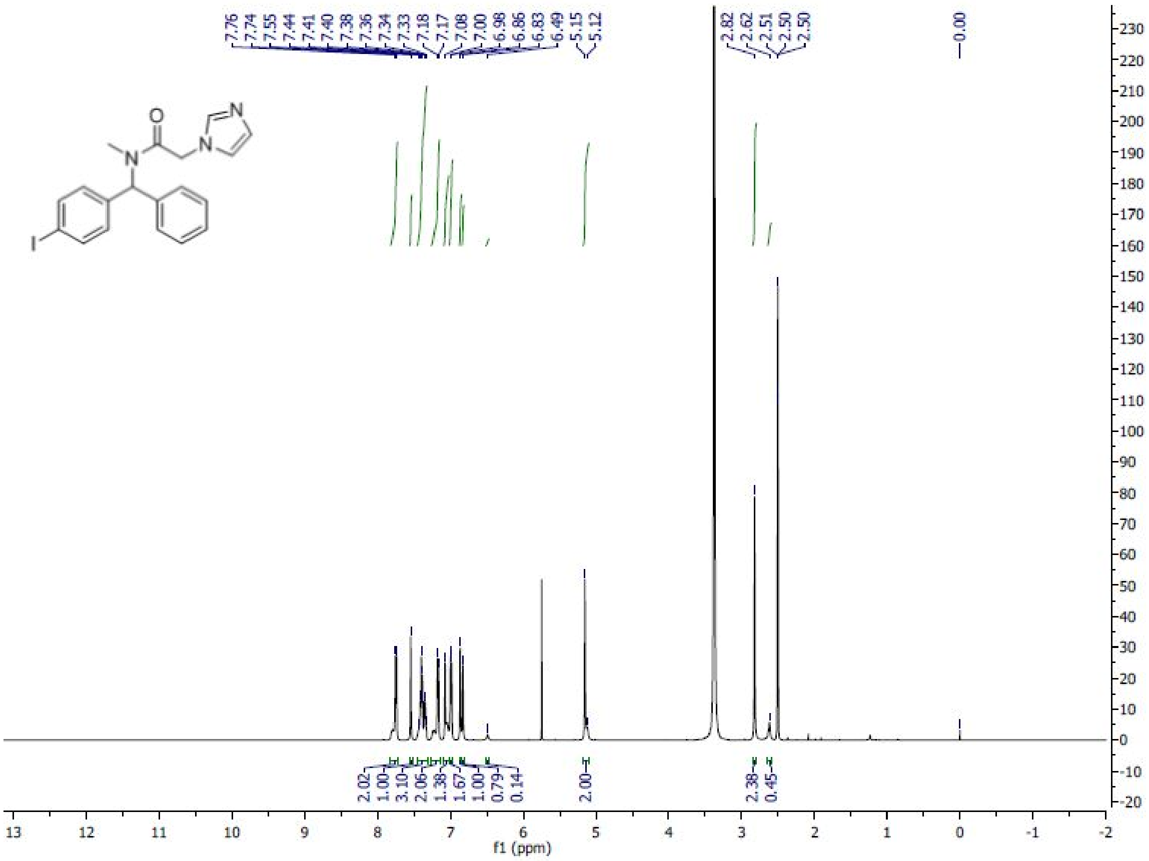

Figure S23. ¹H NMR (500 MHz, DMSO-d_6) of compound 7n.

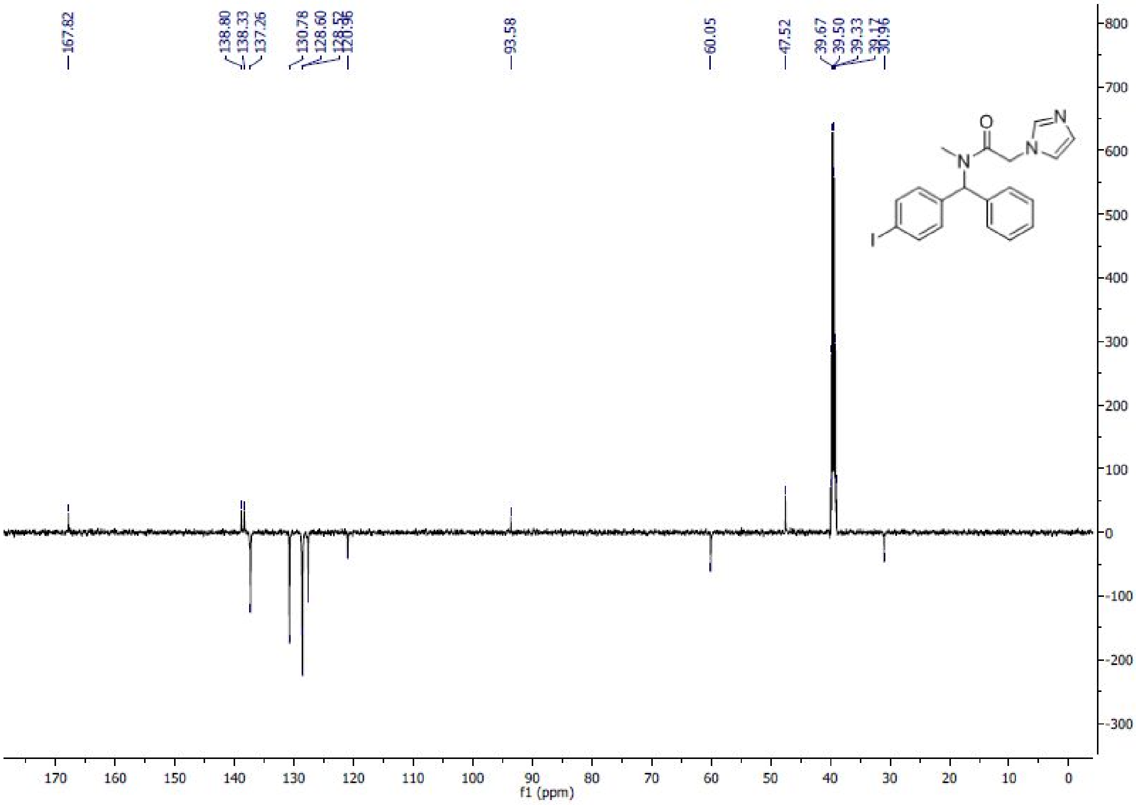

Figure S24. ^{13}**C NMR (125 MHz, DMSO-*d*$_6$) of compound 7n.**

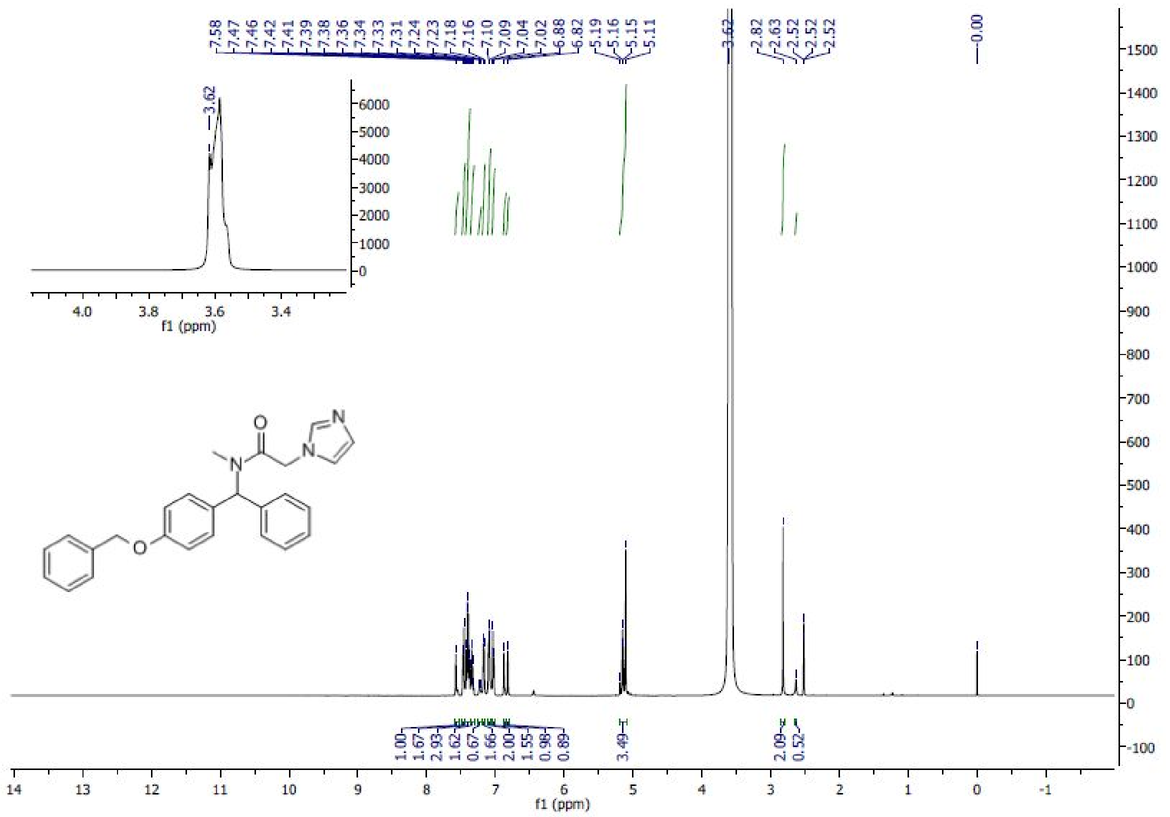

Figure S25. ^{1}**H NMR (500 MHz, DMSO-*d*$_6$) of compound 7o.**

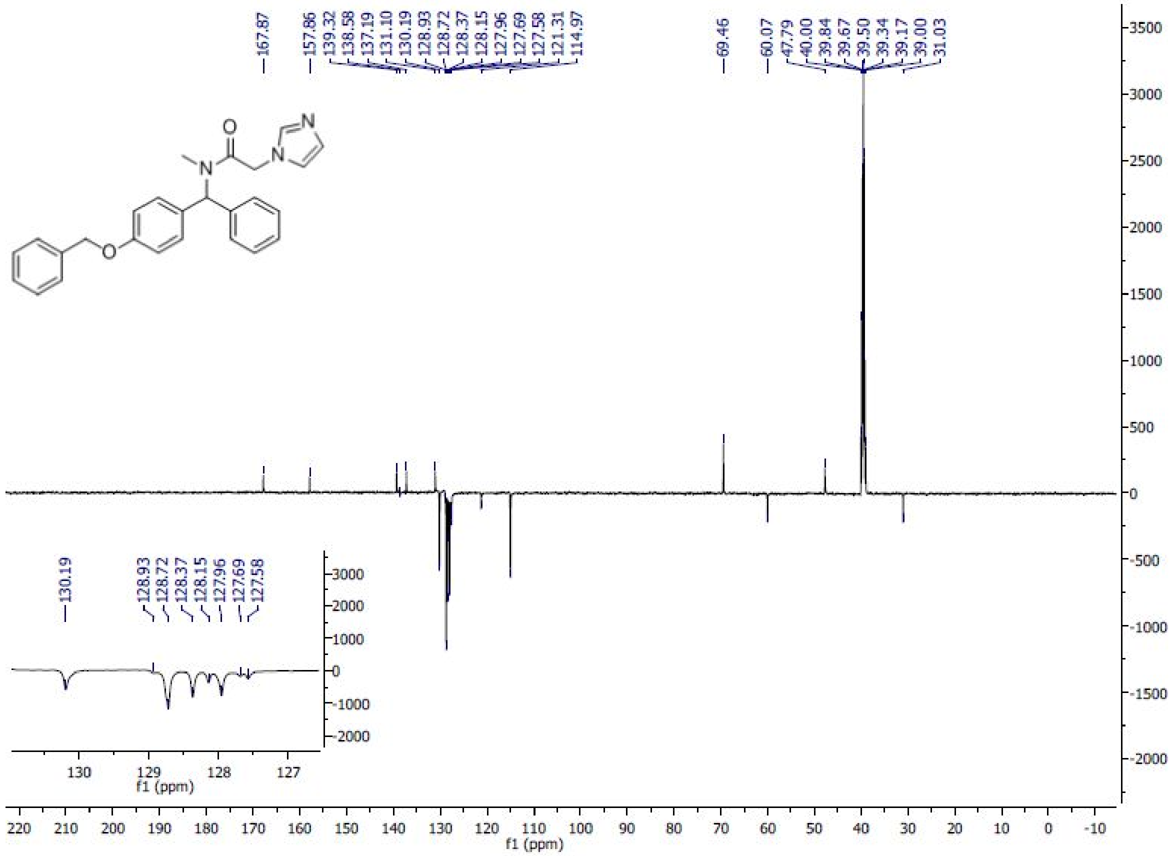

Figure S26. ^{13}C **NMR (125 MHz, DMSO-d_6) of compound 7o.**

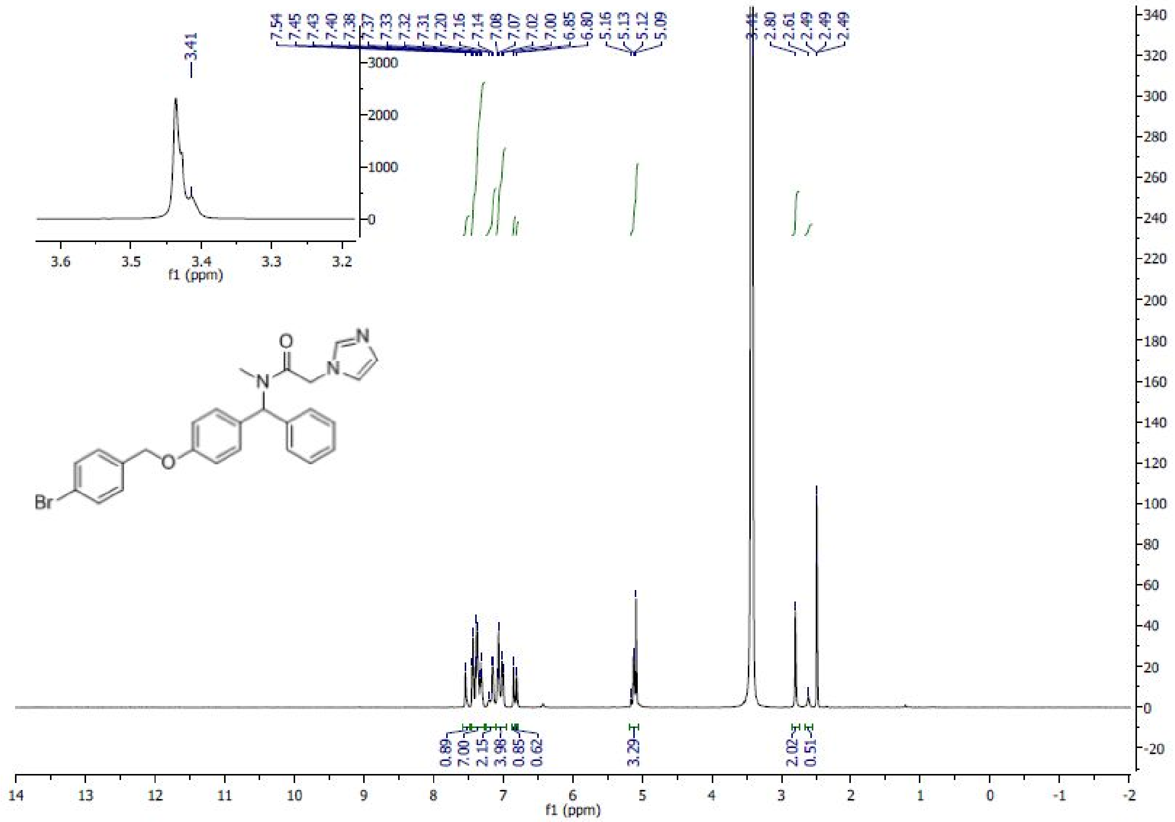

Figure S27. ^{1}H **NMR (500 MHz, DMSO-d_6) of compound 7p.**

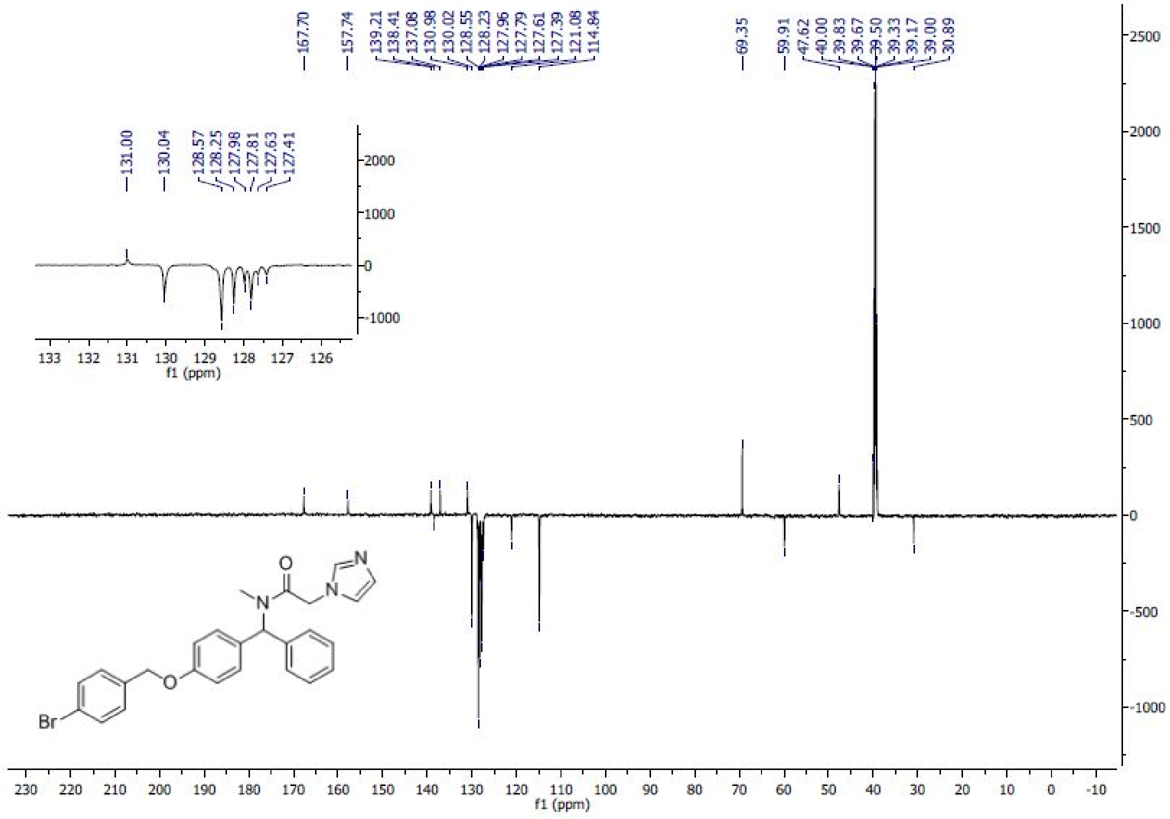

Figure S28. ^{13}C NMR (125 MHz, DMSO-d_6) **of compound 7p.**

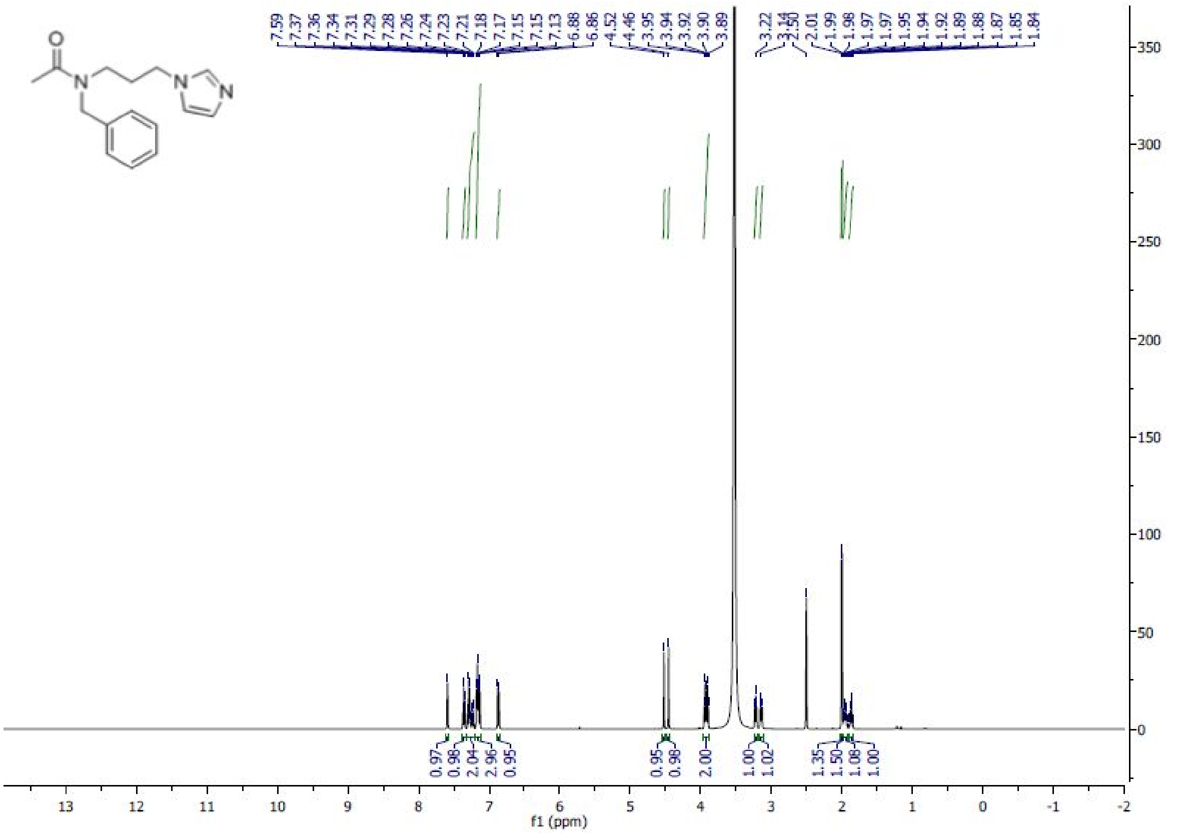

Figure S29. ^{1}H NMR (500 MHz, DMSO-d_6) **of compound 11a.**

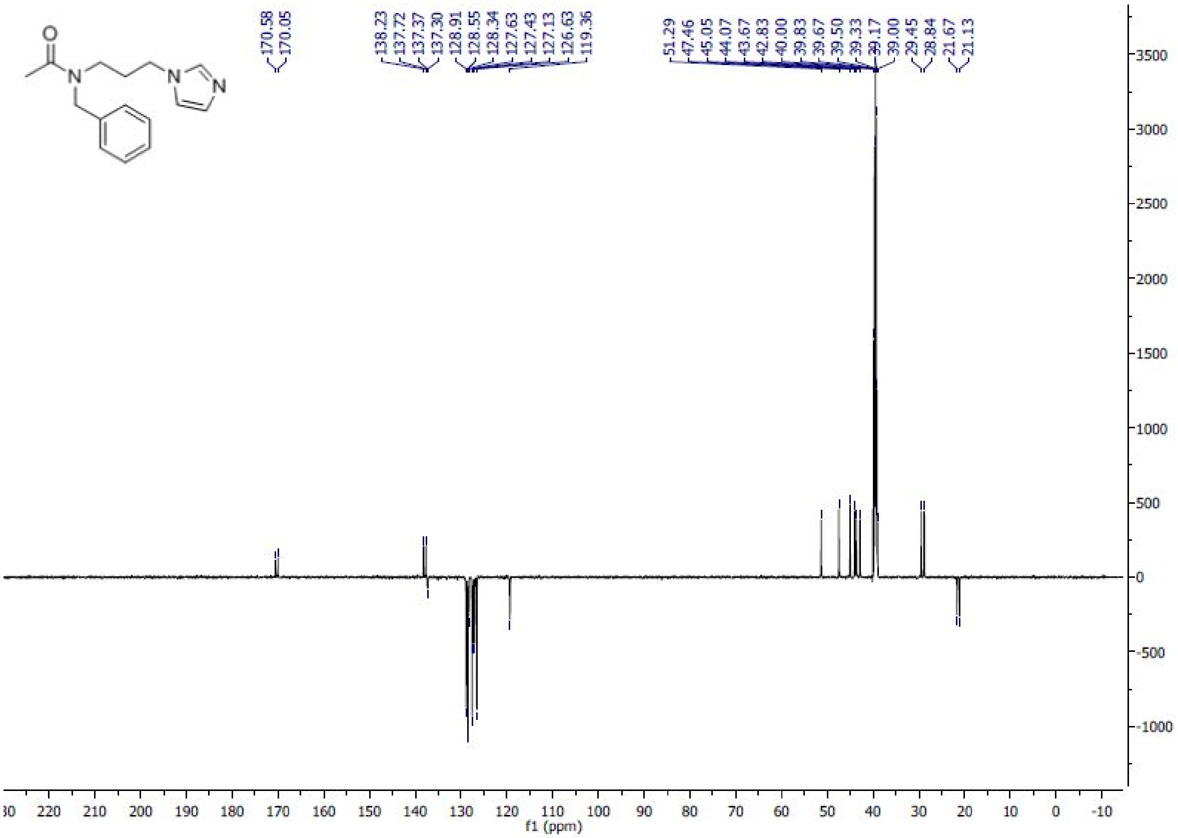

Figure S30. ^{13}C **NMR (125 MHz, DMSO-d_6) of compound 11a.**

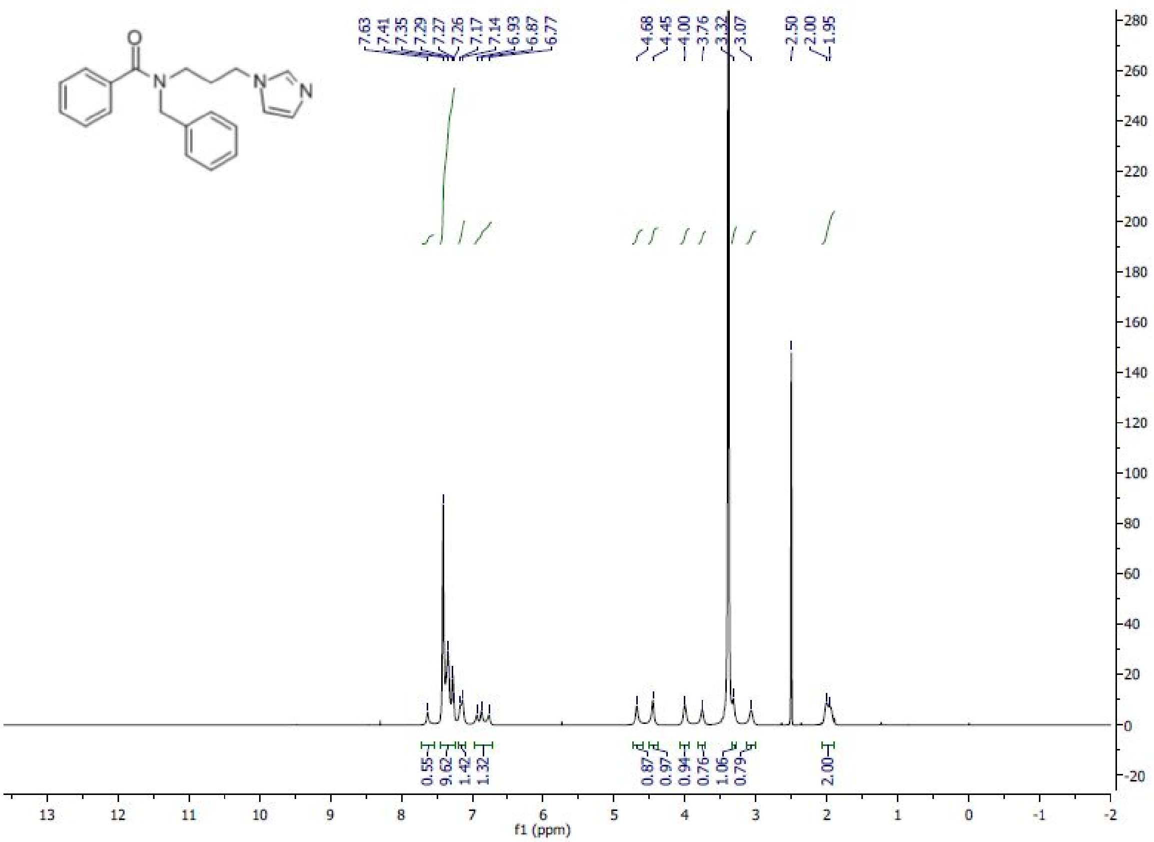

Figure S31. ^{1}H **NMR (500 MHz, DMSO-d_6) of compound 11b.**

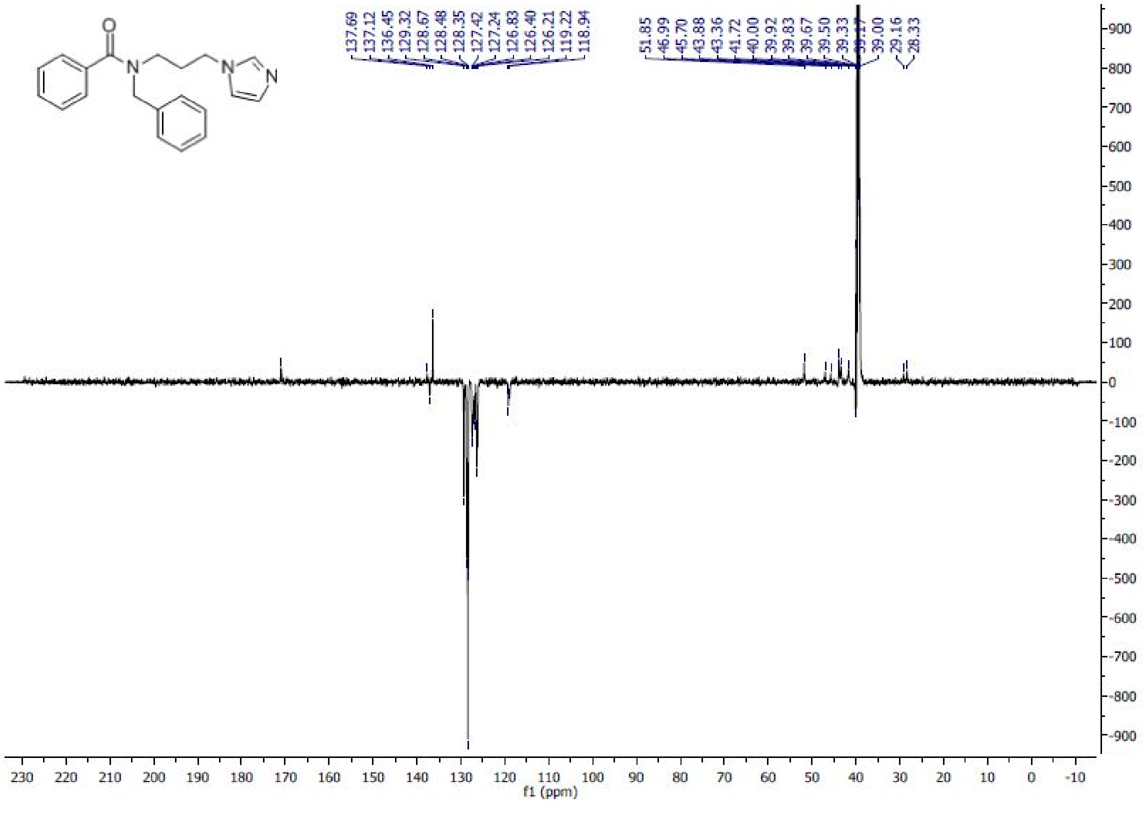

Figure S32. ¹³C NMR (125 MHz, DMSO-d_6) of compound 11b.

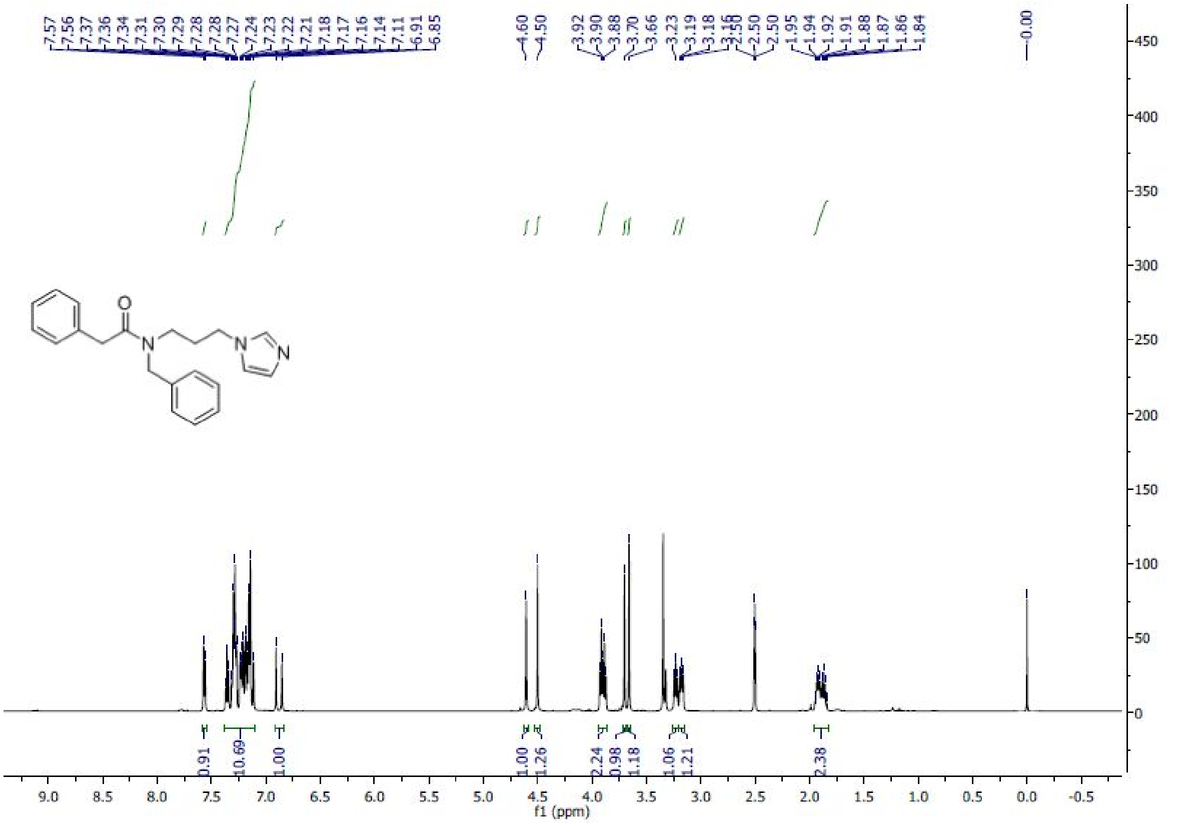

Figure S33. ¹H NMR (500 MHz, DMSO-d_6) of compound 11c.

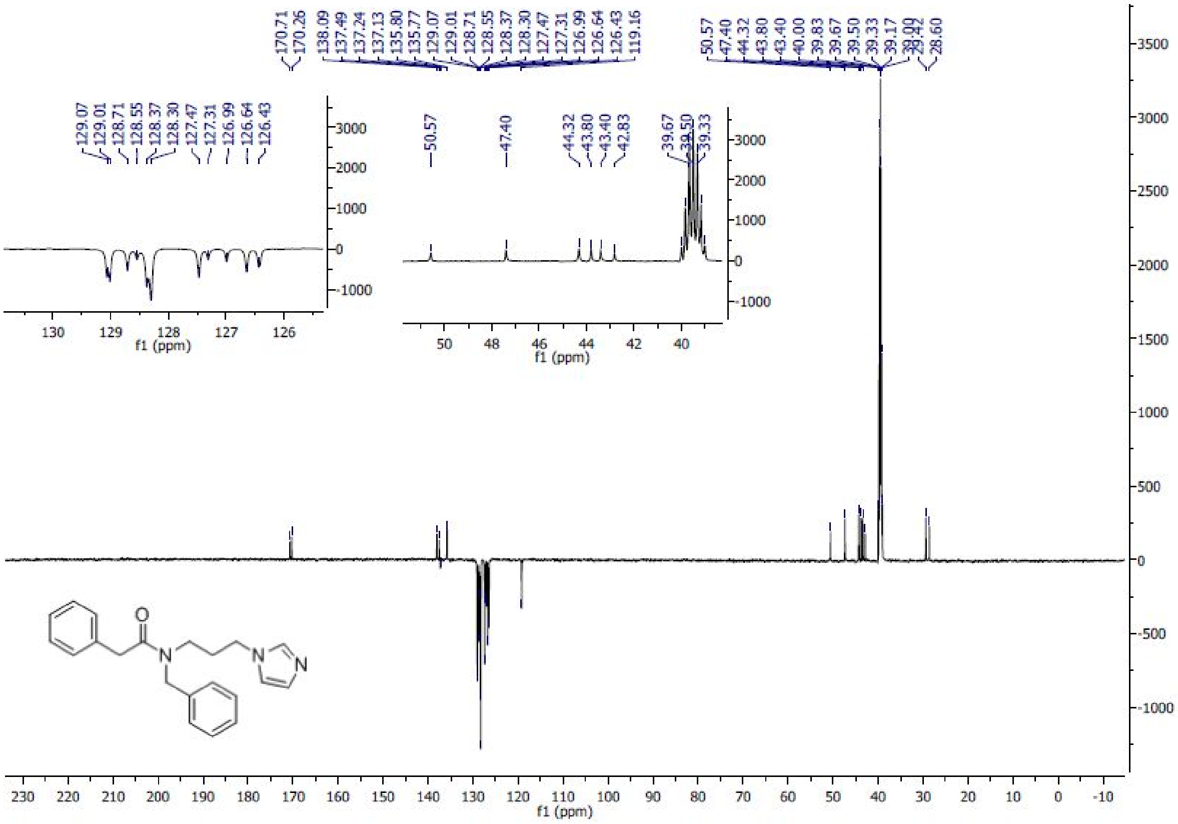

Figure S34. ^{13}**C NMR (125 MHz, DMSO-*d*$_6$) of compound 11c.**

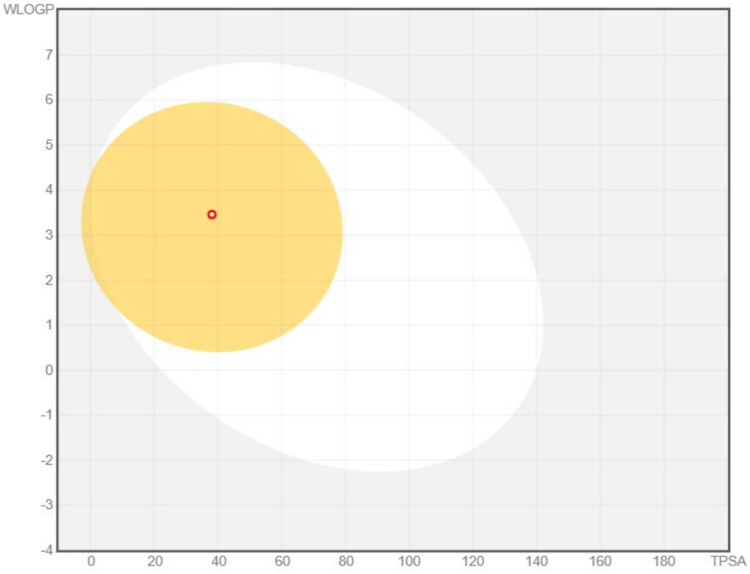

Figure S35. BOILED-Egg plot. Points located in the BOILED-Egg's yellow are the compounds predicted to permeate the BBB passively, differently the ones in the white are the molecules predicted to be only passively absorbed by the gastrointestinal tract. The red dot indicate that the compound is not transported by the P-glycoprotein.

Table S2. Results of SwissADME calculations.

Formula	$C_{19}H_{18}ClN_3O$
MW	339.82
#Heavy atoms	24
#Aromatic heavy atoms	17
Fraction Csp3	0.16
#Rotatable bonds	6
#H-bond acceptors	2
#H-bond donors	0

MR	94.99
TPSA	38.13
iLOGP	2.7
XLOGP3	3.46
WLOGP	3.46
MLOGP	2.55
Silicos-IT Log P	3.12
Consensus Log P	3.06
ESOL Log S	-4.25
ESOL Solubility (mg/ml)	1.89E-02
ESOL Solubility (mol/l)	5.56E-05
ESOL Class	Moderately soluble
Ali Log S	-3.94
Ali Solubility (mg/ml)	3.88E-02
Ali Solubility (mol/l)	1.14E-04
Ali Class	Soluble
Silicos-IT LogSw	-6.15
Silicos-IT Solubility (mg/ml)	2.39E-04
Silicos-IT Solubility (mol/l)	7.03E-07
Silicos-IT class	Poorly soluble
GI absorption	High
BBB permeant	Yes
Pgp substrate	No

CYP1A2 inhibitor	Yes
CYP2C19 inhibitor	Yes
CYP2C9 inhibitor	Yes
CYP2D6 inhibitor	Yes
CYP3A4 inhibitor	Yes
log Kp (cm/s)	-5.92
Lipinski #violations	0
Ghose #violations	0
Veber #violations	0
Egan #violations	0
Muegge #violations	0
Bioavailability Score	0.55
PAINS #alerts	0
Brenk #alerts	0
Leadlikeness #violations	0
Synthetic Accessibility	2.74

Table S3. Results of pkCSM calculations.

Property	Model Name	Predicted Value
Absorption	Water solubility	-3.102 log mol/L
Absorption	Caco2 permeability	1.82 log Papp in 10^{-6} cm/s
Absorption	Intestinal absorption (human)	94.698 % Absorbed
Absorption	Skin Permeability	-2.739 log Kp

Absorption	P-glycoprotein substrate	Yes
Absorption	P-glycoprotein I inhibitor	Yes
Absorption	P-glycoprotein II inhibitor	Yes
Distribution	VDss (human)	0.715
Distribution	Fraction unbound (human)	0.019
Distribution	BBB permeability	0.265 log BB
Distribution	CNS permeability	-1.482 log PS
Metabolism	CYP2D6 substrate	No
Metabolism	CYP3A4 substrate	Yes
Metabolism	CYP1A2 inhibitior	Yes
Metabolism	CYP2C19 inhibitior	Yes
Metabolism	CYP2C9 inhibitior	No
Metabolism	CYP2D6 inhibitior	Yes
Metabolism	CYP3A4 inhibitior	Yes
Excretion	Total Clearance	0.662 log ml/min/kg
Excretion	Renal OCT2 substrate	Yes
Toxicity	AMES toxicity	No
Toxicity	Max. tolerated dose (human)	0.854 log mg/kg/day
Toxicity	hERG I inhibitor	No
Toxicity	hERG II inhibitor	Yes
Toxicity	Oral Rat Acute Toxicity (LD50)	2.236 mol/kg
Toxicity	Oral Rat Chronic Toxicity (LOAEL)	1.246 log mg/kg_bw/day
Toxicity	Hepatotoxicity	No

Toxicity	Skin Sensitisation	No
Toxicity	T.Pyriformis toxicity	0.285 log ug/L
Toxicity	Minnow toxicity	1.058 log mM

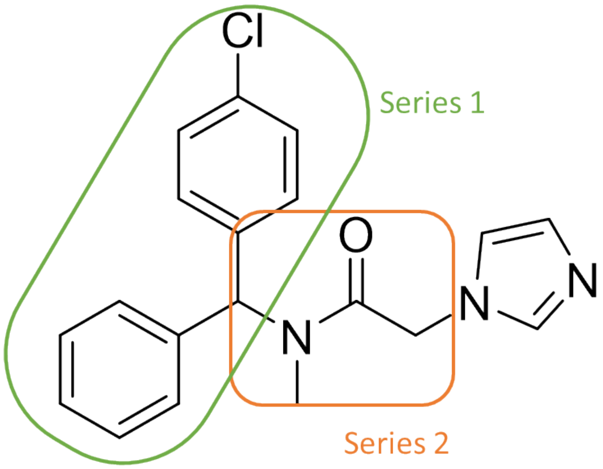

Figure S36. Series 1 and 2 of the scaffold-hopping analysis in 7l.

Table S4. Series 1 derived from isosteric replacement.

Entry	Structure	Predicted pIC_{50}
1	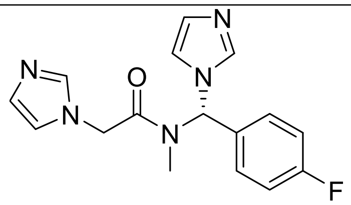	6.6
2	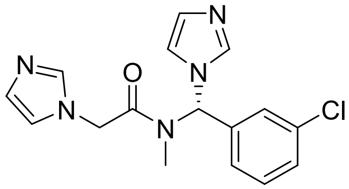	6.4

3	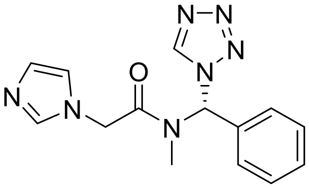	6.3
4	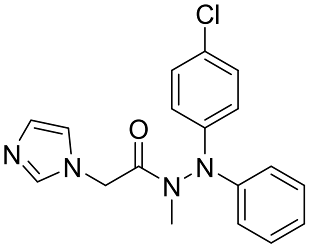	6.3
5	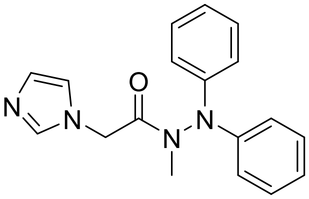	6.3
6	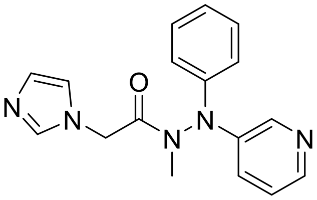	6.3
7	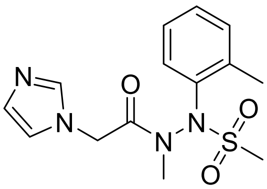	6.2
8	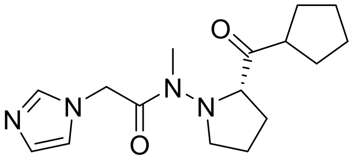	6.2
9	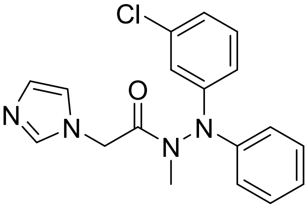	6.1

10	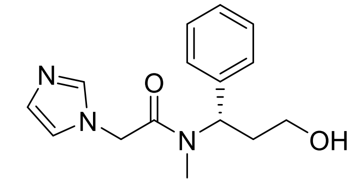	6.1

Table S5. Series 2 derived from isosteric replacement.

Entry	Structure	Predicted pIC_{50}
1	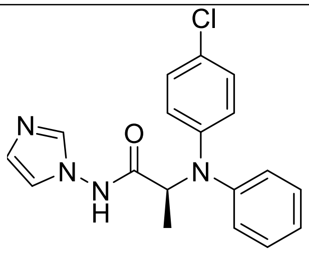	6.2
2	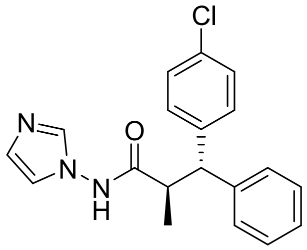	6
3	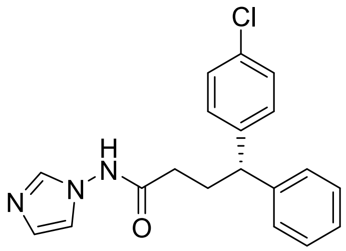	6
4	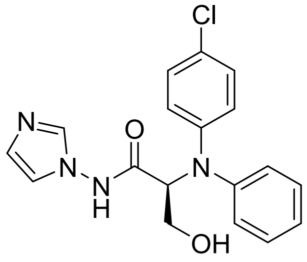	6

5	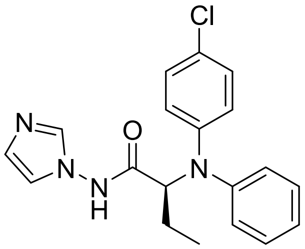	6
6	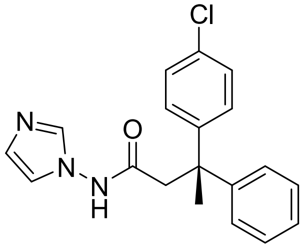	6
7	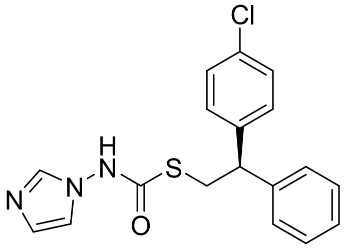	5.9
8	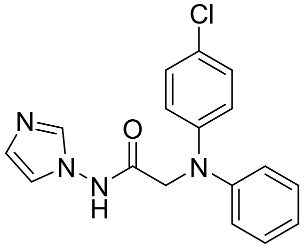	5.9
9	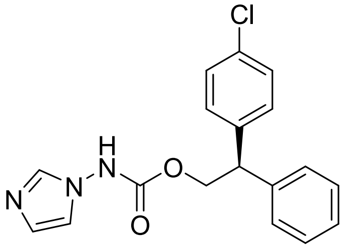	5.9
10	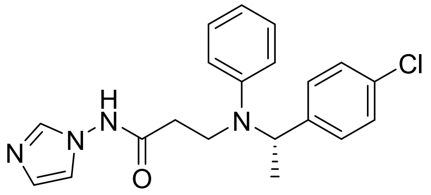	5.9
